# Differential expression analyses reveal extensive transcriptional plasticity induced by temperature in New Zealand silver trevally (*Pseudocaranx georgianus*)

**DOI:** 10.1111/eva.13332

**Published:** 2022-01-22

**Authors:** Noemie Valenza‐Troubat, Marcus Davy, Roy Storey, Matthew J. Wylie, Elena Hilario, Peter Ritchie, Maren Wellenreuther

**Affiliations:** ^1^ The New Zealand Institute for Plant and Food Research Limited Nelson New Zealand; ^2^ The New Zealand Institute for Plant and Food Research Limited Te Puke New Zealand; ^3^ School of Biological Sciences Victoria University of Wellington Wellington New Zealand; ^4^ School of Biological Sciences University of Auckland Auckland New Zealand

**Keywords:** aquaculture, climate change, fisheries management, phenotypic plasticity, transcriptomics

## Abstract

Ectotherm species, such as marine fishes, depend on environmental temperature to regulate their vital functions. In finfish aquaculture production, being able to predict physiological responses in growth and other economic traits to temperature is crucial to address challenges inherent in the selection of grow‐out locations. This will become an even more significant issue under the various predicted future climate change scenarios. In this study, we used the marine teleost silver trevally (*Pseudocaranx georgianus*), a species currently being explored as a candidate for aquaculture in New Zealand, as a model to study plasticity in gene expression patterns and growth in response to different temperatures. Using a captive study population, temperature conditions were experimentally manipulated for 1 month to mimic seasonal extremes. Phenotypic differences in growth were measured in 400 individuals, and gene expression patterns of pituitary gland and liver were determined in a subset of 100 individuals. Results showed that growth increased 50% in the warmer compared with the colder condition, suggesting that temperature has a large impact on metabolic activities associated with growth. A total of 265,116,678 single‐end RNA sequence reads were aligned to the trevally genome, and 28,416 transcript models were developed (27,887 of these had GenBank accessions, and 17,980 unique gene symbols). Further filtering reduced this set to 8597 gene models. 39 and 238 differentially expressed genes (DEGs) were found in the pituitary gland and the liver, respectively (|log_2_FC| > 0.26, *p*‐value < 0.05). Of these, 6 DEGs showed a common expression pattern between both tissues, all involved in housekeeping functions. Temperature‐modulated growth responses were linked to major pathways affecting metabolism, cell regulation and signalling, previously shown to be important for temperature tolerance in other fish species. An interesting finding of this study was that genes linked to the reproductive system were up‐regulated in both tissues in the high treatment, indicating the onset of sexual maturation. Few studies have investigated the thermal plasticity of the gene expression in the main organs of the somatotropic axis simultaneously. Our findings indicate that trevally exhibit substantial growth differences and predictable plastic regulatory responses to different temperature conditions. We identified a set of genes that provide a list of candidates for further investigations for selective breeding objectives and how populations may adapt to increasing temperatures.

## 1 INTRODUCTION

Ectotherm species, such as most marine fishes, depend on temperature to maintain homeostasis, which makes them particularly sensitive to temperature fluctuations in their environment (Ficke et al., 2007). This is because the metabolic demand of these species is in part determined by the thermal environmental conditions (Brett & Groves, 1979), which in turn influences physiological processes such as growth and reproduction (Fry, 1971; Gobler et al., 2018; Hochachka & Somero, 2002). Like all organisms, teleost fish have adapted to thrive within a certain range of temperatures but these ranges vary between species and life stages (Dahlke et al., 2020; Pörtner & Farrell, 2008). When exposed to temperatures outside of that range, individuals become stressed and suffer a reduction in performance. When temperatures rise above the optimal limit, metabolic costs rise (Sebens, 1987) and oxygen transport to tissues becomes limited (Ern et al., 2016). Below the optimal range, metabolic costs are lower but general activities, such as prey capture and digestion, decrease, which can negatively affect the accumulation of sustenance and energy. A lack of energy means a species is unable to sustain growth and other critical functions (Sebens, 1987). For example, cold exposure has been shown to decrease motility performances between 10% and 55% in juvenile silver perch (*Bidyanus bidyanus*; Parisi et al., 2020).

Phenotypic plasticity, or the capacity of a genotype to produce different phenotypes when exposed to different environments, enables organisms to adjust their physiology in response to different thermal conditions. This coping mechanism can happen at different stages of life (Fu et al., 2019; Sandblom et al., 2014): developmental plasticity is often irreversible whereas plastic responses later in life, often referred to as acclimation, can be reversible if exposure to environmental variables is ended (Beaman et al., 2016). One of the critical intermediate steps of this mechanism is the induction of changes in gene expression levels (Hodgins‐Davis & Townsend, 2009). This allows molecular and physiological pathways to be regulated to adjust to a new environment and continue to maintain a high level of fitness (Beaman et al., 2016). Studying expression plasticity thus provides insights into how different phenotypes are generated by the interactions between genotype and the environment (Huang & Agrawal, 2016). Moreover, the study of the expression levels of genes allows the examination of plastic responses for a large set of traits (transcripts levels) that are relatively unbiased compared with traditional phenotypic traits, which can be skewed because of either preconceived notions of their importance or ease of measurement.

Growth in teleost fish is mainly regulated by the somatotropic axis (hypothalamus–pituitary–liver; Reinecke, 2010). Some of the major hormones involved in this axis are somatostatin (Ss) and growth hormone‐releasing hormone (Ghrh), which are secreted by the hypothalamus. Growth hormone (Gh) is secreted by the pituitary gland (referred to as pituitary hereafter) and binds to its receptor (Ghr) present at the surface of target organs, such as the liver. This stimulates the release of insulin‐like growth factor‐I (Igf‐I; Fu et al., 2016; Norbeck et al., 2007) and activates the metabolism of fats, carbohydrates and proteins (Norbeck et al., 2007). Although the function of these hormones has been well studied in some model fish species (Duan et al., 2010), few studies have investigated the thermal phenotypic plasticity of their gene expression in the main organs of the somatotropic axis simultaneously.

The regulation of the genes involved in temperature‐related growth responses can be studied with gene expression studies to gain insights into the molecular machinery underlying these responses. Temperature‐mediated gene expression changes have been observed in several fish species (Metzger & Schulte, 2018; Smith et al., 2013; Veilleux et al., 2015). Genetic variability has been showed to affect the plastic response within populations such as in the Australasian snapper (*Chrysophrys auratus*; Wellenreuther et al., 2019) or in the three‐spined stickleback (*Gasterosteus aculeatus*; Metzger & Schulte, 2018), as well as between species (Baumann & Conover, 2011; Haugen & Vøllestad, 2000; Hutchings, 2011; Jensen et al., 2008). Consequently, each new species investigated has a potentially unique plastic response to temperature changes, which can itself vary among populations.

In this study, we used the New Zealand silver trevally (*Pseudocaranx georgianus*) as an aquaculture model to investigate transcriptomic responses to two temperature treatments. In Aotearoa, its Māori name is araara. Indigenous Māori people have a strong cultural connection to trevally, where it is considered as taonga (i.e. has value, or is treasured). This species is currently being explored as a future candidate species for commercial aquaculture in New Zealand, and basic biological data about the species’ life history are provided in Valenza‐Troubat et al. (2021). Species of the *Pseudocaranx* genus appear to do well in farm‐like conditions, as demonstrated by long‐lasting successful cultures of striped jack (*P*. *dentex*) in Japan where it has been bred since the 1990s (Fukusho, 1995). However, compared with its sister species who have faster growth rates (Abbink et al., 2012), trevally is much slower, which means that its growth rate will likely need to be improved, and genetic information regarding the genes involved and their regulation, would provide crucial context for a future selective breeding programme on this species to enhance growth and to select the appropriate on‐growing sea‐pen locations around New Zealand to maximize growth gains. In the present study, we are using a replicated tank array; we experimentally measured temperature‐dependent growth of 400 juvenile, sexually immature trevally over 1 month, and used high‐throughput RNA‐sequencing (RNA‐seq) to compare differential gene expression responses in the pituitary and the liver in a subset of 100 individuals. The specific objectives of this study were to (1) measure growth responses following exposure to warm/cold temperatures; (2) quantify the expression changes of all genes in the pituitary and liver at different temperatures; and (3) annotate the differentially expressed genes in pituitary and liver to understand the potential molecular mechanisms regulating growth under different temperature.

## 2 METHODS

### 2.1 Fish holding procedures, experimental set‐up and trait phenotyping

**FIGURE 1:**
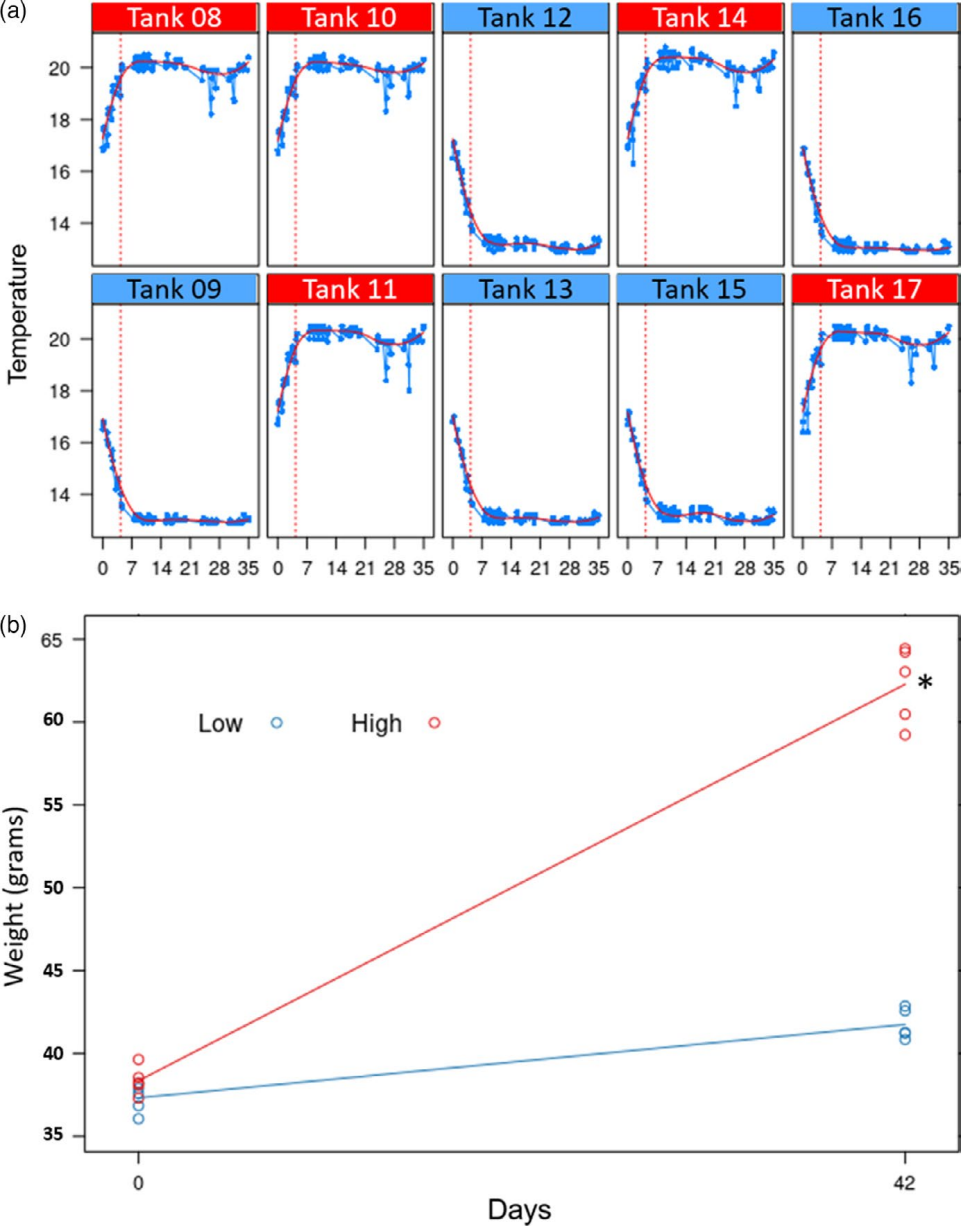
Experimental temperatures and physiological data. (a) Temperature of high (red) and low (blue) treatments recorded in the replicate tanks throughout the experiment. (b) Average weights in the high (red line) and low (blue line) treatments recorded at the beginning and at the end of the experiment. Each dot represents the average weight in a replicate tank. The asterisk shows a statistically significant difference between means (*p*‐value < 0.01)

The New Zealand Institute for Plant and Food Research Limited (PFR) has been holding trevally since 2015, and in 2016, a selective breeding programme was started to select for enhanced growth (Valenza‐Troubat et al., 2021). As part of this, an F_1_ generation was produced in December 2018 at the Finfish Facility of the Nelson Research Centre in New Zealand, in December 2018 using a wild caught F_0_ broodstock. A subset of this generation was randomly selected for this experiment. Prior to experimentation, the F_1_ cohort was kept together in a 30,000‐L tank and provided with flow‐through filtered seawater at ambient temperature and light conditions. At the start of the experiment, in May 2019, 5‐month‐old fish were anaesthetized (10 mg/L AQUI‐S^®^, AQUIS NZ Ltd) and weighed. Four hundred individuals were randomly assigned to a high (20°C) or a low (13°C) temperature treatment (*n* = 200 per treatment) and divided into five replicate tanks per treatment (*n* = 40 per replicate). The tank allocation was randomized to avoid any spatial effects within the bay the experiment took place in. Fish were acclimatized at ambient temperature (16°C ± 0.9) for a period of 10 days following their tank assignment. The temperature was then increased or decreased 1°C per day over a period of 5 days, until treatment temperatures of either 20.0 or 13.0°C were reached, henceforth referred to as high and low temperature treatments, respectively. These temperatures mimic those found in the Tasman Sea during winter and summer. Temperature loggers (HOBO, Onset Computer Corporation) were used to monitor each tank and individual heaters were used to adjust the temperatures if needed. The low treatment had a mean temperature of 13.01°C and the high treatment had a mean temperature of 20.02°C, with minimal variation across the experiment (absolute maximum differences in the low and high treatments were ±1.7 and ±0.5°C, respectively, Figure 1a). The fish were maintained in 800‐L tanks supplied with 1 µm filtered, UV filtered, recirculating aerated seawater (5 L/min flow), and fed daily with commercial pellets (3 mm, Nova ME; Skretting), at a ration equivalent to 2% body mass, spread across four feeds per day. Dissolved oxygen levels were checked four times per day to ensure levels were kept at >90% and tanks were cleaned every 3–4 days. The temperature conditions were then maintained for 32 days. Upon completion of the trial, a total of 100 fish (50 per treatment, 10 per replicate) were anaesthetized (25 ppm AQUI‐S^®^) and then netted from their tank and subsequently sacrificed using an overdose of anaesthetic (60 ppm AQUI‐S^®^). Immediately following euthanasia, fish were weighed again and dissected immediately to snap‐freeze pituitary and liver tissues.

### 2.2 RNA extraction, Illumina library preparation and sequencing

Variations in temperature have been shown to differentially induce expression changes in different tissue types (Metzger & Schulte, 2018; Wellenreuther et al., 2019). We selected the pituitary as the endocrine gland of the somatotropic axis, which regulates growth in teleost (Pérez‐Sánchez et al., 2018) and the liver as its main target organ. Total RNA of both tissues was extracted separately for each individual using the NucleoSpin^®^ RNA kit (Macherey‐Nagel), in accordance with the manufacturer's instructions, which included an on‐column DNase digestion. Due to size differences, total RNA was eluted in 40 μl of TE buffer for the pituitary samples and 50 μl for the liver samples. RNA integrity was checked by capillary electrophoresis with the Fragment Analyzer and the HS total RNA kit (Agilent Technologies). Normalized starting quantities of total RNA (164 and 227 ng for ng for the pituitary and the liver, respectively) were used to prepare 200 Illumina sequencing libraries with the QuantSeq™ 3′ mRNA‐seq library kit plus Dual PCR add on extra barcodes (Lexogen). Library preparations were performed as per the manufacturer's instructions. Sequencing was performed by the Australian Genome Research Facility (AGRF), 100 bp single‐end reads on three lanes of NovaSeq (Illumina).

### 2.3 Genome annotation and completion rate

The trevally genome assembly and its quality are described in detail in Catanach et al. (2021). Briefly, it was outsourced to DoveTail Genomics, who provided a Meraculous (Chapman et al., 2011) de novo assembly from Chicago and Dovetail Hi‐C data, by scaffolding first with the Chicago data plus HiRise. The resulting assembly was used as input into the HiRise pipeline along with the Dovetail Hi‐C data. For this analysis, de novo repeats were identified using RepeatModeler version 1.0.11 (Smit et al., 2015) with the search NCBI engine. Repeats were classified by RepeatModeler into simple, tandem and interspersed repeats. A total of 12.8% of the genome was masked for repeats using RepeatMasker version 4.0.5 (Smit, 2004). Automated gene models were predicted with the BRAKER2 pipeline version 2.1.0 (Hoff et al., 2018), using trevally paired‐end RNA sequences sourced from seven separate tissues (skin, white muscle, liver, kidney, gill, heart and brain) and the trevally genome as input. Gene completion levels were evaluated in the genome assembly using BUSCO version 3.0.2 (Simão et al., 2015; Waterhouse et al., 2018) with the lineage set vertebrata_odb9 to assess completeness of single‐copy orthologues. E‐utilities version 11.4 (https://ftp.ncbi.nlm.nih.gov/entrez/entrezdirect) was used to download protein sequences for zebrafish (*Danio rerio*; 88,504 sequences) and yellowtail kingfish (*Seriola lalandi*; 39,513 sequences) from NCBI (https://www.ncbi.nlm.nih.gov/) on 7 September 2020. BLASTX version 2.2.26 (Altschul et al., 1997) was used to search downloaded protein sequences using a translated nucleotide query for each transcriptome gene locus model. These results were merged with species‐specific genome‐wide annotation for zebrafish provided in the package org. Dr.eg.db version 3.11.4 (Carlson, 2019), using Entrez stable gene identifiers (Maglott et al., 2010) and GenBank accessions to annotate BLASTX alignments of gene locus models. Common Gene Locus (gene model g1… g28416) from blast reports were also used to link transcripts to zebrafish and yellowtail kingfish accession and description information. The Bioconductor (Huber et al., 2015) packages GO.db and org. Dr.eg.db (Carlson, 2019) provided annotation maps describing the entire Gene Ontology (http://geneontology.org).

### 2.4 Sequencing data processing

The quality of the RNA‐seq raw sequences from pituitary and liver tissues was checked using FastQC version 0.11.7 (Andrews, 2017) and MultiQC version 0.11.7 (Ewels et al., 2016). Reads were aligned to the trevally reference genome using the splice site aware aligner STAR version 2.6.1 (Dobin et al., 2013), with the parameter ‐‐clip5pNbases (clipping 12 bases from the 5′ end of each read) the aligned reads were indexed and sorted using Samtools version 1.7 (Li et al., 2009). The number of reads aligning to each transcript for each sample was counted using HTSeq version 0.9.1 (Anders et al., 2015). Libraries were validated to belong to the correct tissue type by aligning them to NCBI downloaded sequences of genes unique to pituitary (Fsh‐β and Lh) or liver (hemopexin b and apolipoprotein A‐IV‐like) using the Burrows‐Wheeler Aligner (BWA) version 0.7.17, with the bwa‐mem option.

### 2.5 Differential expression analysis

Filtering, normalization and analysis were done using the Bioconductor package edgeR version 3.14.0 (Robinson et al., 2010). Duplicated genes and those with unknown annotation information were filtered out prior to normalization. After graphically comparing the trimmed mean of M‐values (TMM; Robinson & Oshlack, 2010) with the TMM with singleton pairing (TMMwsp) normalization methods (Figure S1), the normalization factors were estimated using TMM to adjust for different library sizes. This step did not require correction for individual gene lengths as QuantSeq library preparations preferentially target the 3′ end of individual transcripts. Genewise dispersions were estimated by conditional maximum likelihood, conditioning on the total count for each gene (Smyth & Verbyla, 1996) utilizing information between genes (Robinson & Smyth, 2007). Within a tissue, differential expression between temperature treatments was assessed for each gene using a negative binomial generalized linear model with the function ‘glmTREAT’ (McCarthy et al., 2012), which contains an in‐built test for differential expression relative to a minimum required fold‐change threshold. A gene was considered to be differentially expressed (DEG) between both treatments when the |log_2_FC| > 0.26 (e.g. 1.2 difference between treatments) and FDR‐adjusted *p*‐value < 0.05 (Benjamini & Hochberg, 1995).

### 2.6 Gene ontology

Gene Ontology (GO) analysis tested for over‐representation of GO terms using the edgeR function goana (Young et al., 2010). The enrichment was run on the DEGs with the parameter species = ‘Dr’ mapping to the zebrafish GO terms. GO terms with *p*‐value < 0.01 were considered significant and were visualized with the online tool REViGO (Supek et al., 2011). The same set of genes was also input into the Kyoto Encyclopedia of Genes and Genomes (KEGG; Kanehisa & Goto, 2000) using the edgeR function kegga, with the same parameters as mentioned above.

## 3 RESULTS

### 3.1 Weight increased in the high temperature treatment by 50%

The average weights at the beginning of the experiment were 38.34 ± 7.73 and 37.31 ± 7.62 g in the high and low treatment, respectively. At the end of the experiment, average weights were 62.28 ± 13.09 g in the high and 41.74 ± 8.15 g in the low treatments. While no significant differences were observed between the fish at the beginning of the experiment (*t* test, *p*‐value = 0.1813), growth was significantly different upon termination (*t* test, *p*‐value < 2.2^−16^), where fish increased 50% more in weight in the warmer compared with the colder condition (Figure 1b).

### 3.2 Raw sequencing data and quality statistics

The three lanes of Illumina NovaSeq 6000 produced close to 1.22 billion single‐end raw reads (Table S1). On average, 60% of the reads were successfully aligned, leaving over 772.5 million reads for downstream analysis. The final number of reads per individual ranged from 1.33 to 9.19 million (mean = 3.86 million ± 1.78 million). The number of reads aligned in each treatment group was balanced with 403.3 million in the high group (20°C) and 369.2 million in the low group (13°C).

### 3.3 Functional annotation and filtering

A total of 28,416 gene models were predicted using BRAKER. A summary of the number of transcripts kept after each filtering steps is given in (Table 1). 27,887 meaningful unigenes (98.14% of the total gene models) were annotated against the genome‐wide annotation for zebrafish databases using BLASTX. The exploratory plot (Figure S2) illustrated that longer matches (with fewer mismatches) had smaller *E*‐values, indicating that when *E*‐value < 0.01, *p*‐values and *E*‐value are nearly identical; hence, no threshold for the *E*‐value cut‐off was used so that all annotation matches were included. Gene models seem to split in a mass of two matches, one of small length and another one of larger length. 17,980 gene models were left after filtering for duplicated symbols and 8597 genes from the expression set mapped to the filtered gene models after filtering for a minimum count of 0 (Figure S3).

**TABLE 1:** Summary of filtering steps of the transcripts

Step	Genes	
	Keep	Exclude
Before filtering	28,416	
GenBank accession known	27,887	529
Unique gene symbol	17,980	9907
Filter by expression	8597	9383
After filtering	8597	

### 3.4 Differential gene expression analysis revealed extensive transcriptional plasticity

**FIGURE 2:**
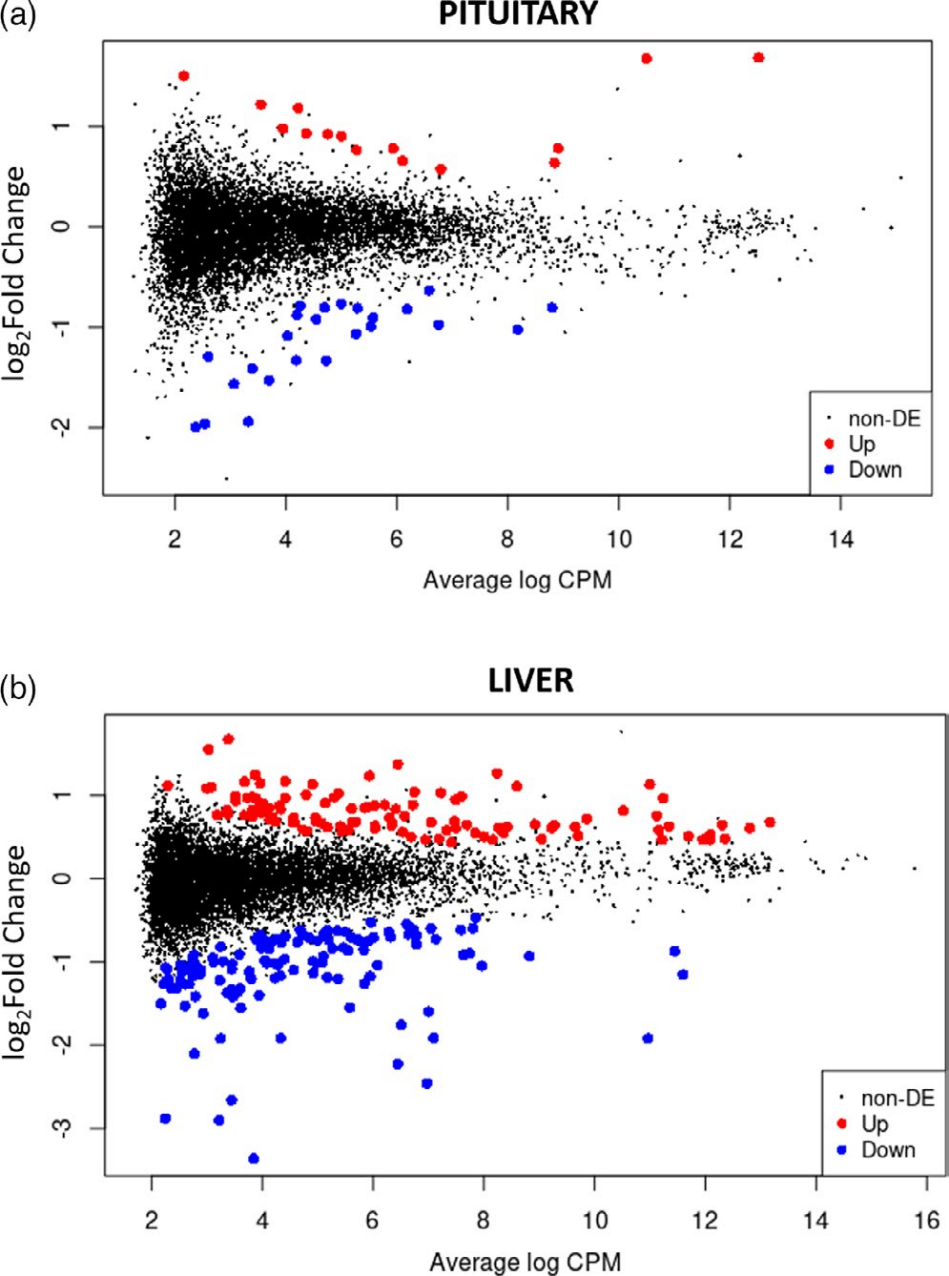
Differential expression of genes (DEG) between high and low treatment in the pituitary (a) and the liver (b). The MA plots show the average log of Count per Million (CPM; *X*‐axis) and log2 Fold Changes (*Y*‐axis). DEGs up‐ and down‐regulated in the high versus low treatment are coloured in red and blue, respectively (*p*‐adjusted < 0.05), |log2FC| > 0.26. Those shown in black were unigenes that did not show significant expression

Differential gene expression patterns in pituitary and liver in response to temperature were determined after 32 days. Multidimensional scaling of the samples showed that most of the variation between the samples was explained by the tissue type (Figure S4a), rather than the treatment (Figure S4b; note: the clustering was made using the 2000 first genes models of the 8597 gene model list). Normalization of the data showed that the pituitary samples generally needed higher size factors than the liver samples (Figure S5a). Overall, the normalization factors were normally distributed (Figure S5b) and more biological variation was observed within the pituitary samples (Figure S6a) compared with the liver samples (Figure S6b). 39 DEGs were detected in the pituitary, accounting for 0.46% of the gene models (Figure 2a, Table S2), and 238 DEGs were found in the liver, accounting for 2.72% of the transcriptome (Figure 2b, Table S3).

**FIGURE 3:**
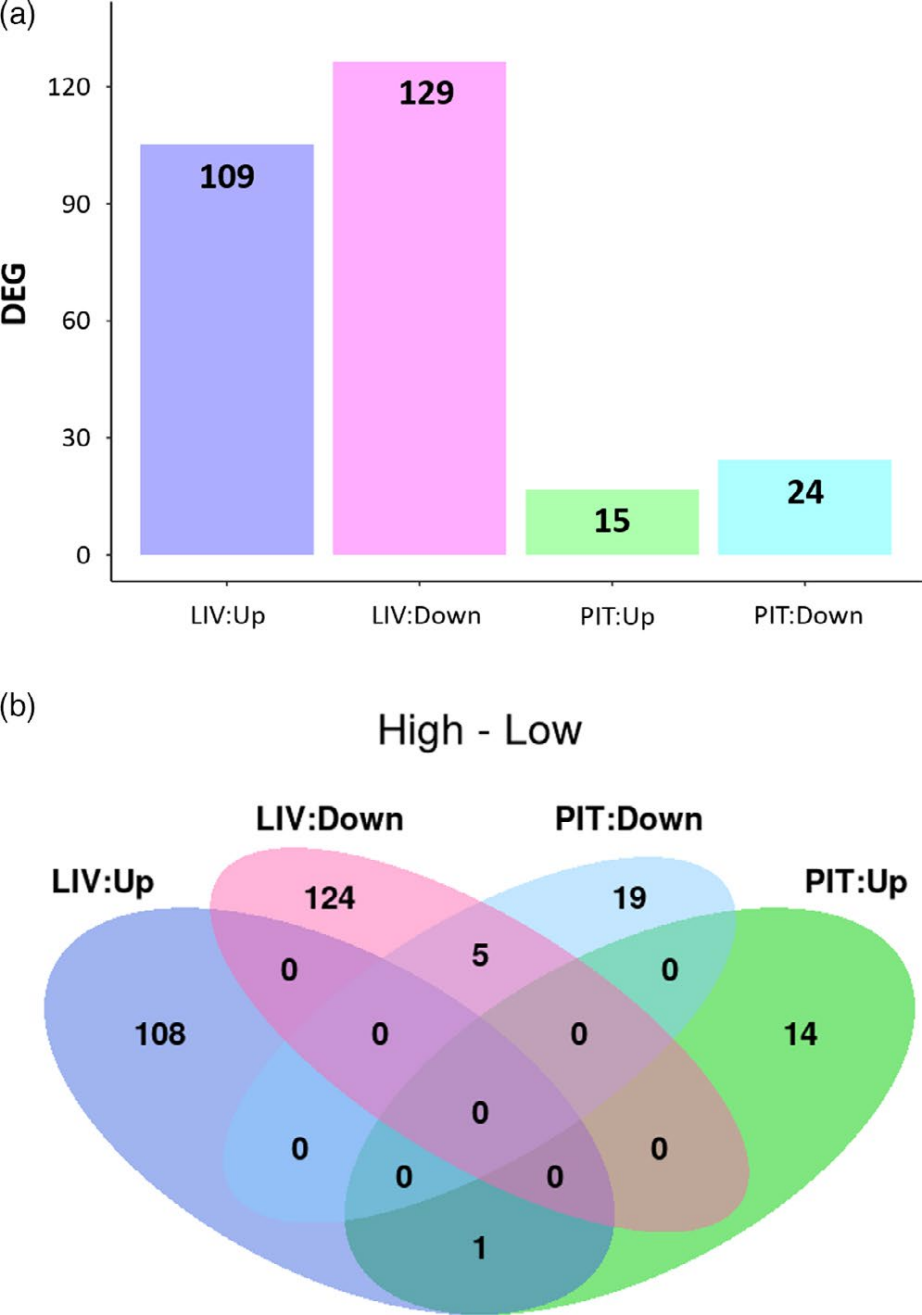
Differential gene expression (DEG) within the tissues. (a) Bar plot of the number of DEGs in each tissue between high and low temperature treatment. (b) Venn diagram showing the number of DEGs up‐ and down‐regulated in the pituitary (PIT) and in the liver (LIV). There were only six DEGs shared between pituitary and liver, five of which were down‐regulated and one up‐regulated

In the pituitary, 15 and 24 DEGs were up‐ and down‐regulated respectively, in the high versus low treatment. In the liver, 109 DEGs were up‐regulated and 129 DEGs were down‐regulated (Figure 3a). In both tissues, many of these DEGs corresponded to genes usually considered as ‘housekeeping genes’, that is expressed in most cells and involved in the maintenance of basal cellular functions. These included for instance genes coding for RNA splicing proteins, translation factors, or enzymes involved in metabolism. The growth hormone receptor gene Ghrb was found to be up‐regulated in the liver. Interestingly, genes involved in the production of the sexual hormones Fsh and Lh were up‐regulated in the high treatment in the pituitary. In the liver, two genes coding for zona pellucida proteins (the acellular membrane surrounding the egg) were found to be down‐regulated. Comparison of gene expression between treatments and tissue types identified five common down‐regulated transcripts in pituitary and liver (Figure 3b), which were tet2 (promoting DNA demethylation); iscub (which codes for the scaffold protein involved in the de novo synthesis of iron‐sulphur clusters within mitochondria); thyn1 (involved in the induction of apoptosis); butyrophilin‐like protein 2 gene (associated with the immune system and the major histocompatibility complex); and gstr (involved in detoxification processes). One gene was found up‐regulated in both tissues, coding for a peptidylprolyl isomerase, which is involved the cellular response to oestrogen stimulus.

### 3.5 Gene ontology to reveal molecular pathways

**FIGURE 4:**
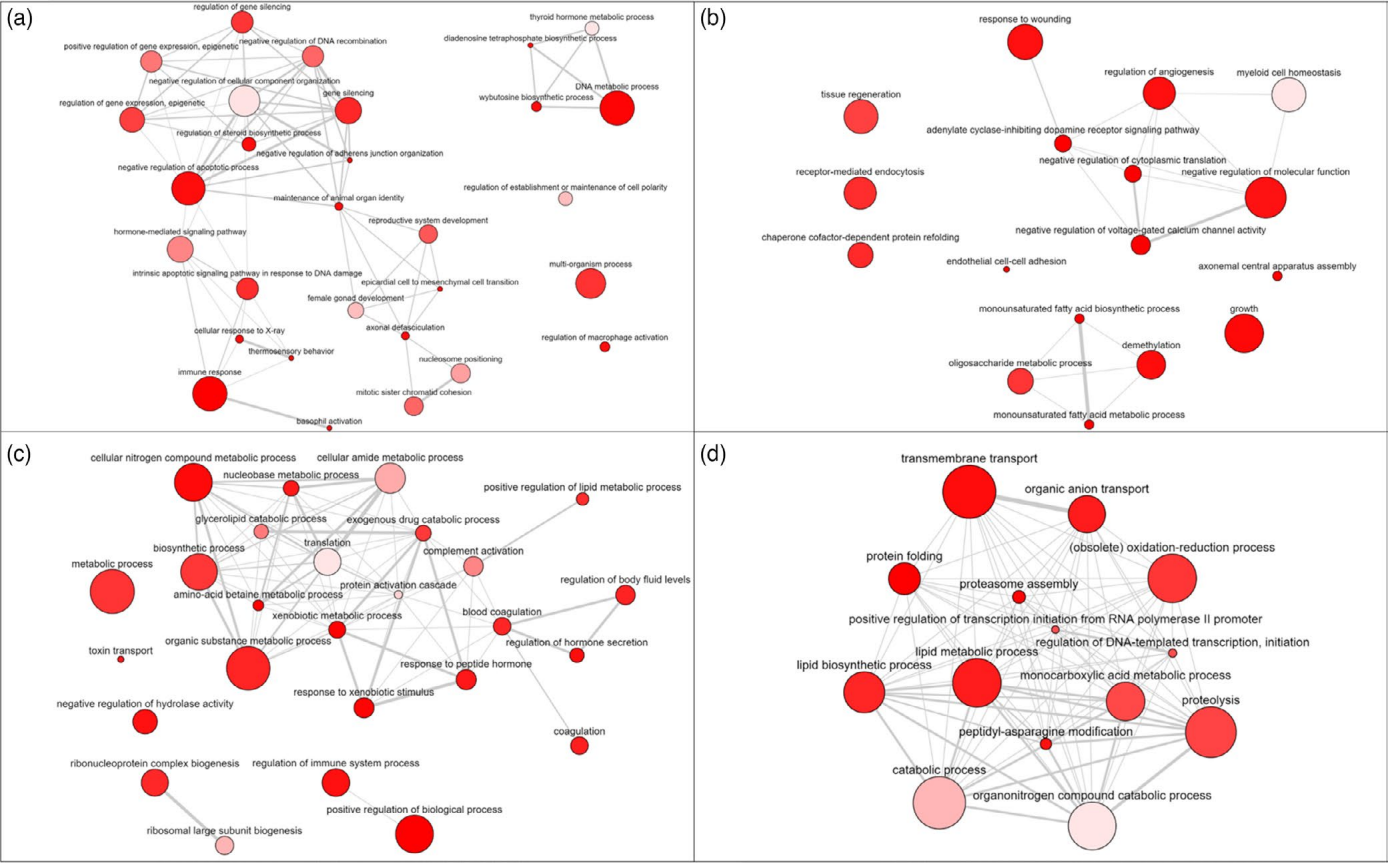
Network plots of Gene Ontology (GO) enriched terms for biological processes which were up (a) down (b) regulated in the pituitary and up (c) or down (d) regulated in the liver. Bubble colour indicates the user‐provided *p*‐value; bubble size indicates the frequency of the GO term in the underlying GOA database. Highly similar GO terms are linked by edges in the graph, where the line width indicates the degree of similarity. The initial placement of the nodes is determined by a ‘force‐directed’ layout algorithm that aims to keep the more similar nodes closer together

The most significantly enriched GO term in each tissue depended on the temperature treatment: in the pituitary, genes involved in the female gonad development (GO:0008585), thyroid hormone metabolic process (GO:0042403) and the negative regulation of cellular component organization (GO:0051129) were up‐regulated (Figure 4a and Figure S7a), whereas genes involved in myeloid cell homeostasis (GO:002262) were down‐regulated (Figure 4b and Figure S7b, Table S4). In the liver, genes involved in translation (GO:0006412), protein activation cascade (GO:0072376) and cellular amide metabolic processes (GO:0043603) were up‐regulated (Figure 4c and Figure S7c), and genes involved in catabolic processes (GO:0009056) were down‐regulated (Figure 4d, Figure S7d, Table S5).

The distribution of DEGs among KEGG terms further confirmed that in the pituitary, the most enriched up‐regulated term was Gnrh‐signalling pathway (ko4912) and down‐regulated was lysosome (ko04142; Table S6). In the liver, terms that were part of the ribosome (ko03010) were up‐regulated, and terms that were part of the proteasome (ko03050) were down‐regulated (Table S7).

## 4 DISCUSSION

Plastic responses to temperature are essential among ectothermic organisms, as all aspects of their physiology are directly dependent on their thermal environment. Therefore, understanding how thermal variation modulates phenotypic plasticity and its effect on major molecular pathways involved in the endocrine regulation of somatic growth is important. In the present study, transcriptomes of pituitary and liver tissues from 100 trevally were sequenced to investigate their physiological regulation mechanisms under different temperatures. Two hundred RNA‐seq libraries were constructed and sequenced, and 28,416 transcripts (N50 = 1980 bp) were identified after assembly. A total of 17,980 genes with unique gene symbols were annotated successfully, which accounted for about 63.3% of all predicted gene models. To the best of our knowledge, this study represents the largest investigation of the thermal phenotypic plasticity of any carangid to date in terms of number of samples, and one of the largest for any teleost species. Our results show that somatic growth of trevally increased by 50% more in the high (20°C) compared with the low (13°C) treatment. These findings are in general agreement with other teleost studies, and the realized growth gain in the warm treatment is very encouraging and provides important knowledge for selecting suitable grow‐out locations for this species around New Zealand so it can thrive. In the Carangid family specifically, optimal metabolic temperature ranges range from 20 to 25°C for yellowtail kingfish (Brown et al., 2011; Pirozzi & Booth, 2009) and 22–26°C in greater amberjack fingerlings (*Seriola dumerili*; Fernández‐Montero et al., 2018, 2020). Differences in growth rate show that temperature has a pronounced effect on the growth phenotype of trevally, which is in line with the seasonal influence on growth described for this species (Valenza‐Troubat et al., 2021), an all of this information provides crucial insights into the bio‐economic feasibility of this species as a new aquaculture species for New Zealand.

We found that 39 and 238 genes were differentially regulated in the high versus low treatments in the pituitary and liver, respectively, demonstrating that temperature has a pronounced effect on gene expression. Many housekeeping genes (Watson, 1970), usually ubiquitously expressed across tissue type and developmental or cell cycle stage and required for the maintenance of functions essential for a cell's existence, were among the DEGs. Differential expression of housekeeping genes has been linked to stress (Torres‐Contreras et al., 2018; Wang et al., 2013) and used as a marker for diseases (Byun et al., 2009). Important genes and pathways linked to the somatotropic and reproductive axes were also highlighted. The growth hormone receptor gene (*Ghrb*), which is the transmembrane receptor for growth hormone, was found to be up‐regulated in the liver. This class of receptor is a critical regulator of growth and metabolism in vertebrates; however, growth hormone, its ligand, was not found to be significantly differentially expressed in the pituitary. Interestingly, the term growth (GO:0040007) was enriched in the pituitary in the low compared with the high treatment. Several hypotheses could explain this result: first, this pathway may need to be inactivated in order for growth to happen; second, at the time of the termination of the experiment, the fishes in the low treatment might have been overexpressing genes involved in the growth pathway to counter‐balance their slow metabolism, or third, the control of growth has been rewired in response to the two temperature regimes. In case of the latter, the increased density of the receptor in the liver tissue may provide a controlled way to modulate growth without overexpressing GH and suffering trade‐offs due to pleiotropic effects, without increasing the amount of ligand. Our results also suggest that the expression of genes associated with female gonad development was increased in the pituitary in the high temperature treatment. Fsh and Lh are gonadotropin hormones synthesized in the pituitary and modulate gonadal steroids (Nett et al., 2002). Zona pellucida proteins (ZPs) were also found to be down‐regulated in the liver. Studies have found ZPs to be lowly expressed in the liver of sexually mature fish like in the gilthead seabream (*Sparus aurata*; Modig et al., 2006). This result highlights that either high temperatures onset early sexual maturation of immature juvenile fish or that cold temperature delayed reproductive development in trevally. To date, little is known about reproduction in this species, particularly in captive aquaculture stocks of trevally. Thus, to explore this further, detailed surveys about sexual maturation and the reproductive cycle and their relationship to factors such as temperature and sex, as well as to growth, need to be carried out to quantify trade‐offs. Overall, warmer temperature appeared to stimulate protein synthesis, cellular differentiation and lipid metabolism, as well as regulate transcription of reproductive hormones. These processes are all important for myogenesis, somatic growth and sexual maturation in teleost. Colder temperatures had a positive effect on the expression of genes involved in blood cell differentiation and tissue injury, inflammation, and immunity (lysosome).

To the best of our knowledge, very few studies have simultaneously investigated the gene expression of both pituitary and liver, two of the main organs involved in the endocrine regulation of somatic growth. Two studies used comparative transcriptome analysis between pituitary and liver in teleost, of which only one investigated the effects of temperature on these two vital organs. Fu et al. (2019) looked at hypothalamo‐pituitary (HP) and liver transcriptomes of bighead carp (*Hypophthalmichthys nobilis*) with high or low body weight and found genes enriched for the GnRH signalling pathway in the HP organs, which the KEGG analysis in our study also highlighted. GnRH is part of the reproductive feedback loop, where it is responsible for the release of Fsh and Lh by the pituitary. It indicated that fish in the high treatment may have begun to mature sexually, which is a proxy for general sufficient growth as well as fitness. This implies that the juvenile immature fish grew to a sufficient stage to begin the process of sexual maturation in the warmer treatment. A study on golden pompano (*Trachinotus ovatus*), a species from the same family as trevally, also compared the effects of different temperature treatments on pituitary and liver gene expression (Zhou et al., 2020). Interestingly, they found more genes to be differentially expressed in the pituitary than in the liver (458 and 205, respectively). Our study showed the inverse relationship, with only 39 DEGs in the pituitary and 238 DEGs in the liver, showing that metabolic processes were by far largest in the liver, which we would expect given the important involvement of this tissue in the regulation of growth, as well as in other physiological functions. It is to be noted that the low treatment in the pompano study corresponded to the high treatment of this study (20°C), which could explain some of the differences observed. Similar to their results, we found enriched terms linked to transporter activity, enzyme activity, and transcription factor activity in the pituitary and immune regulation, digestion, and protein metabolism pathways in the liver.

Another interesting comparison to make is with other teleost species that share a similar geographic distribution to trevally. Using a similar set‐up, Wellenreuther et al. (2019) investigated the effects of cold/hot temperature treatments on the gene expression of the Australasian snapper, another native species of New Zealand, also considered a candidate for aquaculture. They identified that temperature had a major effect on gene expression in both domesticated and wild strains of snapper, highlighting changes in protein synthesis and cellular multiplication. Our study also found enriched terms for ribosomal activity (GO:0005840) in the liver, and nucleosome (GO:0000786) and mitotic sister chromatid cohesion (GO:0007064) in the pituitary, which are involved in cell division. Interestingly, differences in growth between domesticated and wild snapper were found to occur through an interaction of the immune response and anabolic growth pathway modulation, highlighting that in the wild F_1_ strain, immune‐related activity was being prioritized over growth‐related functions. Immune system responses were also found to be influenced by temperature in trevally; however, our findings show enrichment in these pathways in the high compared to the low treatment. This could demonstrate that despite geographical similarities, these two species have differences in thermal performance curves. Interspecific adaptive differences can be caused by gene expression plasticity but mechanisms, including, epigenetic effects, developmental plasticity and neutral genetic variation should not be excluded (Schulte et al., 2011), but these would all require future work.

### 4.1 Management implications and future directions

In marine organisms, temperature is one of the most important environmental factors and affects the majority of physiological activities, such as metabolism, growth, and development (Brierley & Kingsford, 2009; Seibel & Drazen, 2007). In New Zealand, aquaculture currently relies on only three species, namely Greenshell™ mussel (*Perna canaliculus*), Chinook salmon (*Oncorhynchus tshawytscha*), and Pacific oyster (*Crassostrea gigas*). Only one of these, Chinook salmon, is a finfish species, and because of the temperature constraints of this species, it can only be grown around the cool waters of the South Island of New Zealand. There is thus an urgent need to diversify the species that can be grown around New Zealand more generally, and also more specifically, to develop species that can be farmed around the North Island. Selecting the right grow‐out locations for new species is largely dependent on the temperature profiles, as they determine survival and growth, and so this study adds important knowledge about potential regions where trevally can thrive given the temperatures experienced. Our study shows that key responses to temperature depend on the activation of complex molecular networks involved in metabolic, immune and stress signal transduction pathways. Our workflow demonstrates the utility of RNA‐seq analysis for transcriptome profiling and shows that it can be used as a powerful strategy to assess a species’ responses to environmental fluctuations. Given that the Intergovernmental Panel on Climate Change (IPCC) projects a global increase in water temperature, under all emission scenarios, of 1.5–3°C by the end of this century (IPCC et al., 2018), the physiological constrains imposed on fishes are likely to cause significant challenge for aquaculture programmes worldwide. Climate change has already started influencing the intensity and frequency of extreme weather events globally (Pachauri et al., 2014), so predicting the fish's responses to ambient water fluctuations is becoming an increasing concern for sea‐pen farming. The economic impact for aquaculture could be profound if plasticity mechanisms are not well understood. Uncontrolled phenotypic plasticity mechanisms have been shown to influence the genetic gain calculation in many livestock breeding programmes (Nguyen et al., 2017) and are an important parameter to assess for newly domesticated species (Zhang et al., 2016). Temperature fluctuations induce shifts in a variety of phenotypic traits, mostly related to reproduction and life history. Onset of early sexual maturation will be of prime importance for breeding programmes as it reduces growth rates due to the reallocation of energetic resources to reproductive organs (Gjerde et al., 1994; Oppedal et al., 1997). Moreover, organisms that are able to quickly adjust their physiology to environmental changes, that is exhibiting high phenotypic plasticity, may simply be better equipped to survive thermal fluctuations (Fu et al., 2016; Sandblom et al., 2014). Although all fish species exhibit unimodal thermal performance curves, each has its own specific parameters (i.e. thermal tolerance range, optimal growth temperature), and regulatory mechanisms can therefore vary. Selection for faster growth can offset the potential losses from climate change (Janssen et al., 2017). However, the genetic variability that differentiates plastic responses among families should also be harnessed to mitigate the effects of temperature through selection for shifted optimal temperature for growth (Sae‐Lim et al., 2016). Adaptive potential found through standing genetic and epigenetic variation, and newly derived mutations will be a way for selective breeding programmes to produce phenotypes that can compensate for growth losses in suboptimal temperatures.

## CONFLICT OF INTEREST

The authors declare that they have no conflict of interest.

## Supporting information

Supplementary Material

## Data Availability

As the genomic data of this species are from a taonga and thus culturally important species in Aotearoa New Zealand, the data (genome assembly, RNA‐seq libraries) have been deposited in a managed repository that controls access. Data are available through the Genomics Aotearoa data repository at https://repo.data.nesi.org.nz/. This was done to recognize Māori as important partners in science and innovation and as intergenerational guardians of significant natural resources and indigenous knowledge.
