# Supplementary material for: Differential expression analyses reveal extensive transcriptional plasticity induced by temperature in New Zealand silver trevally (Pseudocaranx georgianus)
Source: Evol Appl. 2022 Jan 22;15(2):237–48. doi: 10.1111/eva.13332 (PMC8867707; doi:10.1111/eva.13332)
Supplement: Supplementary file 1 — Supplementary Material [file EVA-15-237-s001.docx]

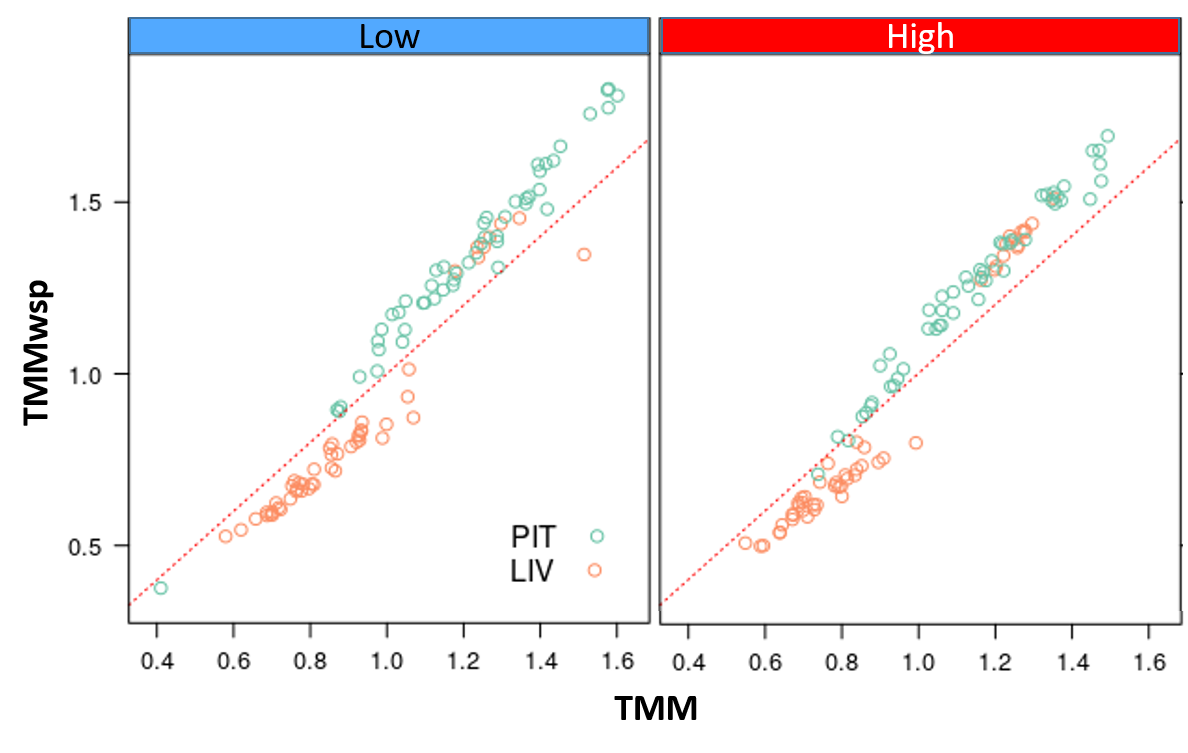


Supplementary Figure 1: Comparison of the normalisation factors calculated by trimmed mean of M-values (TMM) and TMM with singleton pairing (TMMwsp) methods for pituitary (PIT) and liver (LIV) samples in the high and low treatments.


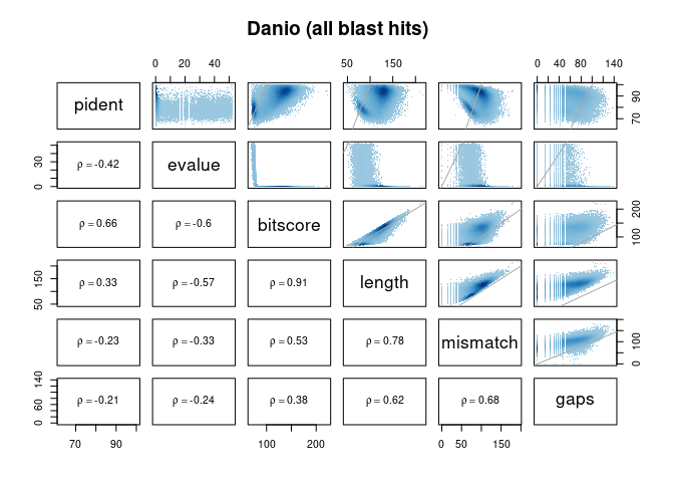


Supplementary Figure 2: Pairwise exploratory plot of the statistical metrics from the *Danio rerio* BLASTX report. This plot shows the degree of correlation between different BLASTX metrics. pident is percentage identity, evalue is the expect value, bitscore is the statistical metric defining similarity (related to the number of identity matches between two sets of sequences).


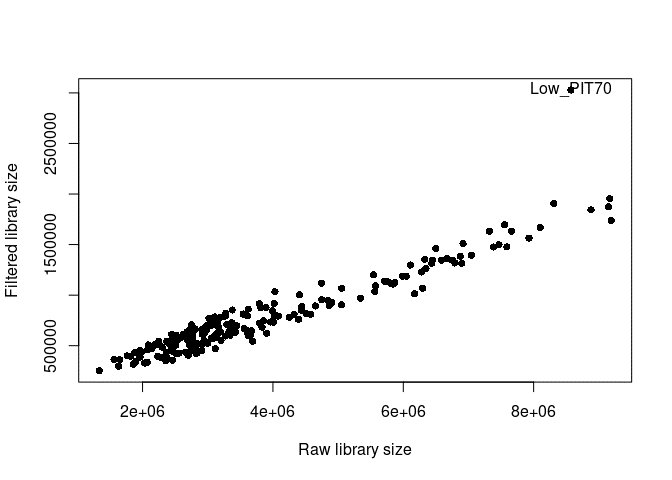


Supplementary Figure 3: Changes in library sizes after filtering. This plot shows the raw library sizes (x-axis) compared with the library sizes after filtering for genes that have sufficiently large counts to be retained in a statistical analysis.


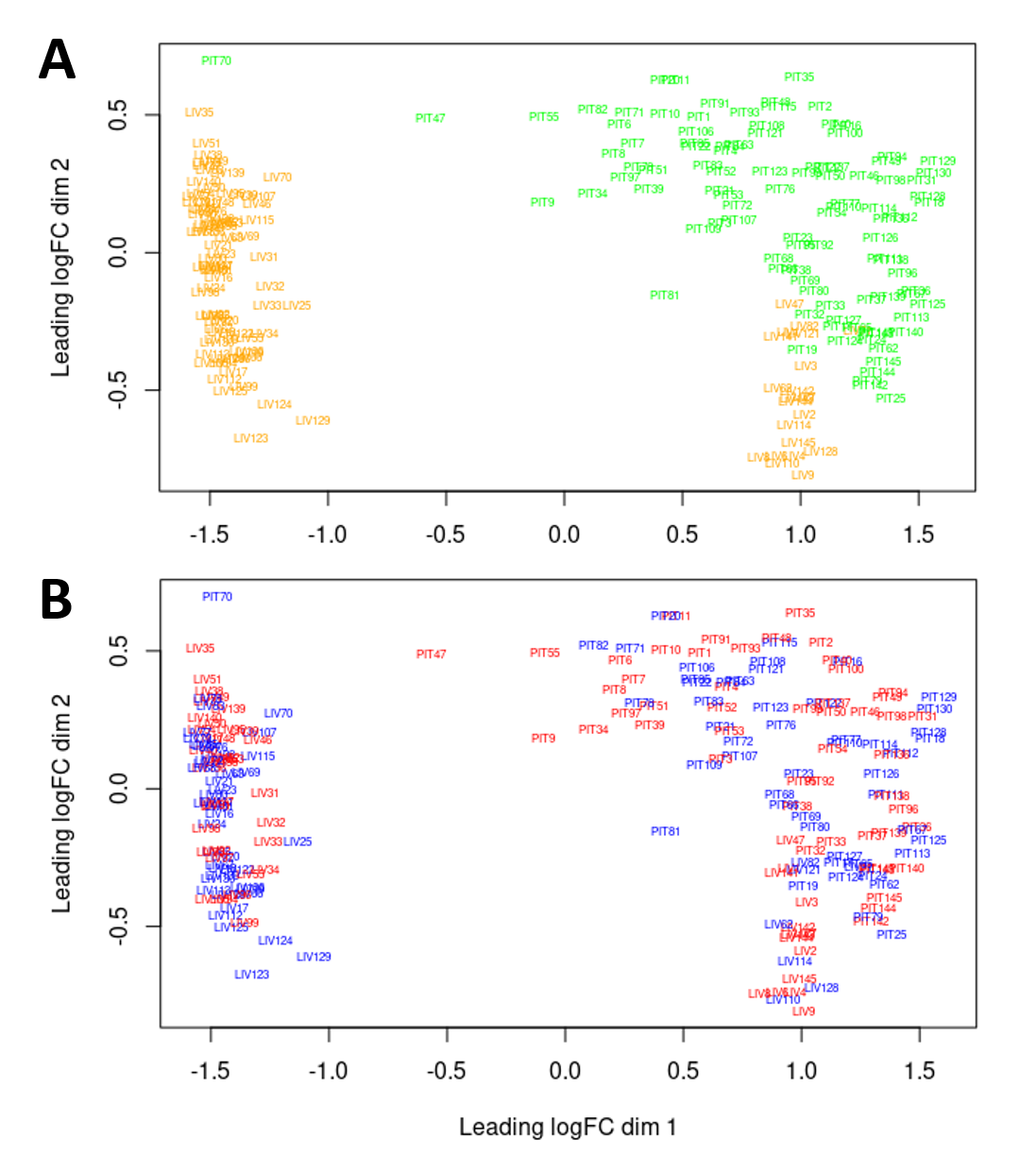


Supplementary Figure 4: Multidimensional scaling plots comparing the 2000 first genes of the gene model list between (a) pituitary (orange) and liver (green) or (b) between high (red) and low (blue) treatments.


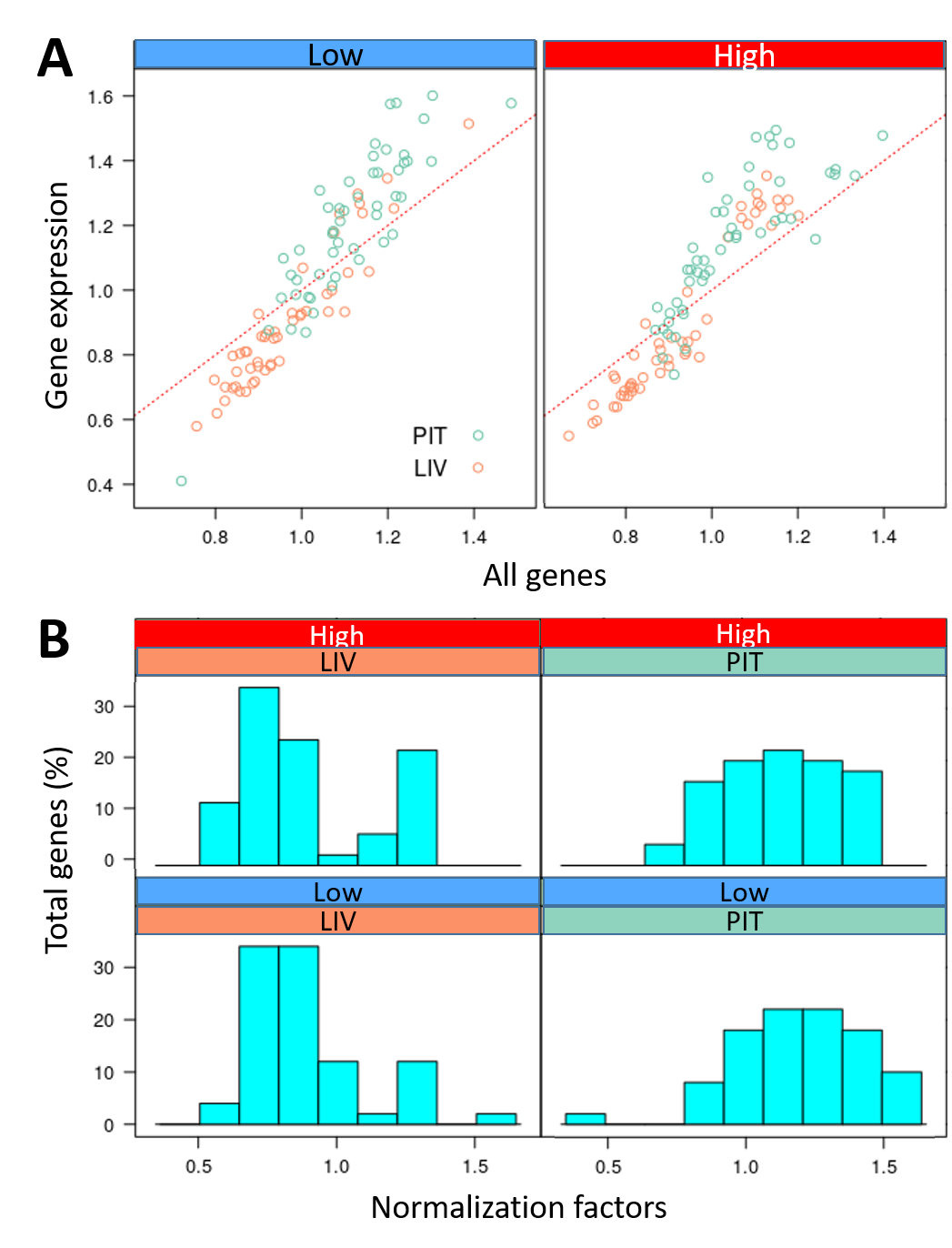


Supplementary Figure 5: Exploratory plots after normalisation using the TMM method. (a) Normalisation factors for pituitary (PIT) and liver (LIV) samples, (b) Distribution of the normalisation factors in function of tissue and treatment.


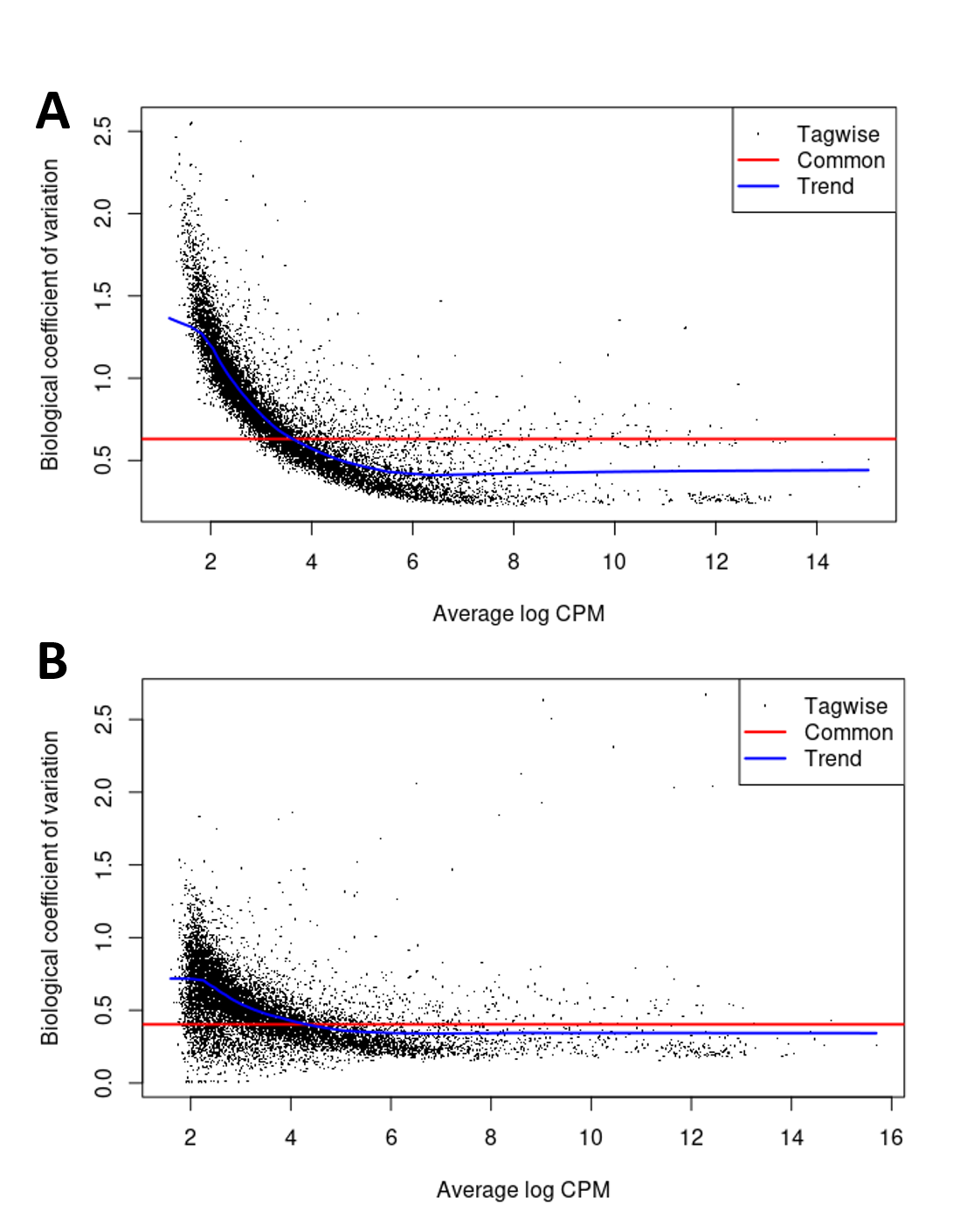


Supplementary Figure 6: Dispersion estimates in the pituitary (a) and in the liver (b) samples. Genewise biological coefficients of variation are plotted against gene abundance. CPM: Count per Million.


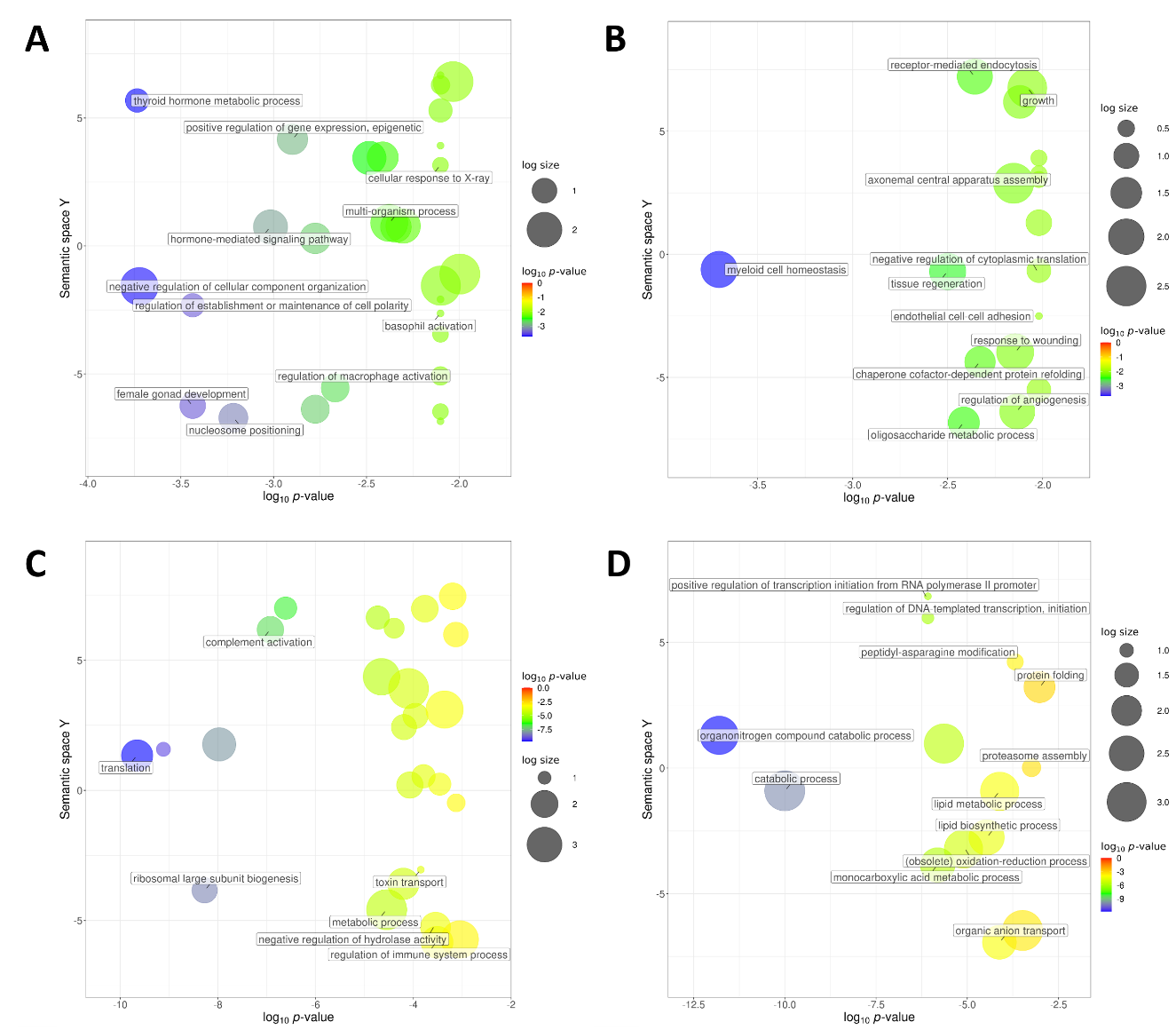


Supplementary Figure 7: Enrichment of Gene Ontology (GO) terms associated with the genes that were differentially expressed in the pituitary gland and in the liver between the two temperature treatments. GO-terms enrichment for genes up- or down-regulated in the pituitary (a and b, respectively) and in the liver(c and d, respectively). For each GO-term, the colour saturation indicates the log_10_ Fisher’s *p*-value of enrichment from smaller (blue) to higher (red) along the x-axis and the semantic space y after multidimensional scaling of GO-terms semantic similarities performed by REViGO in the y-axis. The log size indicates the number of GO-terms represented.

Supplementary Table 1: Summary mapping samples to Next-Generation Sequencing files and numbers of raw and aligned reads.

| Temperature | Tissue | Fish_ID | Prefix | Raw reads | Aligned reads |
| --- | --- | --- | --- | --- | --- |
| High | LIV | 1 | LIV1_HLJ7VDRXX_TTGGTA-TAGGGC_L002 | 8437401 | 5576427 |
| High | LIV | 2 | LIV2_HLJ7VDRXX_GTTACC-TAGGGC_L002 | 15001477 | 9189667 |
| High | LIV | 3 | LIV3_HLJ7VDRXX_CGCAAC-TAGGGC_L002 | 7137781 | 4652641 |
| High | LIV | 4 | LIV4_HLJ7VDRXX_TGGCGA-TAGGGC_L002 | 9106321 | 5344004 |
| High | LIV | 6 | LIV6_HLJ7VDRXX_ACCGTG-TAGGGC_L002 | 7331929 | 4433695 |
| High | LIV | 7 | LIV7_HLJ7VDRXX_CAACAG-TAGGGC_L002 | 7515064 | 4871432 |
| High | LIV | 8 | LIV8_HLJ7VDRXX_GATTGT-TAGGGC_L002 | 7166218 | 4251776 |
| High | LIV | 9 | LIV9_HLJ7VDRXX_ATGGCG-TAGGGC_L002 | 7763032 | 4578677 |
| High | LIV | 10 | LIV10_HJK3MDRXX_TCAGGA-CGCCAT_L001 | 3974885 | 2272254 |
| High | LIV | 11 | LIV11_HJK3MDRXX_CGGTTA-CGCCAT_L001 | 5605095 | 3793449 |
| High | LIV | 31 | LIV31_HJK3MDRXX_GAGTCC-CGCCAT_L001 | 4909647 | 2683163 |
| High | LIV | 32 | LIV32_HJK3MDRXX_GGAGGT-CGCCAT_L001 | 4960173 | 2163308 |
| High | LIV | 33 | LIV33_HJK3MDRXX_CACACT-CGCCAT_L001 | 5406318 | 2396143 |
| High | LIV | 34 | LIV34_HJK3MDRXX_CCGCAA-CGCCAT_L001 | 5152834 | 2673470 |
| High | LIV | 35 | LIV35_HJK3MDRXX_TTTATG-CGCCAT_L001 | 4174840 | 3018963 |
| High | LIV | 36 | LIV36_HJK3MDRXX_AACGCC-CGCCAT_L001 | 4864172 | 2574904 |
| High | LIV | 37 | LIV37_HJK3MDRXX_CAAGCA-CGCCAT_L001 | 4843682 | 2629572 |
| High | LIV | 38 | LIV38_HJK3MDRXX_GCTCGA-CGCCAT_L001 | 4945868 | 3190366 |
| High | LIV | 39 | LIV39_HJK3MDRXX_GCGAAT-CGCCAT_L001 | 4952246 | 2923579 |
| High | LIV | 40 | LIV40_HJK3MDRXX_TGGATT-CGCCAT_L001 | 5848518 | 2984547 |
| High | LIV | 46 | LIV46_HJK3MDRXX_ACCTAC-CGCCAT_L001 | 4987724 | 3041861 |
| High | LIV | 47 | LIV47_HLJ7VDRXX_ATTGGT-TAGGGC_L002 | 9465255 | 6670320 |
| High | LIV | 48 | LIV48_HJK3MDRXX_AGATAG-CGCCAT_L001 | 3489651 | 2089499 |
| High | LIV | 49 | LIV49_HJK3MDRXX_TTGGTA-CGCCAT_L001 | 4571797 | 2514551 |
| High | LIV | 50 | LIV50_HJK3MDRXX_GTTACC-CGCCAT_L001 | 4422446 | 2374783 |
| High | LIV | 51 | LIV51_HJK3MDRXX_CGCAAC-CGCCAT_L001 | 4317092 | 2444950 |
| High | LIV | 52 | LIV52_HJK3MDRXX_TGGCGA-CGCCAT_L001 | 3958906 | 1877836 |
| High | LIV | 53 | LIV53_HJK3MDRXX_ACCGTG-CGCCAT_L001 | 4235397 | 1961790 |
| High | LIV | 54 | LIV54_HJK3MDRXX_CAACAG-CGCCAT_L001 | 5101179 | 2689711 |
| High | LIV | 55 | R-LIV55_HK5YKDRXX_CCTAAG-GCAACG_L001 | 5151796 | 2511818 |
| High | LIV | 91 | LIV91_HJK3MDRXX_GAAGTG-CGCCAT_L001 | 4764930 | 2917972 |
| High | LIV | 92 | LIV92_HJK3MDRXX_CAATGC-CGCCAT_L001 | 4947005 | 2442157 |
| High | LIV | 93 | LIV93_HJK3MDRXX_ACGTCT-CGCCAT_L001 | 4962543 | 2682900 |
| High | LIV | 94 | LIV94_HJK3MDRXX_CAGGAC-CGCCAT_L001 | 5725461 | 2738814 |
| High | LIV | 95 | LIV95_HJK3MDRXX_AAGCTC-CGCCAT_L001 | 4133716 | 2454381 |
| High | LIV | 96 | LIV96_HJK3MDRXX_GACGAT-CGCCAT_L001 | 6073796 | 3145864 |
| High | LIV | 97 | LIV97_HJK3MDRXX_TCGTTC-CGCCAT_L001 | 4629739 | 2304375 |
| High | LIV | 98 | LIV98_HJK3MDRXX_CCAATT-CGCCAT_L001 | 5545528 | 2951165 |
| High | LIV | 99 | LIV99_HJK3MDRXX_AGTTGA-CGCCAT_L001 | 5191835 | 2752175 |
| High | LIV | 100 | LIV100_HJK3MDRXX_AACCGA-CGCCAT_L001 | 4577712 | 1986026 |
| High | LIV | 136 | LIV136_HJK3MDRXX_GCGCTG-CGCCAT_L001 | 6943494 | 3893053 |
| High | LIV | 137 | LIV137_HJK3MDRXX_GAACCT-CGCCAT_L001 | 6719869 | 4020331 |
| High | LIV | 138 | LIV138_HJK3MDRXX_TTCGAG-CGCCAT_L001 | 6551743 | 3814532 |
| High | LIV | 139 | LIV139_HJK3MDRXX_AGAATC-CGCCAT_L001 | 5645071 | 3542549 |
| High | LIV | 140 | LIV140_HJK3MDRXX_AGGCAT-CGCCAT_L001 | 7020815 | 4744813 |
| High | LIV | 141 | LIV141_HLJ7VDRXX_CTCATA-TAGGGC_L002 | 9392167 | 6873618 |
| High | LIV | 142 | LIV142_HLJ7VDRXX_CCGACC-TAGGGC_L002 | 8469523 | 5870859 |
| High | LIV | 143 | LIV143_HLJ7VDRXX_GGCCAA-TAGGGC_L002 | 9611133 | 6790572 |
| High | LIV | 144 | LIV144_HLJ7VDRXX_AGACCA-TAGGGC_L002 | 10974812 | 7588885 |
| High | LIV | 145 | LIV145_HLJ7VDRXX_CGCGGA-TAGGGC_L002 | 8277286 | 5562401 |
|  | **Total LIV** |  |  | **308956922** | **183456598** |
| High | PIT | 1 | PIT1_HLJ7VDRXX_CAGCGT-ATTTTA_L002 | 13432277 | 8879464 |
| High | PIT | 2 | PIT2_HK5YKDRXX_GATCAC-ATTTTA_L001 | 4853238 | 3633884 |
| High | PIT | 3 | PIT3_HK5YKDRXX_ACCAGT-ATTTTA_L001 | 5137268 | 3115574 |
| High | PIT | 4 | PIT4_HK5YKDRXX_TGCACG-ATTTTA_L001 | 5400811 | 3424312 |
| High | PIT | 6 | PIT6_HK5YKDRXX_ACATTA-ATTTTA_L001 | 4845146 | 3111794 |
| High | PIT | 7 | PIT7_HLJ7VDRXX_GTGTAG-ATTTTA_L002 | 10886061 | 6897660 |
| High | PIT | 8 | PIT8_HLJ7VDRXX_CTAGTC-ATTTTA_L002 | 9822216 | 6437852 |
| High | PIT | 9 | PIT9_HLJ7VDRXX_TGTGCA-ATTTTA_L002 | 11813512 | 7657798 |
| High | PIT | 10 | PIT10_HLJ7VDRXX_TCAGGA-ATTTTA_L002 | 11618137 | 7467565 |
| High | PIT | 11 | PIT11_HK5YKDRXX_CGGTTA-ATTTTA_L001 | 6437083 | 4749544 |
| High | PIT | 31 | PIT31_HK5YKDRXX_GAGTCC-ATTTTA_L001 | 3981069 | 2463893 |
| High | PIT | 32 | PIT32_HK5YKDRXX_GGAGGT-ATTTTA_L001 | 4331897 | 2070447 |
| High | PIT | 33 | PIT33_HLJ7VDRXX_CACACT-ATTTTA_L002 | 9586997 | 5544328 |
| High | PIT | 34 | PIT34_HLJ7VDRXX_CCGCAA-ATTTTA_L002 | 10474169 | 6446749 |
| High | PIT | 35 | PIT35_HK5YKDRXX_TTTATG-ATTTTA_L001 | 4796600 | 3607182 |
| High | PIT | 36 | PIT36_HK5YKDRXX_AACGCC-ATTTTA_L001 | 5435342 | 2909860 |
| High | PIT | 37 | PIT37_HK5YKDRXX_CAAGCA-ATTTTA_L001 | 4837776 | 2357764 |
| High | PIT | 38 | PIT38_HK5YKDRXX_GCTCGA-ATTTTA_L001 | 4802814 | 2365927 |
| High | PIT | 39 | PIT39_HK5YKDRXX_GCGAAT-ATTTTA_L001 | 5481132 | 3354317 |
| High | PIT | 40 | PIT40_HK5YKDRXX_TGGATT-ATTTTA_L001 | 6476090 | 4514125 |
| High | PIT | 46 | PIT46_HLJ7VDRXX_ACCTAC-ATTTTA_L002 | 9433598 | 6296230 |
| High | PIT | 47 | PIT47_HLJ7VDRXX_CGAAGG-ATTTTA_L002 | 12341945 | 8307862 |
| High | PIT | 48 | PIT48_HK5YKDRXX_AGATAG-ATTTTA_L001 | 6233049 | 4324411 |
| High | PIT | 49 | PIT49_HK5YKDRXX_TTGGTA-ATTTTA_L001 | 4803215 | 2996510 |
| High | PIT | 50 | PIT50_HK5YKDRXX_GTTACC-ATTTTA_L001 | 4570258 | 2787105 |
| High | PIT | 51 | PIT51_HK5YKDRXX_CGCAAC-ATTTTA_L001 | 5316921 | 3058794 |
| High | PIT | 52 | PIT52_HK5YKDRXX_TGGCGA-ATTTTA_L001 | 5321161 | 3326371 |
| High | PIT | 53 | PIT53_HLJ7VDRXX_ACCGTG-ATTTTA_L002 | 11470368 | 7045917 |
| High | PIT | 54 | PIT54_HK5YKDRXX_CAACAG-ATTTTA_L001 | 4401846 | 2721774 |
| High | PIT | 55 | PIT55_HLJ7VDRXX_GATTGT-ATTTTA_L002 | 11584560 | 7553929 |
| High | PIT | 91 | PIT91_HK5YKDRXX_GAAGTG-ATTTTA_L001 | 5122838 | 3560813 |
| High | PIT | 92 | PIT92_HK5YKDRXX_CAATGC-ATTTTA_L001 | 4454962 | 2752380 |
| High | PIT | 93 | PIT93_HK5YKDRXX_ACGTCT-ATTTTA_L001 | 5765159 | 3796254 |
| High | PIT | 94 | PIT94_HK5YKDRXX_CAGGAC-ATTTTA_L001 | 5601203 | 3961990 |
| High | PIT | 95 | PIT95_HK5YKDRXX_AAGCTC-ATTTTA_L001 | 5075791 | 2826763 |
| High | PIT | 96 | PIT96_HK5YKDRXX_GACGAT-ATTTTA_L001 | 5497602 | 3113473 |
| High | PIT | 97 | PIT97_HK5YKDRXX_TCGTTC-ATTTTA_L001 | 4953838 | 3194847 |
| High | PIT | 98 | PIT98_HK5YKDRXX_CCAATT-ATTTTA_L001 | 4620210 | 2941476 |
| High | PIT | 99 | PIT99_HK5YKDRXX_AGTTGA-ATTTTA_L001 | 4719593 | 2789018 |
| High | PIT | 100 | PIT100_HK5YKDRXX_AACCGA-ATTTTA_L001 | 4436333 | 2917712 |
| High | PIT | 136 | PIT136_HK5YKDRXX_GAACCT-ATTTTA_L001 | 5622671 | 3655631 |
| High | PIT | 137 | PIT137_HK5YKDRXX_TTCGAG-ATTTTA_L001 | 6295154 | 4392843 |
| High | PIT | 138 | PIT138_HK5YKDRXX_AGAATC-ATTTTA_L001 | 5891870 | 3902628 |
| High | PIT | 139 | PIT139_HK5YKDRXX_AGGCAT-ATTTTA_L001 | 6736636 | 3687532 |
| High | PIT | 140 | PIT140_HLJ7VDRXX_ACACGC-ATTTTA_L002 | 9914550 | 6174432 |
| High | PIT | 141 | PIT141_HLJ7VDRXX_GCGCTG-TAGGGC_L002 | 9071190 | 6348118 |
| High | PIT | 142 | PIT142_HLJ7VDRXX_GAACCT-TAGGGC_L002 | 2480151 | 1632468 |
| High | PIT | 143 | PIT143_HLJ7VDRXX_TTCGAG-TAGGGC_L002 | 8374894 | 5760544 |
| High | PIT | 144 | PIT144_HLJ7VDRXX_AGAATC-TAGGGC_L002 | 9096278 | 6278823 |
| High | PIT | 145 | PIT145_HLJ7VDRXX_AGGCAT-TAGGGC_L002 | 9652198 | 6754247 |
|  | **Total PIT** |  |  | **343309677** | **219870934** |
| Low | LIV | 16 | LIV16_HJK3MDRXX_TTAACT-CGCCAT_L001 | 4823833 | 2403971 |
| Low | LIV | 17 | LIV17_HJK3MDRXX_ATGAAC-CGCCAT_L001 | 4337448 | 1648408 |
| Low | LIV | 18 | LIV18_HJK3MDRXX_CCTAAG-CGCCAT_L001 | 4671360 | 1958490 |
| Low | LIV | 19 | LIV19_HJK3MDRXX_AATCCG-CGCCAT_L001 | 3999766 | 1814881 |
| Low | LIV | 20 | LIV20_HJK3MDRXX_GGCTGC-CGCCAT_L001 | 5382017 | 2568944 |
| Low | LIV | 21 | LIV21_HJK3MDRXX_TACCTT-CGCCAT_L001 | 5741947 | 3620733 |
| Low | LIV | 22 | LIV22_HJK3MDRXX_TCTTAA-CGCCAT_L001 | 3489434 | 2142362 |
| Low | LIV | 23 | LIV23_HJK3MDRXX_GTCAGG-CGCCAT_L001 | 4743705 | 2527064 |
| Low | LIV | 24 | LIV24_HJK3MDRXX_ATACTG-CGCCAT_L001 | 4459765 | 2430626 |
| Low | LIV | 25 | LIV25_HJK3MDRXX_TATGTC-CGCCAT_L001 | 3803018 | 2081202 |
| Low | LIV | 62 | LIV62_HLJ7VDRXX_GCCACA-TAGGGC_L002 | 8477747 | 5987908 |
| Low | LIV | 63 | LIV63_HJK3MDRXX_TGACAC-CGCCAT_L001 | 4166252 | 2372257 |
| Low | LIV | 65 | LIV65_HJK3MDRXX_AAGACA-CGCCAT_L001 | 3876363 | 1763965 |
| Low | LIV | 66 | LIV66_HJK3MDRXX_ACAGAT-CGCCAT_L001 | 3530523 | 1562356 |
| Low | LIV | 67 | LIV67_HJK3MDRXX_TAGGCT-CGCCAT_L001 | 4534424 | 2187272 |
| Low | LIV | 68 | LIV68_HJK3MDRXX_CTCCAT-CGCCAT_L001 | 4993988 | 2700680 |
| Low | LIV | 69 | LIV69_HJK3MDRXX_GCATGG-CGCCAT_L001 | 4311364 | 2250275 |
| Low | LIV | 70 | LIV70_HJK3MDRXX_AATAGC-CGCCAT_L001 | 3711173 | 2500527 |
| Low | LIV | 71 | LIV71_HLJ7VDRXX_CATCTA-TAGGGC_L002 | 8165073 | 5820597 |
| Low | LIV | 72 | LIV72_HJK3MDRXX_TCGAGG-CGCCAT_L001 | 5260440 | 3078936 |
| Low | LIV | 76 | LIV76_HJK3MDRXX_CACTAA-CGCCAT_L001 | 5579751 | 3277151 |
| Low | LIV | 77 | LIV77_HJK3MDRXX_GGTATA-CGCCAT_L001 | 4501929 | 2457252 |
| Low | LIV | 78 | LIV78_HJK3MDRXX_CGCCTG-CGCCAT_L001 | 6472332 | 4030989 |
| Low | LIV | 79 | LIV79_HJK3MDRXX_AATGAA-CGCCAT_L001 | 5099585 | 2914427 |
| Low | LIV | 80 | LIV80_HJK3MDRXX_ACAACG-CGCCAT_L001 | 5483025 | 2777073 |
| Low | LIV | 81 | LIV81_HJK3MDRXX_ATATCC-CGCCAT_L001 | 5224837 | 2767303 |
| Low | LIV | 82 | LIV82_HLJ7VDRXX_AACAAG-TAGGGC_L002 | 9164420 | 6586139 |
| Low | LIV | 83 | LIV83_HJK3MDRXX_ATAAGA-CGCCAT_L001 | 5067208 | 3107311 |
| Low | LIV | 84 | LIV84_HJK3MDRXX_GGTGAG-CGCCAT_L001 | 5567849 | 3275886 |
| Low | LIV | 85 | LIV85_HJK3MDRXX_TTCCGC-CGCCAT_L001 | 5343102 | 3374881 |
| Low | LIV | 106 | LIV106_HJK3MDRXX_CAGATG-CGCCAT_L001 | 4958651 | 2634890 |
| Low | LIV | 107 | LIV107_HJK3MDRXX_GTAGAA-CGCCAT_L001 | 4333018 | 2810050 |
| Low | LIV | 108 | LIV108_HJK3MDRXX_GACATC-CGCCAT_L001 | 7185312 | 4406526 |
| Low | LIV | 109 | LIV109_HJK3MDRXX_CGATCT-CGCCAT_L001 | 6004230 | 3608176 |
| Low | LIV | 110 | LIV110_HLJ7VDRXX_GCAGCC-TAGGGC_L002 | 7554138 | 4902910 |
| Low | LIV | 111 | LIV111_HJK3MDRXX_ATGGCG-CGCCAT_L001 | 5030287 | 3039609 |
| Low | LIV | 112 | LIV112_HJK3MDRXX_ATTGGT-CGCCAT_L001 | 5217047 | 1960290 |
| Low | LIV | 113 | LIV113_HJK3MDRXX_GCCACA-CGCCAT_L001 | 5115480 | 2772022 |
| Low | LIV | 114 | LIV114_HLJ7VDRXX_ACTCTT-TAGGGC_L002 | 8552909 | 5841585 |
| Low | LIV | 115 | LIV115_HJK3MDRXX_AACAAG-CGCCAT_L001 | 5186807 | 3258396 |
| Low | LIV | 121 | LIV121_HLJ7VDRXX_TGCTAT-TAGGGC_L002 | 10154338 | 7387751 |
| Low | LIV | 122 | LIV122_HJK3MDRXX_ACTCTT-CGCCAT_L001 | 6501352 | 3992482 |
| Low | LIV | 123 | LIV123_HJK3MDRXX_TGCTAT-CGCCAT_L001 | 5788153 | 2693540 |
| Low | LIV | 124 | LIV124_HJK3MDRXX_AAGTGG-CGCCAT_L001 | 5253671 | 2729245 |
| Low | LIV | 125 | LIV125_HJK3MDRXX_CTCATA-CGCCAT_L001 | 5884769 | 2980405 |
| Low | LIV | 126 | LIV126_HJK3MDRXX_CCGACC-CGCCAT_L001 | 5670948 | 2763997 |
| Low | LIV | 127 | LIV127_HJK3MDRXX_GGCCAA-CGCCAT_L001 | 5136309 | 3063042 |
| Low | LIV | 128 | LIV128_HLJ7VDRXX_AAGTGG-TAGGGC_L002 | 7808118 | 4861731 |
| Low | LIV | 129 | LIV129_HJK3MDRXX_CGCGGA-CGCCAT_L001 | 3796004 | 1334639 |
| Low | LIV | 130 | LIV130_HJK3MDRXX_CCTGCT-CGCCAT_L001 | 5958422 | 3292556 |
|  | **Total LIV** |  |  | **275073641** | **158323708** |
| Low | PIT | 16 | PIT16_HK5YKDRXX_TTAACT-ATTTTA_L001 | 5467022 | 4009780 |
| Low | PIT | 17 | PIT17_HK5YKDRXX_ATGAAC-ATTTTA_L001 | 4669964 | 2284705 |
| Low | PIT | 18 | PIT18_HK5YKDRXX_CCTAAG-ATTTTA_L001 | 4151268 | 2795965 |
| Low | PIT | 19 | PIT19_HK5YKDRXX_AATCCG-ATTTTA_L001 | 3911401 | 2234322 |
| Low | PIT | 20 | PIT20_HLJ7VDRXX_GGCTGC-ATTTTA_L002 | 10269705 | 7319872 |
| Low | PIT | 21 | PIT21_HLJ7VDRXX_TACCTT-ATTTTA_L002 | 14558889 | 9169010 |
| Low | PIT | 22 | PIT22_HLJ7VDRXX_TCTTAA-ATTTTA_L002 | 9544396 | 6107403 |
| Low | PIT | 23 | PIT23_HK5YKDRXX_GTCAGG-ATTTTA_L001 | 4762037 | 2508317 |
| Low | PIT | 24 | PIT24_HK5YKDRXX_ATACTG-ATTTTA_L001 | 3898742 | 2030679 |
| Low | PIT | 25 | PIT25_HK5YKDRXX_TATGTC-ATTTTA_L001 | 3863957 | 1897083 |
| Low | PIT | 62 | PIT62_HLJ7VDRXX_CCTGCT-TAGGGC_L002 | 9094323 | 6045749 |
| Low | PIT | 63 | PIT63_HK5YKDRXX_TGACAC-ATTTTA_L001 | 5721696 | 4019789 |
| Low | PIT | 65 | PIT65_HK5YKDRXX_AAGACA-ATTTTA_L001 | 4874301 | 2658988 |
| Low | PIT | 66 | PIT66_HK5YKDRXX_ACAGAT-ATTTTA_L001 | 5141674 | 2834253 |
| Low | PIT | 67 | PIT67_HK5YKDRXX_TAGGCT-ATTTTA_L001 | 4269848 | 2699476 |
| Low | PIT | 68 | PIT68_HK5YKDRXX_CTCCAT-ATTTTA_L001 | 5723746 | 3627050 |
| Low | PIT | 69 | PIT69_HLJ7VDRXX_GCATGG-ATTTTA_L002 | 7881872 | 5051975 |
| Low | PIT | 70 | PIT70_HLJ7VDRXX_AATAGC-ATTTTA_L002 | 11570251 | 8571817 |
| Low | PIT | 71 | PIT71_HLJ7VDRXX_GTGCCA-ATTTTA_L002 | 10110292 | 6499127 |
| Low | PIT | 72 | PIT72_HK5YKDRXX_TCGAGG-ATTTTA_L001 | 5391656 | 3080797 |
| Low | PIT | 76 | PIT76_HK5YKDRXX_CACTAA-ATTTTA_L001 | 5181429 | 3145134 |
| Low | PIT | 77 | PIT77_HK5YKDRXX_GGTATA-ATTTTA_L001 | 4945911 | 3146382 |
| Low | PIT | 78 | PIT78_HK5YKDRXX_CGCCTG-ATTTTA_L001 | 5162344 | 3161321 |
| Low | PIT | 79 | PIT79_HLJ7VDRXX_AATGAA-ATTTTA_L002 | 9729050 | 4845352 |
| Low | PIT | 80 | PIT80_HLJ7VDRXX_ACAACG-ATTTTA_L002 | 8284310 | 5053056 |
| Low | PIT | 81 | PIT81_HLJ7VDRXX_ATATCC-ATTTTA_L002 | 9902914 | 5710433 |
| Low | PIT | 82 | PIT82_HLJ7VDRXX_AGTACT-ATTTTA_L002 | 12329918 | 8102298 |
| Low | PIT | 83 | PIT83_HK5YKDRXX_ATAAGA-ATTTTA_L001 | 6341812 | 4444123 |
| Low | PIT | 84 | PIT84_HK5YKDRXX_GGTGAG-ATTTTA_L001 | 5556267 | 3855729 |
| Low | PIT | 85 | PIT85_HK5YKDRXX_TTCCGC-ATTTTA_L001 | 4759376 | 3445445 |
| Low | PIT | 106 | PIT106_HK5YKDRXX_CAGATG-ATTTTA_L001 | 4893071 | 3367863 |
| Low | PIT | 107 | PIT107_HLJ7VDRXX_GTAGAA-ATTTTA_L002 | 10573942 | 6331547 |
| Low | PIT | 108 | PIT108_HK5YKDRXX_GACATC-ATTTTA_L001 | 5632139 | 4040833 |
| Low | PIT | 109 | PIT109_HK5YKDRXX_CGATCT-ATTTTA_L001 | 5938762 | 3600529 |
| Low | PIT | 110 | PIT110_HK5YKDRXX_CGTCGC-ATTTTA_L001 | 4984011 | 3268796 |
| Low | PIT | 111 | PIT111_HK5YKDRXX_ATGGCG-ATTTTA_L001 | 4589250 | 2723159 |
| Low | PIT | 112 | PIT112_HK5YKDRXX_ATTGGT-ATTTTA_L001 | 4337834 | 2814677 |
| Low | PIT | 113 | PIT113_HK5YKDRXX_GCCACA-ATTTTA_L001 | 4678972 | 2459022 |
| Low | PIT | 114 | PIT114_HK5YKDRXX_CATCTA-ATTTTA_L001 | 4804212 | 3203260 |
| Low | PIT | 115 | PIT115_HLJ7VDRXX_AACAAG-ATTTTA_L002 | 12856011 | 9149586 |
| Low | PIT | 121 | PIT121_HLJ7VDRXX_GCAGCC-ATTTTA_L002 | 10940143 | 6916660 |
| Low | PIT | 122 | PIT122_HK5YKDRXX_ACTCTT-ATTTTA_L001 | 6017608 | 4083129 |
| Low | PIT | 123 | PIT123_HLJ7VDRXX_TGCTAT-ATTTTA_L002 | 14314889 | 7929005 |
| Low | PIT | 124 | PIT124_HK5YKDRXX_AAGTGG-ATTTTA_L001 | 4724754 | 1856561 |
| Low | PIT | 125 | PIT125_HK5YKDRXX_CTCATA-ATTTTA_L001 | 4202101 | 2363070 |
| Low | PIT | 126 | PIT126_HK5YKDRXX_CCGACC-ATTTTA_L001 | 5086820 | 2973554 |
| Low | PIT | 127 | PIT127_HK5YKDRXX_GGCCAA-ATTTTA_L001 | 4746343 | 2559534 |
| Low | PIT | 128 | PIT128_HK5YKDRXX_CGCGGA-ATTTTA_L001 | 5763678 | 3833416 |
| Low | PIT | 129 | PIT129_HK5YKDRXX_CCTGCT-ATTTTA_L001 | 5125328 | 3670833 |
| Low | PIT | 130 | PIT130_HK5YKDRXX_GCGCTG-ATTTTA_L001 | 4928419 | 3342851 |
|  | **Total PIT** |  |  | **336208658** | **210843315** |
|  | **Total High** |  |  | **652266599** | **403327532** |
|  | **Total Low** |  |  | **611282299** | **369167023** |
|  | **Total reads** |  |  | **1263548898** | **772494555** |

Supplementary Table 2: List of the 39 genes found differentially expressed (|log_2_FC| > 0.26, *p*-value < 0.05) in the pituitary gland, in the high versus low condition.

| Gene | ACCNUM | ENTREZ ID | SYMBOL | Log_2_FC High/Low | log CPM | LR | *E*-value | *p*-value | FDR adjusted *p*-value |
| --- | --- | --- | --- | --- | --- | --- | --- | --- | --- |
| g12011 | CCE39580 | 402917 | lhb | 1.67 | 10.50 | 60.58 | 1.41E-45 | 2.00E-12 | 1.72E-08 |
| g14247 | XP_017213687 | 30259 | prph | -1.53 | 3.69 | 56.18 | 2.92E-146 | 2.25E-11 | 9.67E-08 |
| g16082 | AHE93330 | 101887161 | tet2 | -1.33 | 4.73 | 54.93 | 1.70E-62 | 1.13E-09 | 3.23E-06 |
| g27508 | A3KQ55 | 561384 | mepce | -1.94 | 3.32 | 48.53 | 0.00E+00 | 2.30E-09 | 4.94E-06 |
| g17089 | AAI63519 | 403114 | adm2a | -1.57 | 3.06 | 46.06 | 0.00E+00 | 4.02E-08 | 6.92E-05 |
| g18798 | AAV31152 | 402919 | fshb | 1.68 | 12.52 | 40.62 | 4.15E-137 | 6.48E-08 | 9.28E-05 |
| g2742 | XP_017207109 | 566645 | espnlb | -1.96 | 2.54 | 40.62 | 2.54E-17 | 9.94E-08 | 1.22E-04 |
| g369 | XP_005172678 | 563644 | kdm6bb | -0.81 | 8.79 | 37.52 | 0.00E+00 | 3.06E-07 | 3.29E-04 |
| g24133 | AAH49416 | 393952 | sigmar1 | -0.92 | 4.55 | 36.57 | 0.00E+00 | 3.45E-07 | 3.29E-04 |
| g27177 | XP_001334673 | 30746 | tie1 | -2.00 | 2.36 | 36.08 | 0.00E+00 | 4.44E-07 | 3.82E-04 |
| g20265 | XP_001920640 | 100150283 | loxhd1b | -0.90 | 5.57 | 35.36 | 1.10E-109 | 9.16E-07 | 6.76E-04 |
| g14339 | CAD60658 | 368823 | fkbp11 | 0.64 | 8.84 | 35.24 | 1.14E-23 | 9.44E-07 | 6.76E-04 |
| g23237 | XP_005174156 | 100330283 | LOC100330283 | -1.09 | 4.03 | 35.46 | 3.13E-28 | 1.59E-06 | 1.05E-03 |
| g10829 | AAH59702 | 793658 | arl13a | -1.33 | 4.18 | 34.39 | 9.07E-52 | 3.18E-06 | 1.95E-03 |
| g10971 | XP_009289566 | 103908779 | c14h4orf45 | -0.82 | 6.18 | 34.08 | 1.36E-20 | 3.95E-06 | 2.26E-03 |
| g12861 | XP_021325885 | 565402 | col11a1a | 1.22 | 3.55 | 33.73 | 6.23E-09 | 4.41E-06 | 2.37E-03 |
| g25215 | NP_956951 | 393630 | thyn1 | -0.88 | 4.20 | 32.17 | 2.38E-72 | 5.74E-06 | 2.87E-03 |
| g27504 | XP_017210159 | 100331067 | LOC100331067 | -0.81 | 5.28 | 32.12 | 0.00E+00 | 6.01E-06 | 2.87E-03 |
| g4773 | AAH92763 | 550548 | stmn1b | 0.78 | 5.93 | 31.72 | 1.95E-110 | 1.33E-05 | 6.02E-03 |
| g25621 | XP_017207641 | 108180053 | LOC108180053 | 0.76 | 5.27 | 31.47 | 1.28E-24 | 1.90E-05 | 8.15E-03 |
| g24169 | XP_021324735 | 793912 | nefla | 1.18 | 4.22 | 31.35 | 5.27E-32 | 2.94E-05 | 1.20E-02 |
| g10394 | NP_001131027 | 798788 | gpx3 | 0.78 | 8.91 | 31.05 | 0.00E+00 | 3.16E-05 | 1.23E-02 |
| g10440 | NP_001338645 | 568796 | pcdh20 | 0.98 | 3.94 | 28.65 | 1.04E-68 | 3.32E-05 | 1.24E-02 |
| g24427 | ABB60051 | 493623 | gpsm1b | -0.63 | 6.58 | 28.35 | 0.00E+00 | 3.82E-05 | 1.37E-02 |
| g25508 | NP_001119871 | 561195 | trpv1 | 0.92 | 4.76 | 27.31 | 0.00E+00 | 4.33E-05 | 1.49E-02 |
| g24324 | AAH92881 | 550498 | iscub | -1.06 | 5.26 | 26.64 | 0.00E+00 | 6.49E-05 | 2.14E-02 |
| g2685 | AAI25914 | 777641 | scg2b | -0.99 | 5.53 | 26.00 | 7.48E-44 | 7.79E-05 | 2.48E-02 |
| g18777 | XP_005166545 | 564462 | lrrc4cb | 0.93 | 4.36 | 26.06 | 3.53E-155 | 8.44E-05 | 2.59E-02 |
| g434 | AAH92842 | 572669 | dnajb13 | -1.41 | 3.39 | 25.37 | 3.30E-19 | 9.33E-05 | 2.76E-02 |
| g15780 | XP_005171649 | 793920 | il2rb | 1.50 | 2.16 | 23.46 | 0.00E+00 | 9.63E-05 | 2.76E-02 |
| g6818 | XP_005173575 | 572411 | ephb6 | 0.66 | 6.10 | 23.34 | 0.00E+00 | 1.50E-04 | 4.16E-02 |
| g19448 | AAH61705 | 494154 | lancl1 | -0.78 | 4.25 | 22.71 | 0.00E+00 | 1.55E-04 | 4.16E-02 |
| g11927 | AAD47423 | 30645 | cd74b | 0.90 | 5.00 | 22.57 | 0.00E+00 | 1.81E-04 | 4.70E-02 |
| g11419 | NP_001026843 | 791878 | cst3 | -1.02 | 8.18 | 22.17 | 0.00E+00 | 1.88E-04 | 4.72E-02 |
| g14935 | AAH56531 | 321818 | ctsa | -0.80 | 4.69 | 21.70 | 0.00E+00 | 1.98E-04 | 4.72E-02 |
| g25691 | XP_009292131 | 100034387 | sphkap | -0.77 | 5.00 | 21.80 | 3.62E-82 | 1.99E-04 | 4.72E-02 |
| g2899 | XP_017207706 | 563528 | prss56 | -1.29 | 2.59 | 21.72 | 0.00E+00 | 2.03E-04 | 4.72E-02 |
| g17678 | XP_009301186 | 103911343 | si:ch1073-153i20.4 | 0.58 | 6.79 | 21.53 | 0.00E+00 | 2.14E-04 | 4.85E-02 |
| g4921 | BAF47195 | 564619 | gstr | -0.97 | 6.75 | 21.18 | 4.30E-173 | 2.22E-04 | 4.90E-02 |

Supplementary Table 3: List of the 238 genes found differentially expressed in the liver (|log_2_FC| > 0.26, *p*-value < 0.05) in the high versus low condition.

| Gene | ACCNUM | ENTREZ ID | SYMBOL | Log_2_FC High/ Low | log CPM | LR | *E*-value | *p*-value | FDR adjusted *p*-value |
| --- | --- | --- | --- | --- | --- | --- | --- | --- | --- |
| g7896 | AAI24470 | 558677 | zp3a.2 | -1.76 | 6.51 | 131.08 | 3.57E-114 | 4.25E-24 | 3.66E-20 |
| g10950 | AAH59459 | 317738 | elovl6 | -2.46 | 6.97 | 109.04 | 0.00E+00 | 2.04E-22 | 8.78E-19 |
| g4294 | NP_942110 | 386661 | scd | -1.92 | 7.09 | 79.51 | 1.14E-163 | 4.29E-16 | 1.23E-12 |
| g11736 | A4QP75 | 606657 | trmt2b | -1.60 | 7.00 | 79.84 | 0.00E+00 | 2.70E-15 | 5.80E-12 |
| g3745 | XP_009305081 | 559001 | fasn | -2.66 | 3.45 | 80.31 | 0.00E+00 | 3.76E-14 | 6.46E-11 |
| g25374 | AAI62122 | 568167 | thrsp | -1.92 | 10.97 | 74.52 | 0.00E+00 | 5.07E-13 | 7.27E-10 |
| g9018 | AAX45073 | 394021 | shmt1 | -1.17 | 5.95 | 69.32 | 1.01E-76 | 8.54E-13 | 1.05E-09 |
| g21643 | AAF79948 | 58128 | fabp7a | -2.22 | 6.44 | 69.01 | 5.20E-13 | 1.32E-12 | 1.42E-09 |
| g25034 | XP_005157555 | 100037332 | zgc:162608 | 1.27 | 8.24 | 67.84 | 6.29E-84 | 4.16E-12 | 3.98E-09 |
| g3123 | AAI50352 | 567406 | ahsg2 | 1.13 | 11.00 | 67.14 | 8.16E-158 | 7.52E-12 | 6.46E-09 |
| g19174 | XP_003199513 | 571189 | abcb11b | -1.20 | 5.37 | 66.75 | 3.18E-58 | 1.83E-11 | 1.43E-08 |
| g20728 | XP_005171883 | 406312 | zfand5a | 1.03 | 7.22 | 65.63 | 2.21E-36 | 4.03E-11 | 2.86E-08 |
| g6991 | AAH56293 | 793727 | gnl2 | 0.83 | 6.41 | 63.90 | 0.00E+00 | 4.33E-11 | 2.86E-08 |
| g9808 | AAH76484 | 436922 | aclya | -3.36 | 3.84 | 63.85 | 0.00E+00 | 7.85E-11 | 4.82E-08 |
| g4032 | AAH56596 | 321618 | h1f0 | 1.03 | 5.38 | 61.61 | 1.36E-113 | 8.44E-11 | 4.84E-08 |
| g25035 | XP_696211 | 567816 | LOC567816 | 1.11 | 8.60 | 61.22 | 8.91E-40 | 1.24E-10 | 6.67E-08 |
| g14566 | XP_693183 | 564761 | itih1 | -1.42 | 3.47 | 61.08 | 0.00E+00 | 1.80E-10 | 9.11E-08 |
| g12756 | AAI17630 | 554998 | pdia4 | -1.19 | 5.17 | 57.54 | 1.35E-123 | 2.15E-10 | 1.03E-07 |
| g24011 | CAI99155 | 560202 | ghrb | 1.13 | 4.91 | 57.26 | 1.94E-83 | 2.85E-10 | 1.29E-07 |
| g3399 | BAE78824 | 402819 | cnpy1 | -1.04 | 6.07 | 56.92 | 0.00E+00 | 7.95E-10 | 3.27E-07 |
| g23522 | AAH46004 | 322420 | chia.6 | 1.67 | 3.39 | 56.77 | 2.26E-84 | 7.98E-10 | 3.27E-07 |
| g14734 | XP_002663836 | 100334148 | si:dkey-57n24.6 | 1.55 | 3.03 | 54.11 | 0.00E+00 | 2.03E-09 | 7.92E-07 |
| g24462 | XP_017208526 | 559835 | alpk2 | -2.10 | 2.78 | 54.04 | 0.00E+00 | 3.35E-09 | 1.25E-06 |
| g20065 | XP_005165133 | 393984 | aacs | -1.55 | 5.58 | 52.78 | 0.00E+00 | 4.71E-09 | 1.69E-06 |
| g8240 | AAH91470 | 560651 | ces3 | 0.94 | 7.48 | 52.27 | 2.24E-100 | 6.32E-09 | 2.17E-06 |
| g28139 | CAB64945 | 30708 | fabp2 | -1.26 | 5.84 | 49.78 | 0.00E+00 | 6.67E-09 | 2.21E-06 |
| g20278 | AAH83399 | 450001 | zgc:103482 | -1.55 | 3.61 | 48.31 | 0.00E+00 | 7.65E-09 | 2.44E-06 |
| g15238 | AAH54574 | 64608 | alas1 | 1.04 | 6.75 | 48.20 | 1.23E-105 | 1.48E-08 | 4.56E-06 |
| g20667 | NP_001352946 | 110437731 | LOC110437731 | 0.68 | 13.17 | 48.10 | 2.76E-69 | 2.30E-08 | 6.80E-06 |
| g8257 | NP_001093469 | 560780 | si:dkey-238o13.4 | -2.90 | 3.22 | 47.48 | 0.00E+00 | 2.85E-08 | 8.16E-06 |
| g11458 | ABY90095 | 407621 | disc1 | 0.67 | 6.35 | 47.13 | 9.70E-173 | 3.88E-08 | 1.08E-05 |
| g27504 | XP_017210159 | 100331067 | LOC100331067 | -1.01 | 4.97 | 47.06 | 0.00E+00 | 5.12E-08 | 1.38E-05 |
| g8568 | AAP47138 | 386590 | hsp90b1 | -0.92 | 7.62 | 46.70 | 0.00E+00 | 5.69E-08 | 1.48E-05 |
| g8006 | XP_005159361 | 100321139 | alpk3a | -1.32 | 3.60 | 46.18 | 0.00E+00 | 5.96E-08 | 1.51E-05 |
| g9529 | XP_021323416 | 100007164 | cbln14 | 1.38 | 6.44 | 45.61 | 2.48E-57 | 7.70E-08 | 1.89E-05 |
| g15317 | ABM68033 | 767678 | cish | 1.23 | 5.93 | 44.95 | 0.00E+00 | 8.74E-08 | 2.08E-05 |
| g7092 | AAH76516 | 798836 | tspan13a | -1.17 | 4.33 | 44.88 | 0.00E+00 | 8.96E-08 | 2.08E-05 |
| g9286 | AAI71350 | 573110 | cbx4 | -0.85 | 5.83 | 44.52 | 1.57E-54 | 9.50E-08 | 2.15E-05 |
| g19261 | NP_001314912 | 100537632 | spp2 | 0.96 | 11.24 | 44.17 | 1.36E-20 | 1.02E-07 | 2.25E-05 |
| g4478 | AAH81587 | 447860 | acsl5 | -1.05 | 7.97 | 43.85 | 0.00E+00 | 1.25E-07 | 2.69E-05 |
| g18149 | NP_001038917 | 751742 | uraha | 0.86 | 5.89 | 43.64 | 3.90E-166 | 1.80E-07 | 3.77E-05 |
| g8109 | XP_005174313 | 101887155 | pnpla2 | 1.14 | 3.97 | 43.61 | 0.00E+00 | 2.21E-07 | 4.52E-05 |
| g16082 | AHE93330 | 101887161 | tet2 | -1.10 | 4.57 | 43.52 | 9.61E-23 | 2.64E-07 | 5.28E-05 |
| g387 | XP_017211598 | 559403 | acaca | -1.19 | 4.23 | 42.11 | 0.00E+00 | 3.27E-07 | 6.39E-05 |
| g655 | NP_001313465 | 556700 | smpd1 | -1.01 | 5.08 | 41.94 | 0.00E+00 | 4.34E-07 | 8.30E-05 |
| g21862 | XP_009291550 | 101883329 | si:dkey-206p8.1 | 1.17 | 4.41 | 41.77 | 0.00E+00 | 4.45E-07 | 8.32E-05 |
| g18087 | AAH65657 | 324010 | hnf4b | 0.98 | 5.30 | 41.48 | 0.00E+00 | 5.10E-07 | 9.29E-05 |
| g26929 | AAH76466 | 436604 | uox | 0.75 | 11.12 | 41.23 | 0.00E+00 | 5.18E-07 | 9.29E-05 |
| g4039 | XP_005162937 | 406623 | decr2 | -0.73 | 7.13 | 41.04 | 0.00E+00 | 6.20E-07 | 1.09E-04 |
| g16143 | AAI27404 | 777738 | vps37c | 0.97 | 4.41 | 40.80 | 2.92E-109 | 7.49E-07 | 1.29E-04 |
| g27511 | AAQ97775 | 378963 | eno3 | 0.72 | 9.86 | 40.45 | 3.21E-39 | 7.81E-07 | 1.32E-04 |
| g4159 | AAH59706 | 406298 | ndufa4 | -1.40 | 3.94 | 40.07 | 0.00E+00 | 9.75E-07 | 1.61E-04 |
| g3385 | CBN61608 | 405829 | dpydb | 0.64 | 9.27 | 40.04 | 1.44E-80 | 1.01E-06 | 1.63E-04 |
| g11479 | AAI22336 | 563048 | LOC563048 | 0.58 | 11.16 | 39.56 | 0.00E+00 | 1.15E-06 | 1.82E-04 |
| g1602 | XP_685872 | 564300 | lrrc58b | 0.75 | 6.57 | 39.49 | 0.00E+00 | 1.20E-06 | 1.87E-04 |
| g23029 | AAH44196 | 406762 | pgd | -1.38 | 3.52 | 39.30 | 0.00E+00 | 1.25E-06 | 1.92E-04 |
| g3039 | XP_005163400 | 101883396 | LOC101883396 | -1.02 | 4.22 | 39.03 | 0.00E+00 | 1.29E-06 | 1.95E-04 |
| g9623 | XP_005164295 | 572061 | pde4a | -0.71 | 6.01 | 38.52 | 2.46E-118 | 1.33E-06 | 1.97E-04 |
| g22893 | AAI33979 | 449685 | zgc:136410 | -0.87 | 11.44 | 38.39 | 0.00E+00 | 1.53E-06 | 2.22E-04 |
| g21455 | AAI24343 | 767694 | slc25a21 | -0.69 | 6.78 | 38.40 | 5.75E-73 | 1.74E-06 | 2.49E-04 |
| g5686 | AAI28792 | 664756 | eef1a1l2 | -1.20 | 3.80 | 38.10 | 1.73E-59 | 2.35E-06 | 3.31E-04 |
| g13701 | XP_002665852 | 100330435 | apobb.2 | -1.62 | 2.93 | 37.52 | 4.70E-49 | 2.55E-06 | 3.54E-04 |
| g325 | XP_009299903 | 393246 | ncor1 | 0.61 | 7.49 | 37.51 | 2.78E-159 | 2.65E-06 | 3.61E-04 |
| g18698 | AAQ97835 | 393922 | psmd13 | -0.93 | 5.10 | 37.33 | 0.00E+00 | 2.78E-06 | 3.74E-04 |
| g24409 | AAT68067 | 378848 | hspa5 | -1.36 | 3.55 | 37.21 | 0.00E+00 | 3.44E-06 | 4.54E-04 |
| g16372 | XP_021332635 | 100535713 | LOC100535713 | -0.72 | 5.67 | 37.06 | 0.00E+00 | 3.50E-06 | 4.56E-04 |
| g24540 | AAH97172 | 327642 | lrrc8aa | -1.04 | 4.28 | 36.93 | 1.45E-46 | 3.57E-06 | 4.58E-04 |
| g2827 | XP_699339 | 570735 | dhx30 | -1.37 | 3.36 | 36.60 | 7.83E-175 | 3.92E-06 | 4.96E-04 |
| g26590 | NP_694486 | 259306 | cyp2ad2 | 0.61 | 9.22 | 36.59 | 0.00E+00 | 4.73E-06 | 5.89E-04 |
| g22283 | XP_005158606 | 556848 | crybg1a | 1.01 | 4.79 | 36.31 | 4.36E-59 | 5.65E-06 | 6.94E-04 |
| g16832 | ACR83585 | 407086 | junba | 0.99 | 7.59 | 36.28 | 1.51E-134 | 5.83E-06 | 7.05E-04 |
| g18101 | AAH53243 | 393997 | lipca | 0.73 | 6.30 | 36.16 | 0.00E+00 | 7.08E-06 | 8.46E-04 |
| g17288 | AAH71313 | 415138 | akr1b1.1 | 0.73 | 4.96 | 36.02 | 1.40E+00 | 7.86E-06 | 9.26E-04 |
| g27488 | AAH76027 | 436717 | decr1 | 0.68 | 7.05 | 35.88 | 4.38E-27 | 8.33E-06 | 9.67E-04 |
| g1071 | AAH81582 | 447800 | serpinf1 | 0.88 | 6.72 | 35.82 | 0.00E+00 | 8.44E-06 | 9.67E-04 |
| g25215 | NP_956951 | 393630 | thyn1 | -1.01 | 4.08 | 35.23 | 4.41E-24 | 9.74E-06 | 1.10E-03 |
| g6943 | CAB64946 | 30314 | apoeb | -1.33 | 3.45 | 35.23 | 6.73E-149 | 1.01E-05 | 1.13E-03 |
| g25870 | AAI24635 | 569462 | pnkp | 0.64 | 6.34 | 35.02 | 0.00E+00 | 1.04E-05 | 1.14E-03 |
| g3175 | AAH49037 | 406736 | mgst3a | -0.86 | 5.55 | 34.66 | 4.42E-149 | 1.11E-05 | 1.21E-03 |
| g20287 | AAU14811 | 402991 | gadd45ga | 1.16 | 3.68 | 34.11 | 0.00E+00 | 1.32E-05 | 1.42E-03 |
| g9705 | XP_021326410 | 553550 | tsen54 | -1.92 | 3.25 | 33.58 | 5.85E-151 | 1.48E-05 | 1.55E-03 |
| g19902 | AAI25920 | 777717 | bmp3 | 0.91 | 5.13 | 33.51 | 0.00E+00 | 1.48E-05 | 1.55E-03 |
| g18397 | NP_001139486 | 555721 | sall1a | 0.65 | 8.92 | 33.43 | 2.80E+00 | 1.51E-05 | 1.56E-03 |
| g14750 | AAH93358 | 550453 | pdrg1 | -1.36 | 3.42 | 33.44 | 2.19E-130 | 1.72E-05 | 1.76E-03 |
| g26451 | AAH48051 | 321193 | paics | 0.69 | 7.47 | 33.28 | 0.00E+00 | 1.77E-05 | 1.79E-03 |
| g25869 | AAI39710 | 563828 | si:ch1073-280e3.1 | 0.63 | 6.03 | 33.28 | 1.26E-52 | 1.84E-05 | 1.84E-03 |
| g956 | AAH47807 | 327133 | hyou1 | -0.71 | 5.61 | 32.99 | 0.00E+00 | 2.18E-05 | 2.15E-03 |
| g879 | XP_002663594 | 378983 | postna | 1.10 | 3.08 | 32.83 | 2.36E-141 | 2.21E-05 | 2.15E-03 |
| g24648 | XP_021331726 | 404040 | dab2 | -0.72 | 4.86 | 32.83 | 2.11E-142 | 2.22E-05 | 2.15E-03 |
| g20074 | AAH57478 | 321621 | gldc | -0.84 | 4.12 | 32.72 | 0.00E+00 | 2.34E-05 | 2.22E-03 |
| g11153 | XP_005156656 | 492518 | haao | 0.68 | 5.62 | 32.69 | 0.00E+00 | 2.37E-05 | 2.22E-03 |
| g25356 | XP_009289773 | 559212 | gbe1b | -1.00 | 3.29 | 32.12 | 0.00E+00 | 2.37E-05 | 2.22E-03 |
| g13654 | AAH60895 | 335799 | sod2 | -0.96 | 4.40 | 32.03 | 2.09E-80 | 2.48E-05 | 2.29E-03 |
| g6972 | XP_005157915 | 553294 | ube3d | -1.27 | 2.60 | 31.84 | 1.86E-41 | 2.51E-05 | 2.30E-03 |
| g25800 | NP_001107918 | 573117 | tbrg1 | -1.01 | 3.12 | 31.48 | 9.55E-10 | 2.62E-05 | 2.36E-03 |
| g4503 | F1QGH9 | 322265 | psmd11b | -1.31 | 2.45 | 31.40 | 1.03E-146 | 2.64E-05 | 2.36E-03 |
| g26744 | ABF18955 | 393108 | cyp2p6 | -0.75 | 5.91 | 30.54 | 0.00E+00 | 2.90E-05 | 2.57E-03 |
| g8102 | AAG25710 | 140615 | fads2 | -2.88 | 2.25 | 30.43 | 0.00E+00 | 2.98E-05 | 2.61E-03 |
| g16403 | XP_001921144 | 799340 | si:ch211-207j7.2 | 0.87 | 6.03 | 30.29 | 0.00E+00 | 3.02E-05 | 2.63E-03 |
| g2348 | AAH75752 | 436592 | stard3nl | -0.61 | 7.59 | 30.22 | 3.88E-144 | 3.22E-05 | 2.77E-03 |
| g10578 | AAQ94592 | 321664 | aldob | 0.61 | 12.81 | 30.24 | 0.00E+00 | 3.31E-05 | 2.82E-03 |
| g5490 | CAK04314 | 338267 | arl4ab | 0.97 | 3.73 | 30.16 | 1.10E-76 | 3.50E-05 | 2.93E-03 |
| g145 | ABA54856 | 566735 | insb | 0.85 | 5.84 | 30.08 | 0.00E+00 | 3.51E-05 | 2.93E-03 |
| g24324 | AAH92881 | 550498 | iscub | -1.14 | 4.92 | 29.91 | 0.00E+00 | 4.20E-05 | 3.47E-03 |
| g17079 | AAH53187 | 393941 | psmc2 | -0.60 | 7.80 | 29.79 | 2.19E-61 | 4.28E-05 | 3.50E-03 |
| g23232 | AAI62754 | 100005854 | zgc:194242 | 0.87 | 4.30 | 29.63 | 2.29E-85 | 4.50E-05 | 3.65E-03 |
| g21132 | AAI53957 | 561171 | cbln8 | -1.16 | 11.60 | 29.50 | 2.99E-64 | 4.63E-05 | 3.72E-03 |
| g1559 | AAH71315 | 321611 | mpc2 | -0.70 | 6.32 | 29.40 | 0.00E+00 | 4.89E-05 | 3.89E-03 |
| g15959 | AAI25969 | 558851 | cyp4v8 | 0.84 | 5.61 | 29.22 | 7.14E-56 | 5.30E-05 | 4.18E-03 |
| g23424 | AAH65350 | 402927 | zgc:77375 | -1.50 | 2.17 | 29.15 | 0.00E+00 | 5.80E-05 | 4.50E-03 |
| g5066 | ACX31197 | 560713 | ccn4a | -1.27 | 2.68 | 28.98 | 5.68E-130 | 5.82E-05 | 4.50E-03 |
| g13775 | XP_021322709 | 100536500 | LOC100536500 | -1.92 | 4.34 | 28.81 | 3.40E-149 | 6.08E-05 | 4.66E-03 |
| g21531 | AAH79506 | 445316 | cdkl1 | 0.83 | 4.33 | 28.78 | 0.00E+00 | 6.94E-05 | 5.28E-03 |
| g9736 | AAH81493 | 447942 | cox6a2 | 0.65 | 7.70 | 28.70 | 0.00E+00 | 7.04E-05 | 5.31E-03 |
| g4921 | BAF47195 | 564619 | gstr | -0.93 | 8.82 | 28.57 | 2.69E-46 | 7.44E-05 | 5.56E-03 |
| g6338 | NP_001313526 | 792958 | LOC792958 | -0.84 | 5.26 | 28.28 | 1.70E-62 | 7.64E-05 | 5.66E-03 |
| g16022 | ABA54452 | 326102 | hhip | 0.73 | 4.57 | 28.22 | 3.10E-132 | 7.75E-05 | 5.69E-03 |
| g17658 | AAI65335 | 114367 | nots | -1.41 | 2.79 | 28.21 | 1.07E-123 | 8.01E-05 | 5.84E-03 |
| g10691 | XP_021336737 | 767699 | pmt | 0.61 | 8.26 | 28.16 | 1.70E-145 | 8.68E-05 | 6.27E-03 |
| g15133 | AAI33741 | 100004700 | cyp24a1 | 0.89 | 6.22 | 28.14 | 0.00E+00 | 9.20E-05 | 6.59E-03 |
| g4380 | Q6PBP3 | 323739 | selenou1a | -0.90 | 7.74 | 27.83 | 2.78E-133 | 9.29E-05 | 6.60E-03 |
| g9193 | AAH71424 | 405830 | lgals2a | -0.79 | 6.78 | 27.73 | 2.55E-99 | 9.48E-05 | 6.68E-03 |
| g21131 | XP_021322670 | 557984 | si:ch211-215k15.4 | 0.63 | 11.34 | 27.63 | 0.00E+00 | 9.86E-05 | 6.89E-03 |
| g2585 | XP_021335998 | 110440142 | LOC110440142 | -1.12 | 3.77 | 27.59 | 0.00E+00 | 1.12E-04 | 7.76E-03 |
| g9592 | XP_005164296 | 101882899 | LOC101882899 | 0.60 | 6.04 | 27.47 | 0.00E+00 | 1.29E-04 | 8.77E-03 |
| g18349 | AAH93190 | 550462 | ubl7a | -1.26 | 2.22 | 27.14 | 0.00E+00 | 1.29E-04 | 8.77E-03 |
| g8532 | AAH76206 | 445033 | psma1 | -0.73 | 5.20 | 26.84 | 0.00E+00 | 1.30E-04 | 8.77E-03 |
| g8230 | AAH65322 | 406571 | utp4 | 0.79 | 3.99 | 26.80 | 0.00E+00 | 1.32E-04 | 8.83E-03 |
| g17216 | XP_003200166 | 100537809 | LOC100537809 | -1.26 | 2.58 | 26.67 | 1.95E-110 | 1.38E-04 | 9.19E-03 |
| g8353 | AAX48021 | 415191 | tnni4b.2 | -0.99 | 4.92 | 26.03 | 8.75E-25 | 1.39E-04 | 9.19E-03 |
| g26613 | XP_005170499 | 373089 | tjp3 | 0.61 | 5.18 | 25.97 | 9.01E-25 | 1.46E-04 | 9.59E-03 |
| g23931 | AAI35051 | 64278 | psmb7 | -0.61 | 6.72 | 25.83 | 1.08E-21 | 1.50E-04 | 9.76E-03 |
| g16607 | XP_002660623 | 100331428 | c3a.4 | 0.63 | 8.42 | 25.83 | 0.00E+00 | 1.51E-04 | 9.78E-03 |
| g21516 | NP_001177311 | 100462964 | selenol | 0.90 | 4.01 | 25.71 | 0.00E+00 | 1.53E-04 | 9.83E-03 |
| g87 | AAH44395 | 406828 | faxdc2 | -0.75 | 5.78 | 25.63 | 0.00E+00 | 1.55E-04 | 9.86E-03 |
| g1405 | AAH56563 | 327588 | hpxa | 0.82 | 10.52 | 25.51 | 0.00E+00 | 1.70E-04 | 1.07E-02 |
| g26283 | AAH68336 | 30248 | calr3a | -0.77 | 4.34 | 25.34 | 2.61E-104 | 1.71E-04 | 1.07E-02 |
| g895 | XP_021335093 | 562316 | kl | 1.09 | 3.00 | 25.17 | 0.00E+00 | 1.81E-04 | 1.13E-02 |
| g13790 | AAI63181 | 792608 | knstrn | -1.53 | 2.60 | 24.87 | 0.00E+00 | 1.83E-04 | 1.13E-02 |
| g2744 | AAH56520 | 79378 | agxtb | 0.62 | 9.65 | 24.76 | 0.00E+00 | 1.86E-04 | 1.14E-02 |
| g19988 | AAL75953 | 140614 | xbp1 | 0.63 | 5.41 | 24.59 | 0.00E+00 | 1.94E-04 | 1.18E-02 |
| g24893 | XP_005172658 | 101886567 | rph3aa | 0.58 | 7.32 | 24.60 | 5.24E-172 | 2.08E-04 | 1.26E-02 |
| g6212 | AAI60631 | 100144569 | dpys | -0.76 | 4.63 | 24.59 | 0.00E+00 | 2.14E-04 | 1.29E-02 |
| g11110 | XP_021336675 | 436640 | slc25a48 | -1.03 | 3.45 | 24.51 | 8.77E-153 | 2.16E-04 | 1.29E-02 |
| g11585 | AAN77156 | 393425 | elovl5 | -1.10 | 2.74 | 24.48 | 1.51E-88 | 2.18E-04 | 1.29E-02 |
| g13695 | XP_017207922 | 321070 | rock2b | -0.75 | 4.06 | 24.41 | 0.00E+00 | 2.19E-04 | 1.29E-02 |
| g24033 | XP_009300100 | 100331745 | npr2 | 0.67 | 4.55 | 24.34 | 0.00E+00 | 2.47E-04 | 1.44E-02 |
| g20245 | AAH91862 | 541493 | lifra | 0.71 | 4.13 | 24.20 | 1.54E-147 | 2.53E-04 | 1.46E-02 |
| g16618 | XP_001345197 | 100006464 | si:dkey-27j5.5 | -1.10 | 2.89 | 23.97 | 0.00E+00 | 2.53E-04 | 1.46E-02 |
| g10296 | XP_005157299 | 563853 | mtus1a | -0.70 | 4.41 | 23.90 | 2.03E-51 | 2.61E-04 | 1.49E-02 |
| g26827 | XP_005166980 | 795505 | eps15 | -0.62 | 5.18 | 23.89 | 0.00E+00 | 2.74E-04 | 1.56E-02 |
| g13534 | AAH65466 | 394198 | fosab | 1.24 | 3.87 | 23.83 | 0.00E+00 | 2.83E-04 | 1.60E-02 |
| g26216 | Q5U3U3 | 100005717 | cpt2 | -1.22 | 2.52 | 23.58 | 0.00E+00 | 2.98E-04 | 1.68E-02 |
| g1072 | AAI28865 | 563663 | serpinf2b | 0.52 | 9.70 | 23.56 | 3.64E-130 | 3.01E-04 | 1.68E-02 |
| g1260 | AAH62829 | 406531 | arl4cb | 0.99 | 3.52 | 23.55 | 1.29E-84 | 3.16E-04 | 1.75E-02 |
| g17519 | XP_005164939 | 563056 | hcls1 | -0.64 | 6.28 | 23.50 | 0.00E+00 | 3.26E-04 | 1.80E-02 |
| g18874 | XP_021333072 | 322372 | fah | -0.95 | 3.11 | 23.46 | 1.19E-168 | 3.29E-04 | 1.80E-02 |
| g19121 | XP_005165919 | 393122 | dnpep | -0.75 | 5.00 | 23.29 | 1.38E-95 | 3.32E-04 | 1.81E-02 |
| g24076 | XP_021327915 | 558050 | si:ch211-127i16.2 | -1.04 | 2.79 | 23.12 | 2.06E-116 | 3.38E-04 | 1.83E-02 |
| g18779 | AAS58450 | 322626 | hsd17b12b | -0.73 | 3.89 | 23.09 | 6.73E-164 | 3.62E-04 | 1.95E-02 |
| g18200 | AAI60629 | 799650 | ddx21 | 0.47 | 12.07 | 23.04 | 0.00E+00 | 3.84E-04 | 2.04E-02 |
| g14735 | AAI17590 | 724002 | acad9 | 0.58 | 5.56 | 22.98 | 1.01E-101 | 3.85E-04 | 2.04E-02 |
| g16510 | AAH42325 | 321898 | psmd12 | -0.73 | 4.25 | 22.90 | 1.42E-124 | 3.87E-04 | 2.04E-02 |
| g14888 | AJG05938 | 767691 | manf | -1.13 | 2.55 | 22.80 | 3.32E-103 | 3.97E-04 | 2.08E-02 |
| g2191 | AAH81413 | 447937 | zgc:101851 | -1.23 | 2.27 | 22.79 | 4.00E-03 | 4.00E-04 | 2.08E-02 |
| g21316 | AAI52167 | 553553 | cfd | 0.98 | 3.82 | 22.77 | 4.59E-83 | 4.06E-04 | 2.10E-02 |
| g19348 | AAI16550 | 692285 | pspc1 | 0.57 | 5.43 | 22.75 | 1.02E-103 | 4.13E-04 | 2.12E-02 |
| g9619 | NP_001315082 | 107990273 | si:dkey-114l24.2 | -0.98 | 4.04 | 22.62 | 0.00E+00 | 4.15E-04 | 2.13E-02 |
| g22962 | AAI24536 | 556929 | zgc:154075 | 0.96 | 3.89 | 22.61 | 0.00E+00 | 4.19E-04 | 2.13E-02 |
| g15589 | XP_005155982 | 664760 | tbce | 0.73 | 4.18 | 22.56 | 3.03E-110 | 4.22E-04 | 2.14E-02 |
| g20488 | NP_001119925 | 793882 | git2a | -1.10 | 2.64 | 22.57 | 0.00E+00 | 4.27E-04 | 2.15E-02 |
| g814 | AAI15276 | 678552 | f9b | 0.47 | 9.04 | 22.58 | 0.00E+00 | 4.29E-04 | 2.15E-02 |
| g7213 | XP_005158307 | 558292 | scap | -1.03 | 2.73 | 22.32 | 0.00E+00 | 4.38E-04 | 2.18E-02 |
| g26222 | NP_001038663 | 570393 | hook1 | -1.17 | 2.74 | 22.24 | 0.00E+00 | 4.43E-04 | 2.19E-02 |
| g21959 | XP_002660625 | 100320176 | si:ch73-343l4.8 | 0.50 | 8.00 | 22.08 | 1.24E-52 | 4.63E-04 | 2.28E-02 |
| g26015 | AAH56516 | 393518 | psmd2 | -0.47 | 7.85 | 22.06 | 1.10E-90 | 4.66E-04 | 2.28E-02 |
| g23354 | AAF63256 | 791729 | rh50 | -1.09 | 3.45 | 21.81 | 0.00E+00 | 5.02E-04 | 2.44E-02 |
| g8514 | AAH75928 | 436610 | pnpla3 | 0.86 | 3.94 | 21.73 | 0.00E+00 | 5.15E-04 | 2.47E-02 |
| g28046 | XP_021333686 | 555504 | pcdh7b | 0.83 | 3.83 | 21.71 | 0.00E+00 | 5.15E-04 | 2.47E-02 |
| g24209 | XP_021331766 | 445388 | rcl1 | 0.55 | 8.36 | 21.70 | 0.00E+00 | 5.22E-04 | 2.49E-02 |
| g16887 | AAH49010 | 573095 | psmb3 | -0.55 | 6.61 | 21.66 | 3.18E-57 | 5.26E-04 | 2.50E-02 |
| g14751 | ACG75896 | 100526683 | aldh1l1 | -0.60 | 7.05 | 21.60 | 0.00E+00 | 5.38E-04 | 2.54E-02 |
| g2372 | XP_009295541 | 557738 | ccny | 0.59 | 4.74 | 21.55 | 1.92E-57 | 5.42E-04 | 2.54E-02 |
| g17560 | Q7SXK5 | 402826 | si:ch73-71d17.1 | 0.76 | 3.68 | 21.53 | 0.00E+00 | 5.51E-04 | 2.57E-02 |
| g17582 | NP_001314754 | 406280 | itih2 | 0.51 | 11.70 | 21.52 | 1.12E-103 | 5.66E-04 | 2.63E-02 |
| g14339 | CAD60658 | 368823 | fkbp11 | 0.55 | 7.85 | 21.51 | 0.00E+00 | 5.76E-04 | 2.66E-02 |
| g21532 | AAH46029 | 393187 | dmac2l | 0.84 | 4.12 | 21.48 | 0.00E+00 | 6.12E-04 | 2.81E-02 |
| g3305 | AAI50400 | 566527 | dad1 | -0.72 | 5.72 | 21.38 | 0.00E+00 | 6.16E-04 | 2.81E-02 |
| g25274 | XP_005157459 | 100126024 | zgc:174895 | -1.22 | 3.23 | 21.36 | 2.64E-64 | 6.19E-04 | 2.81E-02 |
| g23139 | XP_005162282 | 30634 | l1cama | 0.94 | 3.52 | 21.35 | 1.80E-02 | 6.34E-04 | 2.87E-02 |
| g11734 | P59723 | 373126 | hif1an | -1.15 | 2.88 | 21.20 | 0.00E+00 | 6.62E-04 | 2.98E-02 |
| g18435 | BAB90841 | 140634 | cyp1a | -0.84 | 3.98 | 21.20 | 0.00E+00 | 6.77E-04 | 3.03E-02 |
| g6089 | AAF66961 | 58122 | mcl1a | 0.47 | 8.16 | 21.11 | 0.00E+00 | 7.05E-04 | 3.14E-02 |
| g241 | AAH46072 | 266797 | stt3a | -0.63 | 5.36 | 21.09 | 1.01E-169 | 7.23E-04 | 3.21E-02 |
| g2158 | AAH64707 | 406314 | ubxn4 | -0.52 | 5.97 | 20.91 | 2.90E+00 | 7.49E-04 | 3.30E-02 |
| g21794 | BAD67593 | 335195 | ywhaqb | 0.48 | 7.18 | 20.81 | 2.33E-83 | 7.55E-04 | 3.31E-02 |
| g20239 | AAI09473 | 322228 | bhmt | 0.53 | 12.09 | 20.72 | 0.00E+00 | 7.81E-04 | 3.41E-02 |
| g13730 | XP_017207953 | 436657 | cpsf2 | -0.72 | 4.02 | 20.65 | 8.25E-84 | 7.92E-04 | 3.44E-02 |
| g12355 | AAF05817 | 30647 | psme2 | -0.64 | 5.50 | 20.58 | 4.07E-90 | 8.01E-04 | 3.46E-02 |
| g19766 | XP_002667717 | 100332841 | LOC100332841 | -1.10 | 2.85 | 20.55 | 0.00E+00 | 8.08E-04 | 3.47E-02 |
| g14573 | AAH54602 | 393902 | rab7a | -0.69 | 3.92 | 20.51 | 0.00E+00 | 8.14E-04 | 3.48E-02 |
| g5178 | NP_001093492 | 566469 | si:dkeyp-120h9.1 | -0.95 | 2.74 | 20.45 | 0.00E+00 | 8.22E-04 | 3.50E-02 |
| g17559 | NP_001038575 | 566596 | si:dkey-153k10.9 | 0.69 | 4.22 | 20.37 | 0.00E+00 | 8.40E-04 | 3.54E-02 |
| g8716 | XP_009292028 | 793125 | si:dkey-47k20.3 | -0.83 | 5.46 | 20.35 | 9.97E-118 | 8.44E-04 | 3.54E-02 |
| g9733 | XP_001924016 | 569097 | LOC569097 | 0.76 | 3.18 | 20.27 | 1.45E-178 | 8.44E-04 | 3.54E-02 |
| g1665 | AAH65443 | 322544 | dnajc3a | -0.75 | 4.19 | 20.26 | 0.00E+00 | 8.53E-04 | 3.55E-02 |
| g2495 | NP_001264046 | 560020 | acss2l | -0.73 | 4.36 | 20.25 | 3.53E-35 | 8.55E-04 | 3.55E-02 |
| g7751 | XP_021329551 | 797251 | si:ch211-236l14.4 | -0.68 | 3.97 | 20.23 | 0.00E+00 | 8.59E-04 | 3.55E-02 |
| g3159 | AAH45968 | 327057 | uck2b | 0.57 | 4.80 | 20.10 | 4.61E-60 | 8.93E-04 | 3.67E-02 |
| g21561 | A1L2F3 | 567446 | nusap1 | -0.91 | 3.58 | 19.99 | 0.00E+00 | 9.11E-04 | 3.73E-02 |
| g25858 | CAA74004 | 30355 | apoa1a | 0.64 | 12.31 | 19.87 | 0.00E+00 | 9.16E-04 | 3.73E-02 |
| g16399 | NP_001121843 | 100147856 | illr2 | 0.47 | 11.95 | 19.76 | 0.00E+00 | 9.19E-04 | 3.73E-02 |
| g20060 | XP_005166846 | 335159 | acsbg2 | -0.66 | 5.11 | 19.76 | 0.00E+00 | 9.50E-04 | 3.83E-02 |
| g23101 | AAY40842 | 565685 | fance | 0.69 | 4.97 | 19.77 | 0.00E+00 | 9.54E-04 | 3.83E-02 |
| g3582 | AAH83309 | 449786 | polr3glb | -1.07 | 2.24 | 19.64 | 5.97E-62 | 9.57E-04 | 3.83E-02 |
| g12596 | XP_005162731 | 322795 | csrnp1b | 0.50 | 6.69 | 19.64 | 7.65E-06 | 9.68E-04 | 3.85E-02 |
| g16398 | XP_021336864 | 100003029 | fgl1 | 0.48 | 12.35 | 19.50 | 2.09E-79 | 9.88E-04 | 3.92E-02 |
| g24477 | AAH68340 | 286746 | sdad1 | 0.47 | 6.94 | 19.46 | 0.00E+00 | 1.01E-03 | 3.97E-02 |
| g9261 | ABI33876 | 793274 | srebf1 | -1.21 | 2.31 | 19.37 | 9.17E-20 | 1.01E-03 | 3.97E-02 |
| g26494 | AAI15130 | 555526 | si:ch211-201h21.5 | 0.67 | 4.58 | 19.36 | 0.00E+00 | 1.03E-03 | 4.02E-02 |
| g3023 | NP_001243132 | 492647 | angptl4 | 0.56 | 6.53 | 19.17 | 7.01E-86 | 1.04E-03 | 4.05E-02 |
| g22852 | XP_009295191 | 564967 | zbtb17 | 0.77 | 3.37 | 19.01 | 0.00E+00 | 1.05E-03 | 4.05E-02 |
| g277 | AAI63387 | 566852 | pdzd3b | 0.69 | 4.25 | 18.99 | 0.00E+00 | 1.07E-03 | 4.10E-02 |
| g12186 | NP_956936 | 393615 | zgc:64201 | -1.32 | 2.36 | 18.79 | 0.00E+00 | 1.07E-03 | 4.10E-02 |
| g74 | AAX83257 | 378966 | ncf1 | 1.12 | 2.29 | 18.76 | 7.61E-33 | 1.08E-03 | 4.11E-02 |
| g20881 | NP_001116221 | 563963 | dgcr8 | -0.62 | 4.68 | 18.68 | 1.83E-61 | 1.09E-03 | 4.15E-02 |
| g17535 | AAH45345 | 322605 | chpt1 | 0.68 | 5.69 | 18.68 | 1.55E-124 | 1.10E-03 | 4.18E-02 |
| g12125 | AAH60926 | 393807 | ppp1r3cb | 0.82 | 3.36 | 18.68 | 0.00E+00 | 1.12E-03 | 4.22E-02 |
| g9725 | XP_009297793 | 327462 | hsd3b7 | -0.67 | 4.72 | 18.57 | 0.00E+00 | 1.24E-03 | 4.64E-02 |
| g12229 | AAH53263 | 406409 | cyb5a | 0.66 | 5.07 | 18.56 | 3.07E-67 | 1.24E-03 | 4.64E-02 |
| g4305 | AAH95058 | 554142 | rgrb | 0.75 | 3.82 | 18.55 | 1.18E-83 | 1.25E-03 | 4.64E-02 |
| g6850 | NP_001116330 | 560277 | masp2 | 0.47 | 11.22 | 18.54 | 3.28E-59 | 1.25E-03 | 4.64E-02 |
| g6322 | ABG35925 | 777792 | sult2st3 | -1.04 | 2.53 | 18.52 | 2.96E-10 | 1.26E-03 | 4.64E-02 |
| g15483 | XP_005173986 | 101882355 | LOC101882355 | -0.82 | 3.24 | 18.48 | 2.29E-123 | 1.26E-03 | 4.64E-02 |
| g8095 | AAH54696 | 337225 | chid1 | -0.92 | 2.76 | 18.47 | 0.00E+00 | 1.28E-03 | 4.69E-02 |
| g16704 | NP_001186962 | 794625 | atp5md | -0.67 | 6.64 | 18.40 | 8.56E-157 | 1.34E-03 | 4.87E-02 |
| g14438 | AAI42794 | 553214 | eif4ba | 0.43 | 7.42 | 18.39 | 0.00E+00 | 1.34E-03 | 4.87E-02 |
| g6507 | XP_009304581 | 558046 | trim2b | -1.09 | 2.27 | 18.31 | 0.00E+00 | 1.36E-03 | 4.90E-02 |

Supplementary Table 4: Gene Ontology (GO) annotation for up- and down-regulated pathways for the pituitary gland (*p*-value < 0.01). ID refers to the universal identifiers of the GO terms, the Ont column shows the ontology domain that each GO term belongs to. The three domains are: biological process (BP), cellular component (CC) and molecular function (MF). The N column represents the total number of genes annotated with each GO term. The Up and Down columns indicate the number of genes within the GO term that are significantly up- and down-regulated in high versus low treatment, respectively.

| ID | N | Up | Down | *p*- value Up | *p*-value Down | Ont | Term |
| --- | --- | --- | --- | --- | --- | --- | --- |
| GO:0060319 | 10 | 0 | 3 | 1.00 | 9.57E-05 | BP | primitive erythrocyte differentiation |
| GO:0002262 | 57 | 1 | 5 | 0.37 | 1.98E-04 | BP | myeloid cell homeostasis |
| GO:0005737 | 2845 | 20 | 43 | 0.78 | 2.19E-04 | CC | cytoplasm |
| GO:0048872 | 63 | 1 | 5 | 0.39 | 3.18E-04 | BP | homeostasis of number of cells |
| GO:0043295 | 4 | 0 | 2 | 1.00 | 5.33E-04 | MF | glutathione binding |
| GO:1900750 | 4 | 0 | 2 | 1.00 | 5.33E-04 | MF | oligopeptide binding |
| GO:0030218 | 51 | 0 | 4 | 1.00 | 1.37E-03 | BP | erythrocyte differentiation |
| GO:0034101 | 52 | 0 | 4 | 1.00 | 1.47E-03 | BP | erythrocyte homeostasis |
| GO:0060215 | 28 | 0 | 3 | 1.00 | 2.31E-03 | BP | primitive hemopoiesis |
| GO:0044444 | 1934 | 12 | 30 | 0.87 | 2.57E-03 | CC | cytoplasmic part |
| GO:0042246 | 64 | 1 | 4 | 0.40 | 3.17E-03 | BP | tissue regeneration |
| GO:0009311 | 10 | 0 | 2 | 1.00 | 3.85E-03 | BP | oligosaccharide metabolic process |
| GO:0006898 | 35 | 0 | 3 | 1.00 | 4.40E-03 | BP | receptor-mediated endocytosis |
| GO:0051084 | 11 | 0 | 2 | 1.00 | 4.68E-03 | BP | 'de novo' posttranslational protein folding |
| GO:0051085 | 11 | 0 | 2 | 1.00 | 4.68E-03 | BP | chaperone cofactor-dependent protein refolding |
| GO:0006458 | 12 | 0 | 2 | 1.00 | 5.58E-03 | BP | 'de novo' protein folding |
| GO:0004364 | 12 | 0 | 2 | 1.00 | 5.58E-03 | MF | glutathione transferase activity |
| GO:0035162 | 40 | 0 | 3 | 1.00 | 6.41E-03 | BP | embryonic hemopoiesis |
| GO:0044092 | 80 | 1 | 4 | 0.47 | 7.03E-03 | BP | negative regulation of molecular function |
| GO:0009611 | 127 | 2 | 5 | 0.27 | 7.17E-03 | BP | response to wounding |
| GO:0045765 | 42 | 0 | 3 | 1.00 | 7.35E-03 | BP | regulation of angiogenesis |
| GO:0070988 | 14 | 0 | 2 | 1.00 | 7.59E-03 | BP | demethylation |
| GO:0040007 | 185 | 3 | 6 | 0.18 | 8.30E-03 | BP | growth |
| GO:0061077 | 15 | 0 | 2 | 1.00 | 8.71E-03 | BP | chaperone-mediated protein folding |
| GO:0045766 | 15 | 0 | 2 | 1.00 | 8.71E-03 | BP | positive regulation of angiogenesis |
| GO:1904018 | 15 | 0 | 2 | 1.00 | 8.71E-03 | BP | positive regulation of vasculature development |
| GO:0030099 | 86 | 0 | 4 | 1.00 | 9.04E-03 | BP | myeloid cell differentiation |
| GO:0030729 | 1 | 0 | 1 | 1.00 | 9.54E-03 | MF | acetoacetate-CoA ligase activity |
| GO:0007195 | 1 | 0 | 1 | 1.00 | 9.54E-03 | BP | adenylate cyclase-inhibiting dopamine receptor signaling pathway |
| GO:0004557 | 1 | 0 | 1 | 1.00 | 9.54E-03 | MF | alpha-galactosidase activity |
| GO:1904158 | 1 | 0 | 1 | 1.00 | 9.54E-03 | BP | axonemal central apparatus assembly |
| GO:0004952 | 1 | 0 | 1 | 1.00 | 9.54E-03 | MF | dopamine neurotransmitter receptor activity |
| GO:0001591 | 1 | 0 | 1 | 1.00 | 9.54E-03 | MF | dopamine neurotransmitter receptor activity, coupled via Gi/Go |
| GO:0071603 | 1 | 0 | 1 | 1.00 | 9.54E-03 | BP | endothelial cell-cell adhesion |
| GO:0030228 | 1 | 0 | 1 | 1.00 | 9.54E-03 | MF | lipoprotein particle receptor activity |
| GO:0005041 | 1 | 0 | 1 | 1.00 | 9.54E-03 | MF | low-density lipoprotein particle receptor activity |
| GO:1990124 | 1 | 0 | 1 | 1.00 | 9.54E-03 | CC | messenger ribonucleoprotein complex |
| GO:1903966 | 1 | 0 | 1 | 1.00 | 9.54E-03 | BP | monounsaturated fatty acid biosynthetic process |
| GO:1903964 | 1 | 0 | 1 | 1.00 | 9.54E-03 | BP | monounsaturated fatty acid metabolic process |
| GO:1903170 | 1 | 0 | 1 | 1.00 | 9.54E-03 | BP | negative regulation of calcium ion transmembrane transport |
| GO:1901020 | 1 | 0 | 1 | 1.00 | 9.54E-03 | BP | negative regulation of calcium ion transmembrane transporter activity |
| GO:2001258 | 1 | 0 | 1 | 1.00 | 9.54E-03 | BP | negative regulation of cation channel activity |
| GO:2000766 | 1 | 0 | 1 | 1.00 | 9.54E-03 | BP | negative regulation of cytoplasmic translation |
| GO:1901386 | 1 | 0 | 1 | 1.00 | 9.54E-03 | BP | negative regulation of voltage-gated calcium channel activity |
| GO:0032896 | 1 | 0 | 1 | 1.00 | 9.54E-03 | MF | palmitoyl-CoA 9-desaturase activity |
| GO:0004615 | 1 | 0 | 1 | 1.00 | 9.54E-03 | MF | phosphomannomutase activity |
| GO:0090118 | 1 | 0 | 1 | 1.00 | 9.54E-03 | BP | receptor-mediated endocytosis involved in cholesterol transport |
| GO:0004768 | 1 | 0 | 1 | 1.00 | 9.54E-03 | MF | stearoyl-CoA 9-desaturase activity |
| GO:0045098 | 1 | 0 | 1 | 1.00 | 9.54E-03 | CC | type III intermediate filament |
| GO:0072341 | 16 | 0 | 2 | 1.00 | 9.89E-03 | MF | modified amino acid binding |
| GO:1901342 | 47 | 0 | 3 | 1.00 | 1.00E-02 | BP | regulation of vasculature development |
| GO:0042403 | 3 | 2 | 0 | 1.84E-04 | 1.00 | BP | thyroid hormone metabolic process |
| GO:0051129 | 68 | 5 | 0 | 1.89E-04 | 1.00 | BP | negative regulation of cellular component organization |
| GO:0008585 | 4 | 2 | 0 | 3.66E-04 | 1.00 | BP | female gonad development |
| GO:0032878 | 4 | 2 | 0 | 3.66E-04 | 1.00 | BP | regulation of establishment or maintenance of cell polarity |
| GO:0005179 | 21 | 3 | 1 | 5.69E-04 | 0.18 | MF | hormone activity |
| GO:0046545 | 5 | 2 | 0 | 6.07E-04 | 1.00 | BP | development of primary female sexual characteristics |
| GO:0046660 | 5 | 2 | 0 | 6.07E-04 | 1.00 | BP | female sex differentiation |
| GO:0031492 | 5 | 2 | 0 | 6.07E-04 | 1.00 | MF | nucleosomal DNA binding |
| GO:0016584 | 5 | 2 | 0 | 6.07E-04 | 1.00 | BP | nucleosome positioning |
| GO:0048232 | 24 | 3 | 0 | 8.51E-04 | 1.00 | BP | male gamete generation |
| GO:0007283 | 24 | 3 | 0 | 8.51E-04 | 1.00 | BP | spermatogenesis |
| GO:0031936 | 6 | 2 | 0 | 9.06E-04 | 1.00 | BP | negative regulation of chromatin silencing |
| GO:0060969 | 6 | 2 | 0 | 9.06E-04 | 1.00 | BP | negative regulation of gene silencing |
| GO:0009755 | 25 | 3 | 0 | 9.61E-04 | 1.00 | BP | hormone-mediated signaling pathway |
| GO:0008406 | 7 | 2 | 0 | 1.26E-03 | 1.00 | BP | gonad development |
| GO:0045815 | 7 | 2 | 0 | 1.26E-03 | 1.00 | BP | positive regulation of gene expression, epigenetic |
| GO:0007530 | 7 | 2 | 0 | 1.26E-03 | 1.00 | BP | sex determination |
| GO:0045137 | 8 | 2 | 0 | 1.67E-03 | 1.00 | BP | development of primary sexual characteristics |
| GO:0007064 | 8 | 2 | 0 | 1.67E-03 | 1.00 | BP | mitotic sister chromatid cohesion |
| GO:0045910 | 8 | 2 | 0 | 1.67E-03 | 1.00 | BP | negative regulation of DNA recombination |
| GO:0031935 | 8 | 2 | 0 | 1.67E-03 | 1.00 | BP | regulation of chromatin silencing |
| GO:0018958 | 9 | 2 | 0 | 2.14E-03 | 1.00 | BP | phenol-containing compound metabolic process |
| GO:0048608 | 9 | 2 | 0 | 2.14E-03 | 1.00 | BP | reproductive structure development |
| GO:0061458 | 9 | 2 | 0 | 2.14E-03 | 1.00 | BP | reproductive structure development |
| GO:0007548 | 9 | 2 | 0 | 2.14E-03 | 1.00 | BP | sex differentiation |
| GO:0003006 | 35 | 3 | 0 | 2.59E-03 | 1.00 | BP | developmental process involved in reproduction |
| GO:0048018 | 74 | 4 | 3 | 2.72E-03 | 0.03 | MF | receptor ligand activity |
| GO:0030545 | 76 | 4 | 3 | 3.00E-03 | 0.04 | MF | receptor regulator activity |
| GO:1905268 | 11 | 2 | 0 | 3.24E-03 | 1.00 | BP | negative regulation of chromatin organization |
| GO:0040029 | 38 | 3 | 0 | 3.28E-03 | 1.00 | BP | regulation of gene expression, epigenetic |
| GO:0030261 | 12 | 2 | 0 | 3.87E-03 | 1.00 | BP | chromosome condensation |
| GO:0000018 | 12 | 2 | 0 | 3.87E-03 | 1.00 | BP | regulation of DNA recombination |
| GO:0060968 | 12 | 2 | 0 | 3.87E-03 | 1.00 | BP | regulation of gene silencing |
| GO:0051704 | 135 | 5 | 1 | 4.19E-03 | 0.73 | BP | multi-organism process |
| GO:0008630 | 13 | 2 | 0 | 4.55E-03 | 1.00 | BP | intrinsic apoptotic signaling pathway in response to DNA damage |
| GO:0007276 | 44 | 3 | 0 | 4.97E-03 | 1.00 | BP | gamete generation |
| GO:0016458 | 44 | 3 | 0 | 4.97E-03 | 1.00 | BP | gene silencing |
| GO:0048609 | 44 | 3 | 0 | 4.97E-03 | 1.00 | BP | multicellular organismal reproductive process |
| GO:0005576 | 339 | 8 | 6 | 5.05E-03 | 0.10 | CC | extracellular region |
| GO:0032504 | 45 | 3 | 0 | 5.30E-03 | 1.00 | BP | multicellular organism reproduction |
| GO:0010639 | 46 | 3 | 0 | 5.64E-03 | 1.00 | BP | negative regulation of organelle organization |
| GO:0140014 | 47 | 3 | 0 | 5.99E-03 | 1.00 | BP | mitotic nuclear division |
| GO:0005102 | 213 | 6 | 3 | 6.58E-03 | 0.33 | MF | signaling receptor binding |
| GO:0031491 | 16 | 2 | 0 | 6.89E-03 | 1.00 | MF | nucleosome binding |
| GO:0031490 | 17 | 2 | 1 | 7.77E-03 | 0.15 | MF | chromatin DNA binding |
| GO:0000786 | 17 | 2 | 0 | 7.77E-03 | 1.00 | CC | nucleosome |
| GO:0007062 | 17 | 2 | 0 | 7.77E-03 | 1.00 | BP | sister chromatid cohesion |
| GO:0003860 | 1 | 1 | 0 | 7.91E-03 | 1.00 | MF | 3-hydroxyisobutyryl-CoA hydrolase activity |
| GO:0003877 | 1 | 1 | 0 | 7.91E-03 | 1.00 | MF | ATP adenylyltransferase activity |
| GO:0007414 | 1 | 1 | 0 | 7.91E-03 | 1.00 | BP | axonal defasciculation |
| GO:0045575 | 1 | 1 | 0 | 7.91E-03 | 1.00 | BP | basophil activation |
| GO:0002276 | 1 | 1 | 0 | 7.91E-03 | 1.00 | BP | basophil activation involved in immune response |
| GO:0005432 | 1 | 1 | 0 | 7.91E-03 | 1.00 | MF | calcium:sodium antiporter activity |
| GO:0071481 | 1 | 1 | 0 | 7.91E-03 | 1.00 | BP | cellular response to X-ray |
| GO:0015960 | 1 | 1 | 0 | 7.91E-03 | 1.00 | BP | diadenosine polyphosphate biosynthetic process |
| GO:0015966 | 1 | 1 | 0 | 7.91E-03 | 1.00 | BP | diadenosine tetraphosphate biosynthetic process |
| GO:0015965 | 1 | 1 | 0 | 7.91E-03 | 1.00 | BP | diadenosine tetraphosphate metabolic process |
| GO:0003347 | 1 | 1 | 0 | 7.91E-03 | 1.00 | BP | epicardial cell to mesenchymal cell transition |
| GO:0070287 | 1 | 1 | 0 | 7.91E-03 | 1.00 | MF | ferritin receptor activity |
| GO:0016913 | 1 | 1 | 0 | 7.91E-03 | 1.00 | MF | follicle-stimulating hormone activity |
| GO:0016914 | 1 | 1 | 0 | 7.91E-03 | 1.00 | CC | follicle-stimulating hormone complex |
| GO:0036230 | 1 | 1 | 0 | 7.91E-03 | 1.00 | BP | granulocyte activation |
| GO:0043564 | 1 | 1 | 0 | 7.91E-03 | 1.00 | CC | Ku70:Ku80 complex |
| GO:0004824 | 1 | 1 | 0 | 7.91E-03 | 1.00 | MF | lysine-tRNA ligase activity |
| GO:0006430 | 1 | 1 | 0 | 7.91E-03 | 1.00 | BP | lysyl-tRNA aminoacylation |
| GO:0042116 | 1 | 1 | 0 | 7.91E-03 | 1.00 | BP | macrophage activation |
| GO:0048496 | 1 | 1 | 0 | 7.91E-03 | 1.00 | BP | maintenance of animal organ identity |
| GO:1903392 | 1 | 1 | 0 | 7.91E-03 | 1.00 | BP | negative regulation of adherens junction organization |
| GO:0001607 | 1 | 1 | 0 | 7.91E-03 | 1.00 | MF | neuromedin U receptor activity |
| GO:0030728 | 1 | 1 | 0 | 7.91E-03 | 1.00 | BP | ovulation |
| GO:0061696 | 1 | 1 | 0 | 7.91E-03 | 1.00 | CC | pituitary gonadotropin complex |
| GO:0043032 | 1 | 1 | 0 | 7.91E-03 | 1.00 | BP | positive regulation of macrophage activation |
| GO:0010893 | 1 | 1 | 0 | 7.91E-03 | 1.00 | BP | positive regulation of steroid biosynthetic process |
| GO:0043030 | 1 | 1 | 0 | 7.91E-03 | 1.00 | BP | regulation of macrophage activation |
| GO:0050810 | 1 | 1 | 0 | 7.91E-03 | 1.00 | BP | regulation of steroid biosynthetic process |
| GO:0040040 | 1 | 1 | 0 | 7.91E-03 | 1.00 | BP | thermosensory behavior |
| GO:0006590 | 1 | 1 | 0 | 7.91E-03 | 1.00 | BP | thyroid hormone generation |
| GO:0102524 | 1 | 1 | 0 | 7.91E-03 | 1.00 | MF | tRNAPhe (7-(3-amino-3-carboxypropyl)wyosine37-C2)-hydroxylase activity |
| GO:0031591 | 1 | 1 | 0 | 7.91E-03 | 1.00 | BP | wybutosine biosynthetic process |
| GO:0031590 | 1 | 1 | 0 | 7.91E-03 | 1.00 | BP | wybutosine metabolic process |
| GO:0043066 | 52 | 3 | 0 | 7.94E-03 | 1.00 | BP | negative regulation of apoptotic process |
| GO:0019953 | 52 | 3 | 0 | 7.94E-03 | 1.00 | BP | sexual reproduction |
| GO:0044703 | 53 | 3 | 0 | 8.36E-03 | 1.00 | BP | multi-organism reproductive process |
| GO:0043069 | 53 | 3 | 0 | 8.36E-03 | 1.00 | BP | negative regulation of programmed cell death |
| GO:0003684 | 18 | 2 | 0 | 8.69E-03 | 1.00 | MF | damaged DNA binding |
| GO:0000049 | 18 | 2 | 0 | 8.69E-03 | 1.00 | MF | tRNA binding |
| GO:0005615 | 228 | 6 | 4 | 9.07E-03 | 0.17 | CC | extracellular space |
| GO:0006259 | 229 | 6 | 1 | 9.26E-03 | 0.89 | BP | DNA metabolic process |
| GO:0044815 | 19 | 2 | 0 | 9.67E-03 | 1.00 | CC | DNA packaging complex |
| GO:0051053 | 19 | 2 | 0 | 9.67E-03 | 1.00 | BP | negative regulation of DNA metabolic process |
| GO:0006955 | 107 | 4 | 1 | 1.00E-02 | 0.64 | BP | immune response |

Supplementary Table 5: Gene Ontology (GO) annotation for up- and down-regulated pathways for liver (*p*-value < 0.01). ID refers to the universal identifiers of the GO terms, the Ont column shows the ontology domain that each GO term belongs to. The three domains are: biological process (BP), cellular component (CC) and molecular function (MF). The N column represents the total number of genes annotated with each GO term. The Up and Down columns indicate the number of genes within the GO term that are significantly up- and down-regulated in high versus low treatment, respectively.

| ID | N | Up | Down | *p*- value Up | *p*-value Down | Ont | Term |
| --- | --- | --- | --- | --- | --- | --- | --- |
| GO:1905369 | 42 | 1 | 24 | 0.90 | 1.37E-22 | CC | endopeptidase complex |
| GO:0000502 | 42 | 1 | 24 | 0.90 | 1.37E-22 | CC | proteasome complex |
| GO:1905368 | 54 | 1 | 24 | 0.95 | 3.38E-19 | CC | peptidase complex |
| GO:0022624 | 21 | 0 | 14 | 1.00 | 5.21E-15 | CC | proteasome accessory complex |
| GO:0005838 | 18 | 0 | 13 | 1.00 | 9.99E-15 | CC | proteasome regulatory particle |
| GO:1901565 | 362 | 23 | 48 | 0.19 | 1.59E-12 | BP | organonitrogen compound catabolic process |
| GO:0030163 | 255 | 10 | 38 | 0.86 | 1.18E-11 | BP | protein catabolic process |
| GO:1901575 | 566 | 39 | 60 | 0.04 | 3.59E-11 | BP | organic substance catabolic process |
| GO:0009056 | 657 | 45 | 65 | 0.04 | 9.96E-11 | BP | catabolic process |
| GO:0043632 | 211 | 6 | 32 | 0.97 | 3.46E-10 | BP | modification-dependent macromolecule catabolic process |
| GO:0044248 | 592 | 43 | 59 | 0.02 | 6.39E-10 | BP | cellular catabolic process |
| GO:0005839 | 14 | 0 | 9 | 1.00 | 7.62E-10 | CC | proteasome core complex |
| GO:0019941 | 206 | 6 | 31 | 0.96 | 8.05E-10 | BP | modification-dependent protein catabolic process |
| GO:0051603 | 232 | 9 | 33 | 0.86 | 1.00E-09 | BP | proteolysis involved in cellular protein catabolic process |
| GO:0044257 | 234 | 10 | 33 | 0.79 | 1.25E-09 | BP | cellular protein catabolic process |
| GO:0006511 | 200 | 4 | 30 | 0.99 | 1.66E-09 | BP | ubiquitin-dependent protein catabolic process |
| GO:0010499 | 15 | 1 | 9 | 0.55 | 1.83E-09 | BP | proteasomal ubiquitin-independent protein catabolic process |
| GO:0004298 | 15 | 0 | 9 | 1.00 | 1.83E-09 | MF | threonine-type endopeptidase activity |
| GO:0070003 | 15 | 0 | 9 | 1.00 | 1.83E-09 | MF | threonine-type peptidase activity |
| GO:0003824 | 2768 | 133 | 171 | 0.90 | 6.45E-09 | MF | catalytic activity |
| GO:0008540 | 9 | 0 | 7 | 1.00 | 8.65E-09 | CC | proteasome regulatory particle, base subcomplex |
| GO:0009057 | 347 | 14 | 40 | 0.88 | 8.90E-09 | BP | macromolecule catabolic process |
| GO:0005783 | 336 | 8 | 39 | 1.00 | 1.14E-08 | CC | endoplasmic reticulum |
| GO:0008541 | 7 | 0 | 6 | 1.00 | 4.13E-08 | CC | proteasome regulatory particle, lid subcomplex |
| GO:0010498 | 125 | 1 | 21 | 1.00 | 6.82E-08 | BP | proteasomal protein catabolic process |
| GO:0043161 | 116 | 0 | 20 | 1.00 | 9.02E-08 | BP | proteasome-mediated ubiquitin-dependent protein catabolic process |
| GO:0004576 | 8 | 0 | 6 | 1.00 | 1.59E-07 | MF | oligosaccharyl transferase activity |
| GO:0044265 | 313 | 13 | 34 | 0.84 | 5.18E-07 | BP | cellular macromolecule catabolic process |
| GO:0005737 | 2845 | 161 | 167 | 0.11 | 5.68E-07 | CC | cytoplasm |
| GO:0004579 | 6 | 0 | 5 | 1.00 | 8.33E-07 | MF | dolichyl-diphosphooligosaccharide-protein glycotransferase activity |
| GO:2000144 | 6 | 0 | 5 | 1.00 | 8.33E-07 | BP | positive regulation of DNA-templated transcription, initiation |
| GO:0060261 | 6 | 0 | 5 | 1.00 | 8.33E-07 | BP | positive regulation of transcription initiation from RNA polymerase II promoter |
| GO:0036402 | 6 | 0 | 5 | 1.00 | 8.33E-07 | MF | proteasome-activating ATPase activity |
| GO:2000142 | 6 | 0 | 5 | 1.00 | 8.33E-07 | BP | regulation of DNA-templated transcription, initiation |
| GO:0060260 | 6 | 0 | 5 | 1.00 | 8.33E-07 | BP | regulation of transcription initiation from RNA polymerase II promoter |
| GO:0032787 | 114 | 11 | 18 | 0.04 | 1.54E-06 | BP | monocarboxylic acid metabolic process |
| GO:0006508 | 458 | 30 | 42 | 0.12 | 2.29E-06 | BP | proteolysis |
| GO:0016491 | 374 | 35 | 36 | 0.00 | 4.26E-06 | MF | oxidoreductase activity |
| GO:0044444 | 1934 | 115 | 120 | 0.06 | 4.56E-06 | CC | cytoplasmic part |
| GO:0055114 | 353 | 30 | 34 | 0.01 | 7.87E-06 | BP | oxidation-reduction process |
| GO:0045899 | 5 | 0 | 4 | 1.00 | 1.63E-05 | BP | positive regulation of RNA polymerase II transcriptional preinitiation complex assembly |
| GO:0045898 | 5 | 0 | 4 | 1.00 | 1.63E-05 | BP | regulation of RNA polymerase II transcriptional preinitiation complex assembly |
| GO:0006631 | 67 | 7 | 12 | 0.06 | 2.38E-05 | BP | fatty acid metabolic process |
| GO:0006082 | 297 | 26 | 29 | 0.01 | 2.91E-05 | BP | organic acid metabolic process |
| GO:0008610 | 116 | 10 | 16 | 0.08 | 3.39E-05 | BP | lipid biosynthetic process |
| GO:0008250 | 6 | 0 | 4 | 1.00 | 4.73E-05 | CC | oligosaccharyltransferase complex |
| GO:0019773 | 6 | 0 | 4 | 1.00 | 4.73E-05 | CC | proteasome core complex, alpha-subunit complex |
| GO:0005215 | 387 | 14 | 34 | 0.95 | 5.40E-05 | MF | transporter activity |
| GO:0012505 | 733 | 19 | 54 | 1.00 | 5.42E-05 | CC | endomembrane system |
| GO:0019752 | 278 | 19 | 27 | 0.14 | 6.04E-05 | BP | carboxylic acid metabolic process |
| GO:0015711 | 75 | 4 | 12 | 0.56 | 7.59E-05 | BP | organic anion transport |
| GO:0006629 | 282 | 21 | 27 | 0.06 | 7.72E-05 | BP | lipid metabolic process |
| GO:0004364 | 12 | 1 | 5 | 0.47 | 8.87E-05 | MF | glutathione transferase activity |
| GO:0043436 | 286 | 19 | 27 | 0.17 | 9.82E-05 | BP | oxoacid metabolic process |
| GO:0006633 | 27 | 3 | 7 | 0.16 | 1.09E-04 | BP | fatty acid biosynthetic process |
| GO:0044432 | 212 | 3 | 22 | 1.00 | 1.12E-04 | CC | endoplasmic reticulum part |
| GO:0046942 | 47 | 2 | 9 | 0.71 | 1.47E-04 | BP | carboxylic acid transport |
| GO:0015849 | 48 | 2 | 9 | 0.72 | 1.74E-04 | BP | organic acid transport |
| GO:0016209 | 29 | 1 | 7 | 0.79 | 1.77E-04 | MF | antioxidant activity |
| GO:0018196 | 8 | 0 | 4 | 1.00 | 2.06E-04 | BP | peptidyl-asparagine modification |
| GO:0042623 | 164 | 3 | 18 | 0.99 | 2.33E-04 | MF | ATPase activity, coupled |
| GO:0003674 | 6511 | 369 | 308 | 0.00 | 2.33E-04 | MF | molecular_function |
| GO:1902494 | 560 | 14 | 42 | 1.00 | 2.69E-04 | CC | catalytic complex |
| GO:0006820 | 111 | 4 | 14 | 0.84 | 2.75E-04 | BP | anion transport |
| GO:0055085 | 324 | 10 | 28 | 0.98 | 3.27E-04 | BP | transmembrane transport |
| GO:0022857 | 343 | 12 | 29 | 0.95 | 3.70E-04 | MF | transmembrane transporter activity |
| GO:0042737 | 43 | 9 | 8 | 0.00 | 4.21E-04 | BP | drug catabolic process |
| GO:0005789 | 189 | 3 | 19 | 1.00 | 4.87E-04 | CC | endoplasmic reticulum membrane |
| GO:0043231 | 2943 | 144 | 157 | 0.85 | 4.93E-04 | CC | intracellular membrane-bounded organelle |
| GO:0098827 | 190 | 3 | 19 | 1.00 | 5.20E-04 | CC | endoplasmic reticulum subcompartment |
| GO:0005622 | 4340 | 234 | 218 | 0.25 | 5.37E-04 | CC | intracellular |
| GO:0044424 | 4340 | 234 | 218 | 0.25 | 5.37E-04 | CC | intracellular part |
| GO:0006694 | 25 | 3 | 6 | 0.14 | 5.39E-04 | BP | steroid biosynthetic process |
| GO:0042175 | 191 | 3 | 19 | 1.00 | 5.55E-04 | CC | nuclear outer membrane-endoplasmic reticulum membrane network |
| GO:0043248 | 10 | 0 | 4 | 1.00 | 5.77E-04 | BP | proteasome assembly |
| GO:0042626 | 57 | 0 | 9 | 1.00 | 6.62E-04 | MF | ATPase activity, coupled to transmembrane movement of substances |
| GO:0046943 | 36 | 1 | 7 | 0.86 | 7.32E-04 | MF | carboxylic acid transmembrane transporter activity |
| GO:0031597 | 5 | 0 | 3 | 1.00 | 7.41E-04 | CC | cytosolic proteasome complex |
| GO:0033764 | 5 | 1 | 3 | 0.24 | 7.41E-04 | MF | steroid dehydrogenase activity, acting on the CH-OH group of donors, NAD or NADP as acceptor |
| GO:0006811 | 344 | 11 | 28 | 0.97 | 8.50E-04 | BP | ion transport |
| GO:0098656 | 37 | 0 | 7 | 1.00 | 8.70E-04 | BP | anion transmembrane transport |
| GO:0005342 | 37 | 1 | 7 | 0.86 | 8.70E-04 | MF | organic acid transmembrane transporter activity |
| GO:0006636 | 11 | 1 | 4 | 0.45 | 8.76E-04 | BP | unsaturated fatty acid biosynthetic process |
| GO:0044255 | 215 | 19 | 20 | 0.02 | 9.40E-04 | BP | cellular lipid metabolic process |
| GO:0006457 | 72 | 2 | 10 | 0.90 | 9.54E-04 | BP | protein folding |
| GO:0015405 | 60 | 0 | 9 | 1.00 | 9.71E-04 | MF | P-P-bond-hydrolysis-driven transmembrane transporter activity |
| GO:1905039 | 19 | 0 | 5 | 1.00 | 1.01E-03 | BP | carboxylic acid transmembrane transport |
| GO:0022626 | 71 | 31 | 1 | 1.20E-21 | 0.96 | CC | cytosolic ribosome |
| GO:0003735 | 123 | 36 | 0 | 4.16E-18 | 1.00 | MF | structural constituent of ribosome |
| GO:0022625 | 39 | 21 | 0 | 1.97E-17 | 1.00 | CC | cytosolic large ribosomal subunit |
| GO:0005840 | 145 | 37 | 1 | 2.09E-16 | 1.00 | CC | ribosome |
| GO:0044445 | 108 | 32 | 4 | 2.16E-16 | 0.69 | CC | cytosolic part |
| GO:0044391 | 119 | 32 | 1 | 4.76E-15 | 0.99 | CC | ribosomal subunit |
| GO:0005198 | 252 | 42 | 9 | 1.25E-11 | 0.76 | MF | structural molecule activity |
| GO:0015934 | 70 | 21 | 0 | 2.68E-11 | 1.00 | CC | large ribosomal subunit |
| GO:0005615 | 228 | 38 | 7 | 1.25E-10 | 0.87 | CC | extracellular space |
| GO:0005576 | 339 | 48 | 13 | 1.58E-10 | 0.71 | CC | extracellular region |
| GO:0006412 | 286 | 43 | 6 | 2.23E-10 | 0.99 | BP | translation |
| GO:0043043 | 289 | 43 | 6 | 3.13E-10 | 0.99 | BP | peptide biosynthetic process |
| GO:0005829 | 468 | 58 | 30 | 3.82E-10 | 0.02 | CC | cytosol |
| GO:0072376 | 20 | 11 | 1 | 7.67E-10 | 0.59 | BP | protein activation cascade |
| GO:0043604 | 320 | 44 | 12 | 2.49E-09 | 0.73 | BP | amide biosynthetic process |
| GO:0006518 | 335 | 45 | 9 | 3.43E-09 | 0.96 | BP | peptide metabolic process |
| GO:0042273 | 46 | 15 | 0 | 5.34E-09 | 1.00 | BP | ribosomal large subunit biogenesis |
| GO:0044421 | 260 | 38 | 8 | 5.94E-09 | 0.88 | CC | extracellular region part |
| GO:0043603 | 396 | 49 | 17 | 1.06E-08 | 0.54 | BP | cellular amide metabolic process |
| GO:1990904 | 390 | 48 | 11 | 1.81E-08 | 0.95 | CC | ribonucleoprotein complex |
| GO:0006956 | 14 | 8 | 1 | 1.19E-07 | 0.46 | BP | complement activation |
| GO:0046503 | 15 | 8 | 2 | 2.42E-07 | 0.13 | BP | glycerolipid catabolic process |
| GO:1901566 | 597 | 60 | 26 | 4.90E-07 | 0.51 | BP | organonitrogen compound biosynthetic process |
| GO:0005506 | 57 | 14 | 8 | 8.93E-07 | 0.00 | MF | iron ion binding |
| GO:0022627 | 30 | 10 | 1 | 1.60E-06 | 0.73 | CC | cytosolic small ribosomal subunit |
| GO:0006959 | 20 | 8 | 1 | 3.76E-06 | 0.59 | BP | humoral immune response |
| GO:0042254 | 153 | 23 | 2 | 3.98E-06 | 0.99 | BP | ribosome biogenesis |
| GO:0000027 | 16 | 7 | 0 | 7.68E-06 | 1.00 | BP | ribosomal large subunit assembly |
| GO:0002252 | 43 | 11 | 2 | 8.78E-06 | 0.56 | BP | immune effector process |
| GO:0004497 | 37 | 10 | 4 | 1.33E-05 | 0.07 | MF | monooxygenase activity |
| GO:0046464 | 12 | 6 | 2 | 1.39E-05 | 0.09 | BP | acylglycerol catabolic process |
| GO:0046461 | 12 | 6 | 2 | 1.39E-05 | 0.09 | BP | neutral lipid catabolic process |
| GO:1901576 | 1553 | 116 | 62 | 1.63E-05 | 0.77 | BP | organic substance biosynthetic process |
| GO:0042738 | 8 | 5 | 2 | 1.87E-05 | 0.04 | BP | exogenous drug catabolic process |
| GO:0008395 | 8 | 5 | 2 | 1.87E-05 | 0.04 | MF | steroid hydroxylase activity |
| GO:0019433 | 8 | 5 | 2 | 1.87E-05 | 0.04 | BP | triglyceride catabolic process |
| GO:0044249 | 1512 | 113 | 58 | 2.14E-05 | 0.86 | BP | cellular biosynthetic process |
| GO:0009058 | 1580 | 117 | 66 | 2.22E-05 | 0.63 | BP | biosynthetic process |
| GO:0006639 | 25 | 8 | 3 | 2.56E-05 | 0.09 | BP | acylglycerol metabolic process |
| GO:0006638 | 25 | 8 | 3 | 2.56E-05 | 0.09 | BP | neutral lipid metabolic process |
| GO:0044271 | 1239 | 96 | 42 | 2.64E-05 | 0.97 | BP | cellular nitrogen compound biosynthetic process |
| GO:0008152 | 3717 | 236 | 189 | 2.84E-05 | 0.00 | BP | metabolic process |
| GO:0006641 | 19 | 7 | 3 | 2.95E-05 | 0.05 | BP | triglyceride metabolic process |
| GO:0015935 | 49 | 11 | 1 | 3.35E-05 | 0.88 | CC | small ribosomal subunit |
| GO:0006955 | 107 | 17 | 3 | 3.52E-05 | 0.85 | BP | immune response |
| GO:0061134 | 58 | 12 | 4 | 3.53E-05 | 0.24 | MF | peptidase regulator activity |
| GO:0045834 | 9 | 5 | 2 | 4.02E-05 | 0.05 | BP | positive regulation of lipid metabolic process |
| GO:0022613 | 230 | 27 | 4 | 6.22E-05 | 0.99 | BP | ribonucleoprotein complex biogenesis |
| GO:0007596 | 28 | 8 | 0 | 6.41E-05 | 1.00 | BP | blood coagulation |
| GO:0006144 | 15 | 6 | 3 | 6.56E-05 | 0.02 | BP | purine nucleobase metabolic process |
| GO:0042255 | 36 | 9 | 0 | 7.10E-05 | 1.00 | BP | ribosome assembly |
| GO:0071704 | 3465 | 220 | 172 | 7.95E-05 | 0.01 | BP | organic substance metabolic process |
| GO:0002181 | 45 | 10 | 2 | 8.37E-05 | 0.58 | BP | cytoplasmic translation |
| GO:0007599 | 29 | 8 | 0 | 8.45E-05 | 1.00 | BP | hemostasis |
| GO:0050878 | 29 | 8 | 0 | 8.45E-05 | 1.00 | BP | regulation of body fluid levels |
| GO:1901564 | 2013 | 139 | 110 | 1.03E-04 | 0.00 | BP | organonitrogen compound metabolic process |
| GO:0050817 | 30 | 8 | 0 | 1.10E-04 | 1.00 | BP | coagulation |
| GO:0070887 | 360 | 36 | 17 | 1.22E-04 | 0.38 | BP | cellular response to chemical stimulus |
| GO:0020037 | 47 | 10 | 4 | 1.24E-04 | 0.14 | MF | heme binding |
| GO:0016712 | 11 | 5 | 3 | 1.35E-04 | 0.01 | MF | oxidoreductase activity, acting on paired donors, with incorporation or reduction of molecular oxygen, reduced flavin or flavoprotein as one donor, and incorporation of one atom of oxygen |
| GO:0005577 | 3 | 3 | 0 | 1.42E-04 | 1.00 | CC | fibrinogen complex |
| GO:1901998 | 3 | 3 | 0 | 1.42E-04 | 1.00 | BP | toxin transport |
| GO:0005575 | 6350 | 364 | 292 | 1.50E-04 | 0.01 | CC | cellular_component |
| GO:0009112 | 24 | 7 | 4 | 1.61E-04 | 0.02 | BP | nucleobase metabolic process |
| GO:0050778 | 40 | 9 | 1 | 1.71E-04 | 0.83 | BP | positive regulation of immune response |
| GO:1901652 | 40 | 9 | 1 | 1.71E-04 | 0.83 | BP | response to peptide |
| GO:0043434 | 40 | 9 | 1 | 1.71E-04 | 0.83 | BP | response to peptide hormone |
| GO:0004866 | 49 | 10 | 2 | 1.79E-04 | 0.63 | MF | endopeptidase inhibitor activity |
| GO:0046113 | 7 | 4 | 2 | 2.27E-04 | 0.03 | BP | nucleobase catabolic process |
| GO:0004252 | 60 | 11 | 0 | 2.34E-04 | 1.00 | MF | serine-type endopeptidase activity |
| GO:0034645 | 1213 | 90 | 39 | 2.45E-04 | 0.98 | BP | cellular macromolecule biosynthetic process |
| GO:0044242 | 51 | 10 | 6 | 2.53E-04 | 0.02 | BP | cellular lipid catabolic process |
| GO:0030414 | 51 | 10 | 2 | 2.53E-04 | 0.65 | MF | peptidase inhibitor activity |
| GO:0050776 | 51 | 10 | 1 | 2.53E-04 | 0.89 | BP | regulation of immune response |
| GO:0046906 | 51 | 10 | 4 | 2.53E-04 | 0.18 | MF | tetrapyrrole binding |
| GO:0009059 | 1218 | 90 | 39 | 2.83E-04 | 0.99 | BP | macromolecule biosynthetic process |
| GO:0051346 | 34 | 8 | 1 | 2.84E-04 | 0.78 | BP | negative regulation of hydrolase activity |
| GO:0002682 | 104 | 15 | 3 | 3.04E-04 | 0.83 | BP | regulation of immune system process |
| GO:0042737 | 43 | 9 | 8 | 3.07E-04 | 0.00 | BP | drug catabolic process |
| GO:0030073 | 13 | 5 | 0 | 3.45E-04 | 1.00 | BP | insulin secretion |
| GO:0046883 | 13 | 5 | 0 | 3.45E-04 | 1.00 | BP | regulation of hormone secretion |
| GO:0019843 | 27 | 7 | 0 | 3.61E-04 | 1.00 | MF | rRNA binding |
| GO:0061135 | 54 | 10 | 4 | 4.11E-04 | 0.20 | MF | endopeptidase regulator activity |
| GO:0002253 | 36 | 8 | 1 | 4.31E-04 | 0.80 | BP | activation of immune response |
| GO:0034641 | 1917 | 130 | 70 | 4.33E-04 | 0.95 | BP | cellular nitrogen compound metabolic process |
| GO:0003674 | 6511 | 369 | 308 | 4.71E-04 | 0.00 | MF | molecular_function |
| GO:0043232 | 1023 | 77 | 36 | 4.88E-04 | 0.92 | CC | intracellular non-membrane-bounded organelle |
| GO:0043228 | 1023 | 77 | 36 | 4.88E-04 | 0.92 | CC | non-membrane-bounded organelle |
| GO:0030072 | 14 | 5 | 0 | 5.14E-04 | 1.00 | BP | peptide hormone secretion |
| GO:0016491 | 374 | 35 | 36 | 5.37E-04 | 0.00 | MF | oxidoreductase activity |
| GO:0010951 | 21 | 6 | 1 | 5.44E-04 | 0.60 | BP | negative regulation of endopeptidase activity |
| GO:0016725 | 4 | 3 | 0 | 5.44E-04 | 1.00 | MF | oxidoreductase activity, acting on CH or CH2 groups |
| GO:0006145 | 4 | 3 | 1 | 5.44E-04 | 0.16 | BP | purine nucleobase catabolic process |
| GO:0048584 | 209 | 23 | 3 | 5.52E-04 | 0.99 | BP | positive regulation of response to stimulus |
| GO:0009410 | 38 | 8 | 3 | 6.36E-04 | 0.22 | BP | response to xenobiotic stimulus |
| GO:0002684 | 57 | 10 | 2 | 6.45E-04 | 0.71 | BP | positive regulation of immune system process |
| GO:0072523 | 15 | 5 | 1 | 7.38E-04 | 0.48 | BP | purine-containing compound catabolic process |
| GO:0006805 | 15 | 5 | 2 | 7.38E-04 | 0.13 | BP | xenobiotic metabolic process |
| GO:0006577 | 9 | 4 | 0 | 7.50E-04 | 1.00 | BP | amino-acid betaine metabolic process |
| GO:0050796 | 9 | 4 | 0 | 7.50E-04 | 1.00 | BP | regulation of insulin secretion |
| GO:0052548 | 39 | 8 | 2 | 7.64E-04 | 0.51 | BP | regulation of endopeptidase activity |
| GO:0044238 | 3305 | 205 | 158 | 8.17E-04 | 0.05 | BP | primary metabolic process |
| GO:0006807 | 3120 | 195 | 143 | 8.25E-04 | 0.18 | BP | nitrogen compound metabolic process |
| GO:0048518 | 816 | 63 | 26 | 8.90E-04 | 0.96 | BP | positive regulation of biological process |
| GO:0017171 | 70 | 11 | 0 | 9.23E-04 | 1.00 | MF | serine hydrolase activity |
| GO:0008236 | 70 | 11 | 0 | 9.23E-04 | 1.00 | MF | serine-type peptidase activity |
| GO:0010466 | 23 | 6 | 1 | 9.26E-04 | 0.64 | BP | negative regulation of peptidase activity |
| GO:0004867 | 23 | 6 | 2 | 9.26E-04 | 0.26 | MF | serine-type endopeptidase inhibitor activity |
| GO:0046879 | 16 | 5 | 0 | 1.03E-03 | 1.00 | BP | hormone secretion |

Supplementary Table 6: List of KEGG pathways for up- and down-regulated pathways for the pituitary gland. ID refers to the universal identifiers of the KEGG terms, the N column represents the total number of genes annotated with each KEGG pathway. The Up and Down columns indicate the number of genes within the KEGG pathway that are significantly up- and down-regulated in high versus low treatment, respectively

| ID | ko | N | Up | Down | *p*- value Up | *p*-value Down | Pathway |
| --- | --- | --- | --- | --- | --- | --- | --- |
| dre04912 | K05858 | 62 | 4 | 1 | 0.0014152 | 0.4492123 | GnRH signaling pathway |
| dre04080 | K05226 | 67 | 4 | 2 | 0.0018878 | 0.1338667 | Neuroactive ligand-receptor interaction |
| dre04520 | K05753 | 68 | 3 | 0 | 0.0164555 | 1 | Adherens junction |
| dre04210 | K02649 | 83 | 3 | 1 | 0.0277542 | 0.5504021 | Apoptosis |
| dre04261 | K05858 | 87 | 3 | 1 | 0.0313155 | 0.5674791 | Adrenergic signaling in cardiomyocytes |
| dre04010 | K05095 | 171 | 4 | 3 | 0.04608 | 0.2232692 | MAPK signaling pathway |
| dre03450 | K10884 | 6 | 1 | 0 | 0.0465484 | 1 | Non-homologous end-joining |
| dre04514 | K06088 | 48 | 2 | 0 | 0.0550964 | 1 | Cell adhesion molecules |
| dre04137 | K08341 | 51 | 2 | 0 | 0.0613505 | 1 | Mitophagy - animal |
| dre04110 | K06619 | 60 | 2 | 0 | 0.0814396 | 1 | Cell cycle |
| dre04114 | K04441 | 62 | 2 | 2 | 0.0861485 | 0.1179447 | Oocyte meiosis |
| dre00590 | K07418 | 20 | 1 | 0 | 0.1470196 | 1 | Arachidonic acid metabolism |
| dre03410 | K10798 | 23 | 1 | 0 | 0.1671516 | 1 | Base excision repair |
| dre00410 | K00128 | 23 | 1 | 0 | 0.1671516 | 1 | beta-Alanine metabolism |
| dre00640 | K07514 | 27 | 1 | 0 | 0.1932676 | 1 | Propanoate metabolism |
| dre00480 | K00681 | 32 | 1 | 1 | 0.2247803 | 0.2645558 | Glutathione metabolism |
| dre00970 | K01870 | 34 | 1 | 0 | 0.2370429 | 1 | Aminoacyl-tRNA biosynthesis |
| dre04370 | K02649 | 35 | 1 | 0 | 0.2431024 | 1 | VEGF signaling pathway |
| dre04141 | K10575 | 120 | 2 | 3 | 0.245355 | 0.1066185 | Protein processing in endoplasmic reticulum |
| dre00270 | K08963 | 37 | 1 | 1 | 0.2550794 | 0.2991052 | Cysteine and methionine metabolism |
| dre04142 | K06501 | 90 | 0 | 5 | 1 | 0.0016286 | Lysosome |
| dre00630 | K01915 | 30 | 0 | 2 | 1 | 0.0329055 | Glyoxylate and dicarboxylate metabolism |
| dre00603 | K03663 | 5 | 0 | 1 | 1 | 0.0468059 | Glycosphingolipid biosynthesis - globo and isoglobo series |
| dre00190 | K03967 | 87 | 0 | 3 | 1 | 0.0500817 | Oxidative phosphorylation |
| dre01100 | K00128 | 860 | 3 | 13 | 0.9721356 | 0.0626876 | Metabolic pathways |
| dre00601 | K07970 | 7 | 0 | 1 | 1 | 0.0649164 | Glycosphingolipid biosynthesis - lacto and neolacto series |
| dre01212 | K07514 | 44 | 0 | 2 | 1 | 0.0657289 | Fatty acid metabolism |
| dre03320 | K07514 | 54 | 1 | 2 | 0.3496255 | 0.0936223 | PPAR signaling pathway |
| dre00531 | K12309 | 11 | 0 | 1 | 1 | 0.1001236 | Glycosaminoglycan degradation |
| dre04141 | K10575 | 120 | 2 | 3 | 0.245355 | 0.1066185 | Protein processing in endoplasmic reticulum |
| dre04540 | K04157 | 59 | 0 | 2 | 1 | 0.1086435 | Gap junction |
| dre04114 | K04441 | 62 | 2 | 2 | 0.0861485 | 0.1179447 | Oocyte meiosis |
| dre04080 | K05226 | 67 | 4 | 2 | 0.0018878 | 0.1338667 | Neuroactive ligand-receptor interaction |
| dre01040 | K10203 | 15 | 0 | 1 | 1 | 0.1340209 | Biosynthesis of unsaturated fatty acids |
| dre00650 | K07514 | 15 | 0 | 1 | 1 | 0.1340209 | Butanoate metabolism |
| dre00062 | K01074 | 16 | 0 | 1 | 1 | 0.1422961 | Fatty acid elongation |
| dre04145 | K01365 | 73 | 0 | 2 | 1 | 0.1535658 | Phagosome |
| dre04144 | K05095 | 159 | 2 | 3 | 0.3593518 | 0.1936028 | Endocytosis |
| dre00020 | K00031 | 23 | 0 | 1 | 1 | 0.1980761 | Citrate cycle (TCA cycle) |
| dre00982 | K00699 | 24 | 0 | 1 | 1 | 0.2057464 | Drug metabolism - cytochrome P450 |
| dre01200 | K07514 | 90 | 1 | 2 | 0.5125323 | 0.2118415 | Carbon metabolism |
| dre00980 | K00699 | 25 | 0 | 1 | 1 | 0.2133443 | Metabolism of xenobiotics by cytochrome P450 |
| dre04010 | K05095 | 171 | 4 | 3 | 0.04608 | 0.2232692 | MAPK signaling pathway |
| dre03040 | K12862 | 95 | 0 | 2 | 1 | 0.2294162 | Spliceosome |
| dre00520 | K01183 | 28 | 0 | 1 | 1 | 0.2357097 | Amino sugar and nucleotide sugar metabolism |
| dre00051 | K17497 | 28 | 0 | 1 | 1 | 0.2357097 | Fructose and mannose metabolism |
| dre00480 | K00681 | 32 | 1 | 1 | 0.2247803 | 0.2645558 | Glutathione metabolism |
| dre00260 | K00315 | 36 | 0 | 1 | 1 | 0.2923261 | Glycine, serine and threonine metabolism |
| dre00620 | K00128 | 36 | 0 | 1 | 1 | 0.2923261 | Pyruvate metabolism |
| dre00270 | K08963 | 37 | 1 | 1 | 0.2550794 | 0.2991052 | Cysteine and methionine metabolism |
| dre03050 | K11598 | 37 | 0 | 1 | 1 | 0.2991052 | Proteasome |
| dre00280 | K00128 | 40 | 1 | 1 | 0.2726956 | 0.31906 | Valine, leucine and isoleucine degradation |
| dre00983 | K00699 | 43 | 0 | 1 | 1 | 0.3384534 | Drug metabolism - other enzymes |
| dre04060 | K04304 | 50 | 1 | 1 | 0.3285034 | 0.3816084 | Cytokine-cytokine receptor interaction |
| dre04916 | K05858 | 50 | 0 | 1 | 1 | 0.3816084 | Melanogenesis |
| dre04146 | K07514 | 53 | 0 | 1 | 1 | 0.3992409 | Peroxisome |
| dre04914 | K02649 | 53 | 0 | 1 | 1 | 0.3992409 | Progesterone-mediated oocyte maturation |
| dre04260 | K02265 | 54 | 1 | 1 | 0.3496255 | 0.4050073 | Cardiac muscle contraction |
| dre05132 | K10419 | 152 | 0 | 2 | 1 | 0.4273702 | Salmonella infection |
| dre04012 | K02649 | 58 | 0 | 1 | 1 | 0.4275311 | ErbB signaling pathway |

Supplementary Table 7: List of KEGG pathways for up- and down-regulated pathways for the liver. ID refers to the universal identifiers of the KEGG terms, the N column represents the total number of genes annotated with each KEGG pathway. The Up and Down columns indicate the number of genes within the KEGG pathway that are significantly up- and down-regulated in high versus low treatment, respectively.

| ID | ko | N | Up | Down | *p*- value Up | *p*-value Down | Pathway |
| --- | --- | --- | --- | --- | --- | --- | --- |
| dre03010 | K02973 | 105 | 36 | 0 | 1.09E-20 | 1 | Ribosome |
| dre03320 | K07514 | 54 | 9 | 11 | 0.00174 | 1.461E-05 | PPAR signaling pathway |
| dre00232 | K00365 | 3 | 2 | 0 | 0.0078845 | 1 | Caffeine metabolism |
| dre04210 | K02649 | 83 | 10 | 2 | 0.010869 | 0.8784165 | Apoptosis |
| dre00591 | K07418 | 10 | 3 | 2 | 0.01 | 0.0661561 | Linoleic acid metabolism |
| dre04260 | K02265 | 54 | 6 | 4 | 0.06 | 0.2020317 | Cardiac muscle contraction |
| dre04068 | K08341 | 82 | 8 | 1 | 0.06 | 0.9733577 | FoxO signaling pathway |
| dre00565 | K16342 | 18 | 3 | 1 | 0.06 | 0.5474014 | Ether lipid metabolism |
| dre00230 | K00939 | 84 | 8 | 2 | 0.07 | 0.882582 | Purine metabolism |
| dre00983 | K00699 | 43 | 5 | 5 | 0.07 | 0.0361902 | Drug metabolism - other enzymes |
| dre04115 | K10140 | 33 | 4 | 0 | 0.09 | 1 | p53 signaling pathway |
| dre00534 | K01793 | 11 | 2 | 0 | 0.11 | 1 | Glycosaminoglycan biosynthesis - heparan sulfate / heparin |
| dre00564 | K00968 | 49 | 5 | 4 | 0.11 | 0.1587812 | Glycerophospholipid metabolism |
| dre04150 | K02649 | 83 | 7 | 2 | 0.14 | 0.8784165 | mTOR signaling pathway |
| dre04810 | K05095 | 131 | 10 | 3 | 0.15 | 0.9257988 | Regulation of actin cytoskeleton |
| dre04216 | K14714 | 26 | 3 | 5 | 0.15 | 0.0044765 | Ferroptosis |
| dre00830 | K00699 | 26 | 3 | 1 | 0.15 | 0.6819761 | Retinol metabolism |
| dre00561 | K00128 | 40 | 4 | 1 | 0.15 | 0.8286383 | Glycerolipid metabolism |
| dre04620 | K02649 | 41 | 4 | 0 | 0.16 | 1 | Toll-like receptor signaling pathway |
| dre03008 | K14525 | 56 | 5 | 1 | 0.17 | 0.9155784 | Ribosome biogenesis in eukaryotes |
| dre03050 | K11598 | 37 | 1 | 24 | 0.86 | 1.68E-24 | Proteasome |
| dre01212 | K07514 | 44 | 3 | 12 | 0.41 | 2.05E-07 | Fatty acid metabolism |
| dre04141 | K10575 | 120 | 3 | 19 | 0.95 | 7.513E-07 | Protein processing in endoplasmic reticulum |
| dre01100 | K00128 | 860 | 48 | 63 | 0.33 | 1.397E-05 | Metabolic pathways |
| dre03320 | K07514 | 54 | 9 | 11 | 0.00 | 1.461E-05 | PPAR signaling pathway |
| dre01040 | K10203 | 15 | 0 | 6 | 1.00 | 2.196E-05 | Biosynthesis of unsaturated fatty acids |
| dre00061 | K15013 | 13 | 0 | 5 | 1.00 | 0.000139 | Fatty acid biosynthesis |
| dre00513 | K00736 | 27 | 0 | 6 | 1.00 | 0.0008373 | Various types of N-glycan biosynthesis |
| dre04145 | K01365 | 73 | 3 | 10 | 0.74 | 0.0010644 | Phagosome |
| dre00510 | K00736 | 29 | 0 | 6 | 1.00 | 0.0012493 | N-Glycan biosynthesis |
| dre04142 | K06501 | 90 | 3 | 11 | 0.86 | 0.001586 | Lysosome |
| dre00480 | K00681 | 32 | 2 | 6 | 0.50 | 0.0021372 | Glutathione metabolism |
| dre00982 | K00699 | 24 | 2 | 5 | 0.36 | 0.0031042 | Drug metabolism - cytochrome P450 |
| dre04216 | K14714 | 26 | 3 | 5 | 0.15 | 0.0044765 | Ferroptosis |
| dre00592 | K16342 | 9 | 1 | 3 | 0.38 | 0.0054732 | alpha-Linolenic acid metabolism |
| dre01200 | K07514 | 90 | 4 | 9 | 0.70 | 0.0150273 | Carbon metabolism |
| dre00071 | K00128 | 35 | 3 | 5 | 0.28 | 0.0161108 | Fatty acid degradation |
| dre02010 | K05665 | 24 | 0 | 4 | 1.00 | 0.0181249 | ABC transporters |
| dre00980 | K00699 | 25 | 1 | 4 | 0.74 | 0.0208638 | Metabolism of xenobiotics by cytochrome P450 |
| dre04146 | K07514 | 53 | 4 | 6 | 0.30 | 0.0254687 | Peroxisome |
| dre00062 | K01074 | 16 | 1 | 3 | 0.58 | 0.0291849 | Fatty acid elongation |
| dre00190 | K03967 | 87 | 5 | 8 | 0.48 | 0.0331838 | Oxidative phosphorylation |
| dre00983 | K00699 | 43 | 5 | 5 | 0.07 | 0.0361902 | Drug metabolism - other enzymes |
| dre00590 | K07418 | 20 | 2 | 3 | 0.28 | 0.0523814 | Arachidonic acid metabolism |
| dre00140 | K00699 | 20 | 1 | 3 | 0.66 | 0.0523814 | Steroid hormone biosynthesis |
| dre00350 | K00431 | 20 | 1 | 3 | 0.66 | 0.0523814 | Tyrosine metabolism |
| dre00591 | K07418 | 10 | 3 | 2 | 0.01 | 0.0661561 | Linoleic acid metabolism |
| dre00500 | K01084 | 22 | 0 | 3 | 1.00 | 0.0664734 | Starch and sucrose metabolism |
| dre00620 | K00128 | 36 | 1 | 4 | 0.86 | 0.067376 | Pyruvate metabolism |
| dre00410 | K00128 | 23 | 1 | 3 | 0.71 | 0.0741012 | beta-Alanine metabolism |
| dre00670 | K13403 | 13 | 0 | 2 | 1.00 | 0.1054345 | One carbon pool by folate |
| dre00640 | K07514 | 27 | 0 | 3 | 1.00 | 0.1081439 | Propanoate metabolism |
| dre00330 | K00128 | 28 | 2 | 3 | 0.43 | 0.1174564 | Arginine and proline metabolism |
| dre00600 | K12357 | 28 | 0 | 3 | 1.00 | 0.1174564 | Sphingolipid metabolism |
| dre00524 | K00844 | 3 | 0 | 1 | 1.00 | 0.1236652 | Neomycin, kanamycin and gentamicin biosynthesis |
| dre00650 | K07514 | 15 | 2 | 2 | 0.18 | 0.1342785 | Butanoate metabolism |
| dre00630 | K01915 | 30 | 1 | 3 | 0.80 | 0.1369243 | Glyoxylate and dicarboxylate metabolism |
| dre04144 | K05095 | 159 | 4 | 10 | 0.97 | 0.1474093 | Endocytosis |
| dre00340 | K00128 | 16 | 2 | 2 | 0.20 | 0.1492919 | Histidine metabolism |
| dre00564 | K00968 | 49 | 5 | 4 | 0.11 | 0.1587812 | Glycerophospholipid metabolism |
